# Supplementary material for: Disease characteristics and management of hospitalised adolescents and adults with community-acquired pneumonia in China: a retrospective multicentre survey
Source: BMJ Open. 2018 Feb 15;8(2):e018709. doi: 10.1136/bmjopen-2017-018709 (PMC5829872; doi:10.1136/bmjopen-2017-018709)

Appendix 1: Details of Participating centers

Name of the hospital	Province, city	2nd and 3rd level hospital	Teaching Hospital	Beds	Staff of Clinical Microbioloy Lab
Beijing Chao-Yang Hospital Affiliated to Capital Medical University	Beijing	3rd	Yes	1400	11
Beijing Jishuitan Hospital 4th Medical College of Peking University	Beijing	3rd	Yes	1500	10
Beijing Luhe Hospital Affiliated to Capital Medical University	Beijing	3rd	Yes	1042	5
Qingdao Municipal Hospital	ShanDong, Qingdao	3rd	Yes	1200	4
Qilu Hospital Of Shandong University(Qindao)	ShanDong, Jinan	3rd	Yes	1200	6
Beijing Huimin Hospital	Beijing	2nd	Yes	500	2
Linzi District People's Hospital	ShanDong, Zibo	2nd	Yes	1200	5
The 2nd Hospital of Beijing Corps, Chinese Armed Police Forces	Beijing	3rd	Yes	450	2
China-Japan Friendship Hospital	Beijing	3rd	Yes	1610	9
Yan'an Hospital Affiliated to Kunming Medical University	Kunming, Yan'an	3rd	Yes	1302	4

Yantai Yuhuangding Hospital	Shangdong, Yantai	3rd	Yes	3000	6
Rizhao Chinese Medical Hospital Affiliated to Shandong Chinese Medical University	Shangdong, Rizhao	3rd	Yes	1212	8
Weifang NO.2 People's Hospital	Shangdong, Weifang	3rd	Yes	1006	8

Definition of 2nd and 3rd level hospital in China:

The 2nd level hospital was defined as a hospital providing medical, prevention, health care and rehabilitation services to multiple communities (with a radius of population more than 100,000 peoples); the 3rd level hospital was defined as a hospital providing medical service to the whole country beyond cities and provinces, with comprehensive medical, teaching and research ability.

Appendix 2: ICD-10

Influenza with pneumonia, other influenza virus identified	J10.0
Influenza with pneumonia, virus not identified	J11.0
Virus pneumonia, not elsewhere classified	J12
Adenoviral pneumonia	J12.0
Respiratory syncytial virus pneumonia	J12.1
Parainfluenza virus pneumonia	J12.2
Other virus pneumonia	J12.8
Viral pneumonia,unspecified	J12.9
Pneumonia due to *Streptococcus pneumoniae*	J13
Pneumonia due to *Haemophilus influenzae*	J14
Bacterial pneumonia, not elsewhere classified	J15
Pneumonia due to *Klebsiella pneumoniae*	J15.0
Pneumonia due to *Pseudomonas spp.*	J15.1
Pneumonia due to *Staphylococcus*	J15.2
Pneumonia due to *Streptococcus spp*., group B	J15.3
Pneumonia due to other *streptococci*	J15.4
Pneumonia due to *Escherichia coli*	J15.5
Pneumonia due to other aerobic Gram-negative bacteria	J15.6
Pneumonia due to Mycoplasma pneumoniae	J15.7
Other bacterial pneumonia	J15.8

Bacterial pneumonia, unspecified	J15.9
Pneumonia due to other infectious organisms, not elsewhere classified	J16
Chlamydia pneumonia	J16.0
Pneumonia due to other specified infectious organisms	J16.8
Pneumonia due to other specified infectious organism	J16.8
Pneumonia in diseases classified elsewhere	J17*
Pneumonia in bacterial diseases classified elsewhere	J17.0*
Pneumonia in viral diseases classified elsewhere	J17.1*
Pneumonia in mycoses	J17.2*
Pneumonia in other diseases classified elsewhere	J17.8*
Pulmonary mycobacterial infection	A31.0
Pulmonary actinomycosis	A42.0
Pulmonary nocardiosis	A43.0
Legionnaires' disease	A48.1
Varicella pneumonia	B01.2+
Measles complicated by pneumonia	B05.1+
Cytomegaloviral pneumonitis	B25.0+
Pulmonary candidiasis	B37.1
Acute pulmonary coccidioidomycosis	B38.0
Acute pulmonary histoplasmosis capsulati	B39.0
Acute pulmonary blastomycosis	B40.0

Pulmonary paracoccidioidomycosis	B41.0
Pulmonary sporotrichosis	B42.0+
Invasive pulmonary aspergillosis	B44.0
Other pulmonary aspergillosis	B44.1
Pulmonary cryptococcosis	B45.0
Pulmonary mucormycosis	B46.0
Pneumonia, organism unspecified	J18
Bronchopneumonia, unspecified organism	J18.0
Lobar pneumonia, unspecified	J18.1
Hypostatic, pneumonia, unspecified	J18.2
Other pneumonia, organism unspecified	J18.8
Pneumonia, unspecified	J18.9

Appendix 3: Case Report Form Of Patients Hospitalized With CAP/HCAP

**Code：　　　R-**	
**Name：**	**Gender：　○Male　　○Female**
**Age：　______years old**	**Nationality：　○Han　　○Others**
**Eight：______cm**	**Weight：___ kg**
**ID Number：**	
**Date Of Admission：______Y____M____D**	
**Case Number ：**	**ID Number:**
**Admission Form：　○Outpatience　○Emergency　○Transfers**	
**Tel：**	**Cell Phone：**
**Provider Payments：○Social Medical Insurance　○New Rural Cooperative Medical System　○ Medical Services At State Expense　○ Commercial Medical Insurance　○Self-paying　○Others**	

Study Director：Bin Cao

Team Members：Liang Chen、Hu Li、Meng Liu、Xiudi Han、Xiaoli Zhu 、Bo Liu、Jinxiang Wang、Xuexin Yao、Chunxiao Zhang、Shujing Shi、Fei Zhou、Chunxue Xue、Yanli Li、Donghao Yu （Beijing Chao-Yang Hospital 001；Beijing Jishuitan Hospital 002；Beijing Luhe Hospital 003；Qingdao Municipal Hospital 004;Qilu Hospital Of Shandong University(Qindao) 005；Beijing Huimin Hospital 006；Linzi District People's Hospital 007；The 2nd Hospital of Beijing Corps,Chinese Armed Police Forces 008；China-Japan Friendship Hospital 009;Yan'an Hospital Affliated to Kunming Medical University 010）

Inclusion Criteria：

1. **Age ≥14 years old**
2. **Onset in community**
3. **Chest X-ray or CT scan showing infiltration or interstitial changes, with or without pleural effusion**
4. **Any one of pneumonia clinical manifestations, including：**

(a) Recent cough, sputum or aggravation of respiratory symptoms, the emergence of purulent sputum, with or without chest pain;

(b) Fever (axillary temperature ≥37.3°C) or hypothermia (axillary temperature <36 °C);

(c) Signs of pulmonary consolidation and (or) moist rales;

(d) WBC>10×10^9/L, or <4×10^9/L, with or without nucleus left.

Meet criteria 1,2 ,3 and anyone of criteria 4

Exclusion Criteria：

1. **Lung infiltrate or interstitial changes which can be interpreted as lung cancer, pulmonary tuberculosis, non-infectious interstitial lung diseases, pulmonary edema, atelectasis, pulmonary embolism, pulmonary eosinophil infiltration, pulmonary vasculitis;**
2. **HIV positive**
3. **Readmission within 72 hours after discharging.**

**Muscle And Joint Aches//Headache**	
**Darrhea?**	○Y ○N
**Familial Aggregation（2 Epidemiological Related People Suffered From Pneumonia In Two Weeks）?**	○Y ○N
**Physical Examination (The Worst Value Of The Day On Admission）**	
**Tmax，(**	
**Tmin，(**	
**HR, beats/min**	
**RR, breaths/min**	
**BP(Systolic Pressure / Diastolic Pressure), mmHg**	
**Disorder Of Consciousness?**	○Y ○N
**Cyanosis?**	○Y ○N
**Physical Signs Of Lung:**	**Moist rales** ○Y ○N<br>**Dry rales** ○Y ○N
**Edema Of Legs?**	○Y ○N;<br>**If Y，Asymmetric Edema Of Legs?** ○Y ○N

3.Pre-hospital Medical Data ○Y ○N

**Radiology**		
**Chest X-ray**<br>○Y<br>○N<br>○Unknown<br>**Date of Examination:**<br>____Y___M__D	**Site Of Pneumonia**	○**Bilateral Lung** ○**Unilateral Lung**
	**Site Of Pneumonia**	○**Superior Lobe Of Right Lung**<br>○**Middle Lobe Of Right Lung**<br>○**Inferior Lobe Of Right Lung**<br>○**Superior Lobe Of Left Lung**<br>○**Inferior Lobe Of Left Lung**<br>○**Unknown**
	**Plural effusion**	○N ○Left ○Right ○Bilateral
	**Cavity**	○Y ○N
	**consolidation**	○Y ○N
	**Interstitial Change**	○Y ○N
	**Infiltration**	○Y ○N
**Lung CT**<br>○Y<br>○N<br>○Unknown<br>**Date of Examination:**<br>____Y___M__D	**Alveolar Infiltration**	○**Superior Lobe Of Right Lung**<br>○**Middle Lobe Of Right Lung**<br>○**Inferior Lobe Of Right Lung**<br>○**Superior Lobe Of Left Lung**<br>○**Inferior Lobe Of Left Lung**<br>○**Bilateral Diffuse Infiltration**<br>○**Unilateral Diffuse iInfiltration**

	Plural effusion	○N ○Left ○Right ○Bilateral
	Cavity	○Y ○N
	consolidation	○Y ○N
	Abscesses	○Y ○N
	Patchy Shadow	○Y ○N
	Interstitial change	○Y ○N

Microbiological Examination

Microbiological Examination Before Admission ○Y ○N ○Unknown

If Y：
Date Of Specimen Collection：______Y_____M_____D
Specimen Type: ○Sputum ○Blood ○BALF ○Asopharyngeal Swab
○Endotracheal Aspirate ○Plural Effusion ○Urine
Microbiological Examination Results:________________________________

Treatment Before Admission

Antimicrobials Before Admission ○Y ○N ○Unknown

Drug name（Generic Name And Trade Name）	Route Of Administration	Drug Regimen	Start Time	TerminalTime
eg：Ceftriaxone（罗氏芬）	● Intravenous ○Oral	2.0g ，Qd	2014．9．1	2014．9．8
	○ Intravenous ○Oral			
	○ Intravenous ○Oral			
	○ Intravenous ○Oral			
	○ Intravenous ○Oral			
**Antiviral Drug Use Before Admission ○Y ○N ○Unknown**				
	○Intravenous ○Oral ○ Inhalation			
	○Intravenous ○Oral ○ inhalation			
**Corticosteroid Use Before Admission ○Y ○N ○Unknown**				
	○Intravenous ○Oral ○ inhalation			
	○Intravenous ○Oral ○ inhalation			

Vasopressor Use Before Admission ○Y ○N ○Unknown

If Y，Start Time：_______________ Terminal Time：_______________

Invasive Ventilation Before Admission ○Y ○N ○Unknown

If Y，Start Time：_______________ Terminal Time：_______________

‘ –‡5. Vasopressors：Norepinephrine、Dopamine、Dobutamine、Metaraminol、adrenaline

4.Laboratory Examination In 24hr On Admission

		**Interstitial Change**	**○Y ○N**
		**Alveolar Infiltration**	**○Superior Lobe Of Right Lung**<br>**○Middle Lobe Of Right Lung**<br>**○Inferior Lobe Of Right Lung**<br>**○Superior Lobe Of Left Lung**<br>**○Inferior Lobe Of Left Lung**<br>**○Bilateral Diffuse Infiltration**<br>**○Unilateral Diffuse Infiltration**
**Ultrasonography**	**Lower Limb Vascular Ultrasound Exam**	**Venous Thrombosis**	**○N ○Left ○Right**<br>**○Bilateral ○ Unexamined**

Note※The Worst Value Of Blood Gas Analysis And FiO2 At That Time.

6.Keep Detailed Records Of The Following Time Points,And Write down The Code In The First Row Of The Table：

The 4th day（The Day On Admission Is The 1st Day）； The day of changing Antibiotics in 14 days After Admission； The 14th day after Admission ； The Day Of Discharging

Category	Item(Unit)	The Reason and The Date						
Vital Signs	Disorder Of Consciousness							
	Tmax（ (）							
	Tmin(( )							
	HR（beats/min）							
	RR（breaths/min）							
	BP(/ mmHg）							
Symptoms<br>1. Exacerbation<br>2. Alleviation<br>3. No-change	Cough							
	Sputum							
	Chest Pain							
	Shortness Of Breath							
	Moist Rales							
	Dry Rales							
Blood Routine	WBC（*10^9/L）							
	Neu（*10^9/L）							
	Lym（*10^9/L）							
	HGB（g/L）							
	HCT（%）							
	PLT（*10^9/L）							
Biochemistry	ALB（g/L）							
	LDH（U/L）							
	AST（U/L）							
	ALT（U/L）							
	ALP（U/L）							
	TBIL（umol/L）							
	DBIL（umol/L）							
	CK（U/L）							
	CTNI（ng/ml）							
	BUN（mmol/L）							
	Cr（mmol/L）							
	[illegible]							

Note：a. Direct Microscopy of sputum is not included

7.Treatment During Hospitalization

Antibiotics Use ○Y ○N				
Drug Name （Generic Name And Trade Name）	Route Of Administration	Drug Regimen	Start Time	Terminal Ttime
eg： Ceftriaxone （罗氏芬）	● Intravenous ○Oral	2.0g，Qd	2014-3-2	2014-4-5
	○ Intravenous ○Oral			
	○ Intravenous ○Oral			
	○ Intravenous ○Oral			
	○ Intravenous ○Oral			
	○ Intravenous ○Oral			
	○ Intravenous ○Oral			
	○ Intravenous ○Oral			
**Antiviral Drugs Use ○Y ○N**				
Drug name （Generic Name And Trade Name）	Route Of Administration	Drug Regimen	Start Time	Terminal Time
	○ Intravenous ○Oral ○ Inhalation			
	○ Intravenous ○Oral ○ Inhalation			
	○ Intravenous ○Oral ○ Inhalation			
**Glucocorticoids Use ○Y ○N**				
Drug name （Generic Name And Trade Name）	Route Of Administration	Drug Regimen	Start Time	Terminal Time
	○ Intravenous ○Oral ○Inhalation			
	○ Intravenous ○Oral ○ Inhalation			
**Vasopressors Use ○Y ○N**				
Drug Name	Route Of Administration	Drug Regimen	Start Time	Terminal Ttime
**Immunoregulation Drugs（Including Intravenous Immunoglobulin 、Thymosins）○Y ○N**				
Drug Name	Route of administration	Drug Regimen	Start time	Terminal time

**Alternative/ Supportive Treatment**			
**Item**	**Use**	**Start Time**	**Terminal Time**
**Continuous Venous-venous Hemofiltration**	○Y ○N		
**Extracorporeal Membrane Oxygenation （ECMO）**	○Y ○N		
**Non-invasive Ventilation**	○Y ○N		
**Invasive Ventilation**	○Y ○N		

8、Measurement Of T Lymphocyte Subsets

Date of specimen collection: ___Y ____M ____D

**T lymphocyte subsets**	**CD4**		**/ml**
	**CD8**		**/ml**
	**CD4%**		
	**CD8%**		
	**NK**		**/ml**
	**NKT**		**/ml**
	**CD4/CD8**		

Note：Without Time Limitation

9.Microbiological Examination

(1).Microbiological Examination In 48hrs After Admission ○Y ○N

**Microbiological Examination For Sputum Or eEndotracheal aAspiration<br>Date Of Specimen Collection：__Y___M___D**	
**Item**	**Results**
**Direct Microscopy**	**○Good Quality Sputum（ > 25 leukocytes and < 10 epithelial cells per × 100 magnification field）<br>○Not Good Quality Sputum<br>○Unknown**
	**○G+ Cocci ○G+ Bacillus ○G- Cocci<br>○G- Bacillus ○Positive Acid-fast Stain ○None**

**Bacteria Culture**	**○Streptococcus pneumoniae ○Moraxella catarrhalis<br>○Haemophilus influenzae ○staphylococcus aureus<br>○Pseudomonas aeruginosa ○Klebsiella pneumoniae<br>○Enterobacter cloacae ○Proteus spp<br>○Acinetobacter spp ○Serratia marcescens<br>○Stenotrophomonas maltophilia ○Enterobacter aerogenes<br>○Escherichia coli ○Enterococcus faecalis<br>○Enterococcus faecium ○Others： _____<br>○None Or Normal oropharyngeal flora**
	**Drug Resistant Bacteria**
	**○Methicillin Resistance Staphylococcus aureus（MRSA)<br>○Vancomycin-resistant Enterococcus<br>Bacteria producing ESBLs：<br>○Escherichia coli ○Klebsiella pneumoniae<br>○Enterobacter cloacae ○Serratia marcescens<br>non - fermentative bacteria.：<br>○Acinetobacter baumannii ○Pseudomonas aeruginosa<br>○Others： ____**
	**If Streptococcus pneumoniae ， MIC for penicillin____ug/ml；<br>○Not detected**
	**If MRSA，MIC for Vancomycin_____ug/ml；<br>○Not detected**
**Direct Microscopy**	**○Fungal Spore ○Fungal Hyphae<br>○Cryptococcus neoformans ○None**
**Fungi Culture**	**○Spergillus Fumigatus ○Aspergilusflavus<br>○Aspergillus terreus ○Mucor Mucedo<br>○Candida Spp ○Cryptococcus Neoformans<br>○Undetected ○Others： ____**
**Nucleic Acid Test For Respiratory Virus**	**○Influenza A H1N1 ○Avian influenza H7N9<br>○Influenza A H2N3 ○Influenza A H5N1<br>○Nontypeable Influenza A ○Influenza B<br>○Adenovirus ○Parainfluenza virus 1<br>○Parainfluenza virus2 ○Parainfluenza virus 3<br>○Parainfluenza virus 4 ○Respiratory syncytial virus A<br>○Rhinovirus ○Respiratory syncytial virus B<br>○Coronavirus OC43HKU1 ○Enterovirus<br>○Coronavirus 229ENL63 ○Herpes simplex virus<br>○Bocavirus ○Cytomegalovirus<br>○EB virus ○MERS-CoV**

**Nucleic Acid Test For Atypical Etiology**	**○Mycoplasma pneumoniae ○Chlamydia pneumoniae**<br>**○Legionella spp**

(2).Microbiological Examination For BALF? **○Y ○N**

**Microbiological examination for BALF（Within One Week After Admission）**<br>**Date Of Specimen Collection：__Y___M___D**	
**Item**	**Results**
**Direct Microscopy**	**○G+ Cocci ○G+ Bacillus ○G- Cocci**<br>**○G- Bacillus ○Positive Acid-fast Stain ○None**
**Bacteria Culture**	**○Streptococcus pneumoniae ○Moraxella catarrhalis**<br>**○Haemophilus influenzae ○staphylococcus aureus**<br>**○Pseudomonas aeruginosa ○Klebsiella pneumoniae**<br>**○Enterobacter cloacae ○Proteus spp**<br>**○Acinetobacter spp ○Serratia marcescens**<br>**○Stenotrophomonas maltophilia ○Enterobacter aerogenes**<br>**○Escherichia coli ○Enterococcus faecalis**<br>**○Enterococcus faecium ○Others：_____**<br>**○None Or Normal oropharyngeal flora**
	**Drug Resistant Bacteria**
	**○Methicillin Resistance Staphylococcus aureus（MRSA)**<br>**○Vancomycin-resistant Enterococcus**<br>**Bacteria producing ESBLs：**<br>**○Escherichia coli ○Klebsiella pneumoniae**<br>**○Enterobacter cloacae ○Serratia marcescens**<br>**non - fermentative bacteria.：**<br>**○Acinetobacter baumannii ○Pseudomonas aeruginosa**<br>**○Others：____**
	**If Streptococcus pneumoniae ，MIC for penicillin____ug/ml；**<br>**○Not Detected**
	**If MRSA，MIC for Vancomycin_____ug/ml；**<br>**○Not Detected**
**Direct Microscopy**	**○Fungal Spore ○Fungal Hyphae**<br>**○Cryptococcus neoformans ○None**

**Fungi Culture**	**○Spergillus Fumigatus** **○Aspergilusflavus**<br>**○Aspergillus terreus** **○Mucor Mucedo**<br>**○Candida Spp** **○Cryptococcus Neoformans**<br>**○Undetected** **○Others：____**
**Nucleic Acid For Respiratory Virus**	**○Influenza A H1N1** **○Avian influenza H7N9**<br>**○Influenza A H2N3** **○Influenza A H5N1**<br>**○Nontypeable Influenza A** **○Influenza B**<br>**○Adenovirus** **○Parainfluenza virus 1**<br>**○Parainfluenza virus2** **○Parainfluenza virus 3**<br>**○Parainfluenza virus 4** **○Respiratory syncytial virus A**<br>**○Rhinovirus** **○Respiratory syncytial virus B**<br>**○Coronavirus OC43HKU1** **○Enterovirus**<br>**○Coronavirus 229ENL63** **○Herpes simplex virus**<br>**○Bocavirus** **○Cytomegalovirus**<br>**○EB virus** **○MERS-CoV**
**Nucleic Acid Test For Atypical Etiology**	○Mycoplasma pneumoniae **○Chlamydia pneumoniae**<br>○L**egionella** spp

(3).Blood Culture In One Week After Admission？ ○**Y** ○**N**

**Blood Culture (In One Week After Admission)<br>Date Of Specimen Collection：__Y___M___D**	
**Item**	**Results**
**Bacteria Culture**	**○Staphylococcus aureus** **○Moraxella catarrhalis**<br>**○Haemophilus influenzae** **○Pseudomonas aeruginosa**<br>**○Klebsiella pneumoniae** **○Enterobacter cloacae**<br>**○Proteus spp** **○Acinetobacter spp**<br>**○Serratia marcescens** **○Stenotrophomonas maltophilia**<br>**○Enterobacter aerogenes** **○Escherichia coli**<br>**○Enterococcus faecalis** **○Enterococcus faecium**<br>**○Others：__________** **○None or normal oropharyngeal flora**
	**Drug Resistant Bacteria**
	**○Methicillin resistance staphylococcus aureus（MRSA)**<br>**○Vancomycin-resistant Enterococcus**<br>**Bacteria producing ESBLs：**<br>**○Escherichia coli** **○Klebsiella pneumoniae**<br>**○Enterobacter cloacae** **○Serratia marcescens**<br>**non - fermentative bacteria.：**<br>**○Acinetobacter baumannii** **○Pseudomonas aeruginosa**<br>**○Others：____**

	**If Streptococcus pneumoniae ，MIC for penicillin____ug/ml；**<br>**○Not Detected**
	**If MRSA，MIC for Vancomycin_____ug/ml；**<br>**○Not Detected**
**Fungi Culture**	**○Candidiasis albicans ○Candida krusei**<br>**○Candida tropicalis ○Candida glabrata**<br>**○Candida parapsilosis ○Cryptococcus neoformans**<br>**○Aspergillus fumigatus ○Aspergilus flavus**<br>**○Aspergillus terreus ○Mucor Mucedo**<br>**○Undetected ○Others：____**

（4）、Pleural Effusion？ ○Y ○N

Pleural Effusion Test？ ○Y ○N

**Microbiological Examination For Pleural Effusion（Without Time Limitation）**<br>**Date of Specimen collection：__Y___M___D**	
**Pleural Effusion Routine**	
**Total Cell Count：___×10^6/L；Multinuclear Cell：___×10^6/L；**<br>**Mononuclear Cells：___×10^6/L**	
**Pleural Effusion Biochemistry**	
**LDH：___U/L； ADA：___U/L； Pr：___g/L**<br>**Glu:___mmol/L Cl: ___mmol/L**	
**Item**	**Results**
**Bacteria Culture**	**○Staphylococcus aureus ○Moraxella catarrhalis**<br>**○Haemophilus influenzae ○Pseudomonas aeruginosa**<br>**○Klebsiella pneumoniae ○Enterobacter cloacae**<br>**○Proteus spp ○Acinetobacter spp**<br>**○Serratia marcescens ○Stenotrophomonas maltophilia**<br>**○Enterobacter aerogenes ○Escherichia coli**<br>**○Enterococcus faecalis ○Enterococcus faecium**<br>**○Others：___________ ○None or Normal Oropharyngeal Flora**
	**Drug Resistant Bacteria**
	**○Methicillin resistance staphylococcus aureus（MRSA)**<br>**○Vancomycin-resistant Enterococcus**<br>**Bacteria producing ESBLs：**<br>**○Escherichia coli ○Klebsiella pneumoniae**<br>**○Enterobacter cloacae ○Serratia marcescens**<br>**non - fermentative bacteria.：**<br>**○Acinetobacter baumannii ○Pseudomonas aeruginosa**<br>**○Others：____**
**Fungi Culture**	**○Candidiasis albicans ○Candida krusei**<br>**○Candida tropicalis ○Candida glabrata**

	○Candida parapsilosis ○Cryptococcus neoformans<br>○Aspergillus fumigatus ○Aspergilus flavus<br>○Aspergillus terreus ○Mucor Mucedo<br>○Undetected ○Others：_

（5）、Antigen Test In 48hr After Admission？ ○Y ○N

**Urinary antigen（in 48hr after admission）<br>Date of specimen collection：__Y___M___D**	
**Urinary Antigen For**	**○Positive ○Negative ○Undetected**
**Urinary Antigen For**	**○Positive ○Negative ○Undetected**
**Throat Swab Aantigen Test（In 48hr After Admission）<br>Date Of Specimen Collection：__Y___M___D**	
**Respiratory Syncytial Virus Antigen Test**	**○Positive ○Negative ○Undetected**
**Influenza A Antigen Test**	**○Positive ○Negative ○Undetected**
**Influenza B Antigen Test**	**○Positive ○Negative ○Undetected**

（6）、Antibody Test？ a）○Y ○N

b）If Y , Titer Of Antibody In Paired Serum? ○Y ,Interval___days

○N

**Antibody Test（Without Time Limitation）<br>Date Of Specimen Collection：__Y___M___D**	
**○IgM for**	**○IgM for Influenza A**
**○IgG for**	**○IgM for Parainfluenza**
**○IgM for spp**	**○IgM for Q fever**
**○IgG for spp.**	**○IgM for Adenovirus**
**○IgM for**	**○IgM for Respiratory syncytial virus**
**○IgG for**	**○IgM for Parainfluenza 1,2,3**

10、**Outcomes**

(1).Treatment Failure Within 14 Days

**Treatment Failure Within 14 Days（Multiple choices）**<br>（The Value Of The 1st Day On Admission As The Baseline Data）	
**1.Needs Invasive Ventilation**	○Y ○N
**2.Needs Non-invasive Ventilation**	○Y ○N
**3.Needs Vasopressors**	○Y ○N
**4.Death**	○Y ○N

**The Reasons For Treatment Failure**	
**1.CAP Progression**	**Pneumonia Progression ○Y ○N**
**2.CAP Complications**	**Pyothorax ○Y ○N**<br>**Endocarditis ○Y ○N**<br>**Meningitis ○Y ○N**<br>**Others：__________**
**3.Severe Sepsis Due To CAP**	**ARDS ○Y ○N**<br>**Sepsis ○Y ○N**<br>**Hepatic Failure ○Y ○N**<br>**Renal Ffailure ○Y ○N**<br>**Clotting Disorders, ○Y ○N**<br>**Encephalopathy ○Y ○N**<br>**Others：__________**
**4.Complications Or Underlying Disease Deterioration**	**Pulmonary Embolism ○Y ○N**<br>**Myocardial Infarction ○Y ○N**<br>**Arrhythmia ○Y ○N**<br>**Gastrointestinal Bleeding ○Y ○N**<br>**Congestive Heart Failure ○Y ○N**<br>**COPD ○Y ○N**<br>**Diabetes Mellitus ○Y ○N**<br>**Nephropathy ○Y ○N**<br>**Others：__________**
**5.Complications Due To Treatment**	**Hemopneumothorax ○Y ○N**<br>**Allergic To Antibiotics ○Y ○N**<br>**HAP/VAP ○Y ○N**<br>**Vascular Catheter Infection ○Y ○N**<br>**C. Difficile Infection ○Y ○N**<br>**Iatrogenic Urinary Tract Infection ○Y ○N**<br>**Others：__________**
**6.Unknown**	**○Y ○N**

(2). Complications During Hospitalization

Complications During Hospitalization	
Complications（Multiple Choices） ○Y ○N	
Respiratory Failure ○Y ○N	ARDS ○Y ○N
Heart Failure ○Y ○N	Acute Myocardial Infarction ○Y ○N
Acute Liver Failure ○Y ○N	AcuteRenal Failure ○Y ○N
Septic Shock ○Y ○N	Stroke ○Y ○N
DIC ○Y ○N	Antibiotic Associated Diarrhea ○Y ○N
Arrhythmia ○Y ○N	MODS ○Y ○N
Pulmonary Embolism ○Y ○N	Deep Venous Thrombosis ○Y ○N
Ventilator Associated Pneumonia ○Y ○N	Gastrointestinal Bleeding ○Y ○N
Invasive Aspergillosis ○Y ○N	Mediastinal Emphysema ○Y ○N
Pneumothorax ○Y ○N	Nosocomial Bloodstream Infection ○Y ○N
Others ○Y ○N	If Y：________

(3). Outcomes

Clinical Stability Before Discharge	○Y ○N<br>If Y，the date of clinical stability ______ Y ______M ______D.<br>Meet the following seven criteria：Temperature<37.8 (for more than 24hr；Heart rate ≤100 beats/min ；Respiratory rate ≤24 breaths/min ;Systolic blood pressure ≥90 mm Hg ；Arterial oxygen saturation ≥90% or pO2 ≥ 60 mm Hg on room air ；Ability to maintain oral intake；Normal mental status.
Admitted to RICU/ICU?	○Y ○N<br>If Y，The Date Of Admitted To RICU/ICU：____Y____M____D<br>The Date Of Transfer From RICU/ICU：____Y____M____D
Discharging	The Date Of Discharging____Y____M____D<br>Outcome ○Improvement ○Against-advice discharge ○Death<br>If death，The Death Date_____Y____M____D
Direct Cause Of Death (only one choice)	○Severe Pneumonia ○Respiratory Failure<br>○Shock○Heart Failure ○Acute Myocardial Infarction

	○**Acute renal Failure**<br>○**DIC**<br>○**Gastrointestinal Bleeding**	○**Hepatic failure**<br>○**Stroke**<br>○**Others：________**

10. **Cost And Economy Data**

Total Expenses _______Yuan：

Drugs Cost：__________Yuan ，Antimicrobials Cost _________Yuan

Laboratory Testing Expenses：________Yuan

Bed Charge：__________Yuan

Health Care Worker Labor Cost：_________Yuan

Appendix 4: Definition of underlying diseases

1) Long-term smoking was defined as: cigarette smokers of 10 cigarettes/d during at least the previous year；

2) Alcoholism was defined as: drinking more than 5 bottles of beer (500ml / bottle) or half a catty liquor once in 2 weeks; or drinking more than 2.5 bottles of beer (500ml / bottle) or 2 ounc of white spirit per day for more than five years；

3) Long-term oral corticosteroids was defined as: oral prednisone ≥10mg / d or equivalent doses of other corticosteroids for more than 3 weeks.[1]

4) Long-term inhaled corticosteroids was defined as: inhaled corticosteroid for more than 30 days, the daily dose wasn't limited;

5) COPD was defined as: persistent airflow limitation, FEV1 / FVC < 70% post bronchodilator;

6) Asthma was defined by the history of respiratory symptoms such as wheeze, cough that varied over time and intensity, together with variable respiratory airway limitation;

7) Hypertension was defined as systolic blood pressure≥140mmHg and /or diastolic blood pressure≥90mmHg in resting status;

8) Coronary heart disease included angina pectoris, myocardial infarction, ischemic cardiomyopathy;

9) Chronic congestive heart failure was defined as cardiomegaly and ejection fraction ≤40%;

10) Cerebrovascular diseases included transient ischemic attack, cerebral hemorrhage, subarachnoid hemorrhage, cerebral infarction, etc;

11) Diebetes mellitus: included diabetes mellitus type 1 and diabetes mellitus type 2, not included impaired glucose tolerance and impaired fasting glycaemia;

12) Chronic liver disease included chronic viral hepatitis, chronic alcoholic liver disease, chronic fatty liver disease, etc;

13) Chronic kidney disease included diabetic nephropathy, hypertensive renal damage, chronic glomerulonephritis, chronic pyelonephritis, lupus nephritis, IgA nephropathy, nephrotic syndrome, hereditary kidney disease, etc;

14) Connective Tissue Diseases include SLE, Sjogren's syndrome, rheumatoid arthritis, polymyositis / dermatomyositis, systemic vasculitis, ankylosing spondylitis, inflammatory bowel disease, hyperthyroidism, etc;

15) Organ transplantation or bone marrow transplantation included solid organ transplantating, such as liver transplantation, kidney transplantation, lung transplantation or pancreas transplantation, etc and bone marrow transplantation;

16) Aspiration risk factors included choking, drowning, nasal, pseudobulbar palsy, dementia, coma, poisoning, Parkinson's disease.

17) Immunosuppressive therapy: was defined as systmatic glucocorticosteroid (such as prednisone ≥10mg/d for more than 3 weeks in the last month); cyclosporine or azathioprine use within 3 months, and methotrexate use ≥12.5mg/week within 3 months; biological modifiers such as etanercept and infiximab within 3 weeks.

18) Immunocompromised status included HIV(+)，chemotherapy/radiotherapy within 6 months, immunosuppressive therapy, organ/bone marrow transplantation, splenectomy, hematological neoplasms.[2]

19) Risk factors for pseudomonal infection was defined as chronic airway disease (bronchiectasis or COPD) and HCAP risk factors according to IDSA/ATS criteria.[3-7]

20) Overtreatment was defined as: i) use of antipseudomonal β-lactams or β-lactams+ fluoquinolones in hospitalized (not in ICU) patients without risk factors for pseudomonal infection; i i) use of β-lactams(antipseudomonal or not)+ fluoquinolones in ICU patients aged< 65yr without risk factors for pseudomonas infection；iii) use of anti-MRSA drugs in hospitalized (not in ICU) patients.[3]

References:

1. Jouneau S, Poineuf JS, Minjolle S, *et al*. Which patients should be tested for viruses on bronchoalveolar lavage fluid? *Eur J Clin Microbiol Infect Dis* 2013 ;32:671-7.

2. Sousa D, Justo I, Domínguez A,*et al*. Community-acquired pneumonia in immunocompromised older patients: incidence, causative organisms and outcome. *Clin Microbiol Infect* 2013 ;19:187-92

3. Guidelines for the diagnosis and treatment of adult community-acquired pneumonia in China(2016 Edition). *Zhonghua Jie He He Hu Xi Za Zhi* 2016; 39: 241-2

4. American Thoracic Society.Infectious Diseases Society of America.Guidelines for the management of adults with hospital-acquired, ventilator-associated, and healthcare-associated pneumonia.*Am J Respir Crit Care Med* 2005; 171:388-416

5. Maruyama T1, Fujisawa T, Okuno M, *et al*.

A new strategy for healthcare-associated pneumonia: a 2-year prospective multicenter cohort study using risk factors for multidrug-resistant pathogens to select initial empiric therapy. *Clin Infect Dis* 2013;57:1373-83.

6. Qi F, Zhang GX, She DY *et al*. Healthcare-associated Pneumonia: Clinical Features and Retrospective Analysis Over 10 Years. *Chin Med J (Engl)* 2015;128:2707-13.

7. Seong GM, Kim M, Lee J, *et al*. Healthcare-Associated Pneumonia among Hospitalized Patients: Is It Different from Community Acquired Pneumonia? *Tuberc Respir Dis (Seoul)* 2014;76:66-74.

Appendix 5 Empirical antimicrobial regimens according to Chinese CAP guideline

**Populations**	**Common pathogens**	**Anti-infective agents for initial empirical therapy**	**Comment**
Outpatient treatment (Oral administration is recommended)			
Young adults without underlying disease(s)	*S. pneumoniae*, *M. pneumoniae*, *H. influenzae*, *C. pneumoniae*, influenza virus, adenovirus, *M. catarrhalis*	(1) Aminopenicillins, penicillins-β-lactamase -inhibitor combinations; (2) I or II generation cephalosporins; (3) doxycycline or minocycline; (4) respiratory quinolones; (5) macrolides	(1) Differentiate among bacterial pneumonia, *Mycoplasma*, *Chlamydia* and viral pneumonia based on clinical characteristics; (2) Mild pneumonia caused by *Mycoplasma*, *Chlamydia*, and virus is usually self-limited
Patients with underlying disease(s) or elderly patients (age ≥ 65 years)	*S. pneumoniae*, *H. influenzae*, *Enterobacteriaceae* such as *K. pneumoniae*, *C. pneumoniae*, influenza virus, RSV, *M. catarrhalis*	(1) Penicillins-β-lactamase-inhibitor combinations; (2) II or III generation cephalosporins (oral); (3) respiratory quinolones; (4) penicillins-lactamase -inhibitor combinations, II generation cephalosporins, III generation cephalosporins combined with doxycycline or minocycline or macrolides	Monotherapy with doxycycline or minocycline or macrolides is not recommended in patients with risk factors of resistant *S. pneumoniae* (1), such as age > 65 years, underlying diseases (chronic cardiac, pulmonary, or renal diseases, diabetes mellitus, and immunosuppression), alcoholism, and β-lactams treatment within 3 months.
Inpatient treatment, non-ICU (Intravenous or oral administration)			

Young adults without underlying disease(s)	*S. pneumoniae*, *H. influenzae*, *M. catarrhalis*, *S. aureus*, *M. pneumoniae*, *C. pneumoniae*, influenza virus, adenovirus, other respiratory tract viruses	(1) Penicillin G, aminopenicillins, penicillins-β-lactamase-inhibitor combinations; (2) II or III generation cephalosporins, cephamycins, oxacephems; (3) the above drugs combined with doxycycline, minocycline or macrolides; (4) respiratory quinolones; (5) macrolides	(1) Only 1.9% the *S. pneumoniae* isolates from adult CAP are resistant to intravenous penicillins in China. The percentage of intermediate strains is only about 9%. Intravenous penicillins are still effective in hospitalized patients infected with penicillin-intermediate *S. pneumoniae* when increasing the dosage (23, 161); (2) When atypical pathogens are suspected, doxycycline or minocycline or respiratory quinolones are preferred. Macrolides can be used in regions with lower resistance rate to mycoplasma
Patients with underlying disease(s) or elderly patients (age ≥ 65 years)	*S. pneumoniae*, *H. influenzae*, *Enterobacteriaceae* such as *K. pneumoniae*, influenza virus, RSV, *M. catarrhalis*, anaerobic bacteria, *Legionella*	(1) Penicillins-β-lactamase-inhibitor combinations; (2) III generation cephalosporins or their enzyme-inhibitor combinations, carbapenems such as cephamycins, oxacephems, ertapenem; (3) monotherapy of the above drugs or in combination with macrolides; (4) respiratory quinolones	(1) *Enterobacteriaceae* infection must be considered in patients with underlying disease(s) and elderly patients. The patients must be further evaluated for the risk of infection with ESBLs-producing *Enterobacteriaceae*; (2) Elderly patients should be monitored for the risk factors of aspiration
Requirement for ICU admission (Intravenous administration is recommended)			
Young adults without underlying disease(s)	*S. pneumoniae*, *S. aureus*, influenza virus, adenovirus, *Legionella*	(1) Penicillins-β-lactamase-inhibitor combinations, III generation cephalosporins, cephamycins, oxacephems, ertapenem combined with macrolides; (2) respiratory quinolones	(1) *S. pneumoniae* is the most common pathogen. The other pathogens such as *S. aureus*, *Legionella*, influenza virus should also be considered (1, 2, 162-166); (2) During influenza seasons, attention must be paid to influenza viral infections. Combination with neuraminidase inhibitors should be considered. Attention should be paid to secondary *S. aureus* infection (167). The agents active against MRSA can be used in combination if necessary

Patients with underlying disease(s) or elderly patients (age ≥ 65 years)	*S. pneumoniae*, *Legionella*, *Enterobacteriaceae* such as *K. pneumoniae*, *S. aureus*, anaerobic bacteria, influenza virus, RSV	(1) Penicillins-β-lactamase-inhibitor combinations, III generation cephalosporins or in combination with beta-lactamase inhibitors, carbapenems such as ertapenem combined with macrolides; (2) penicillins-β-lactamase-inhibitor combinations, III generation cephalosporins or in combination with beta-lactamase inhibitors, carbapenems such as ertapenem combined with respiratory quinolones	(1) Evaluate the risk of infection with ESBLs-producing *Enterobacteriaceae*; (2) Physicians should be aware of the risk factors for aspiration and antimicrobial coverage of relevant pathogens
CAP with risk factors for *P. aeruginosa* infection and requirement for inpatient treatment or ICU admission (Intravenous administration is recommended)			
Patients with structural lung disease	*P. aeruginosa*, *S. pneumoniae*, *Legionella*, *Enterobacteriaceae* such as *K. pneumoniae*, *S. aureus*, anaerobic bacteria, influenza virus, RSV virus	(1) β-lactams with antipseudomonal activity; (2) quinolones with antipseudomonal activity; (3) β-lactams with antipseudomonal activity combined with quinolones or aminoglycosides with antipseudomonal activity; (4) combination of β-lactams, aminoglycosides and quinolones with antipseudomonal activity	Risk factors include: (1) airway *P. aeruginosa* colonization; (2) repeated doses of antibacterial drugs or glucocorticoids due to chronic airway disease. Combination therapy is recommended for patients with severe CAP or proven antimicrobial resistance

Appendix 6: Definition of microbiological criteria of CAP:

Definite, if one of the following criteria was met:

4. Positive urinary antigen for *Legionella pneumophila* (LP, Binax Now L pneumophila urinary antigen test; Trinity Biotech, Bray, Ireland);
5. Positive urinary antigen for *Streptococcus pneumoniae* (Binax Now *S pneumoniae* urinary antigen test; Emergo Europe, The Netherlands);
6. Positive bacterial culture from blood or plural fluid except for coagulase negative *Staphylococcus spp*.
7. Paired sera with a fourfold or more increase in the titers of antibodies to *Mycoplasma pneumoniae* (MP), *Chlamydia pneumonia*, *L pneumophila or* respiratory viruses (Influenza A and B, Parainfluenza, Adenovirus, Respiratory syncytial virus). Or Serum IgM antibody (MIF) ≥ 1:16 for *Chlamydia pneumonia.*

Probable, if one of the following criteria was met:

a. Detection of respiratory virus in sputum/bronchoalveolar lavage (BALF)/throat swabs by Realtime-PCR (Zhijiang, Shanghai, China) according to manufacturer's instructions, including respiratory syncytial virus (RSV) types A and B, influenza virus (IFV) types A and B, parainfluenza virus (PIV) types 1, 2, 3 and 4, rhinovirus (HRV), enterovirus (EV), coronavirus (hCoV) types 229E, NL63, OC43 and HKU1, parapneumovirus (hMPV), and adenovirus (AdV), bocavirus;
b. Bacteria isolated form purulent sputum (defined as an adequate quality sputum sample with > 25 leukocytes and < 10 epithelial cells per × 100 magnification field) with compatible findings of Gram staining;
c. Detection of *Mycoplasma pneumoniae* (MP), *Chlamydia pneumonia* or *L pneumophila* in sputum/BALF/throat swabs by Realtime-PCR (Zhijiang, Shanghai, China)
d. Positive antigen for Influenza A/B (Alere ^{TM}, Clearview Exact Influenza A& B)

e. Serum IgM antibody positive for *Mycoplasma pneumoniae* (MP), or Serum IgG antibody (MIF) ≥ 1:512 for *Chlamydia pneumonia.*

Appendix 7: CAP patients with definite and probable microbiological diagnosis

Etiology	Without risk factors for pseudomonal infection (n=409)				With risk factors for pseudomonal infection (n=329)	Total (n=738)
	age<65yr and not in ICU (n=182)	age<65yr and in ICU (n=29)	age≥65yr and not in ICU (n=162)	age≥65yr and in ICU (n=36)		
**Bacterial**	142 (19.2%)	14 (1.9%)	137 (18.6%)	34 (4.6%)	316 (42.8%)	643 (87.1%)
Pseudomonas aeruginosa	27	0	31	6	133	197
Klebsiella pneumoniae	30	9	27	10	54	130
E. coli	15	1	17	2	31	66
Acinetobacter	13	3	20	3	23	62
Staphylococcus aureus	7	3	10	7	24	51
Enterobacter cloacae	9	1	8	3	17	38
Streptococcus pneumoniae	9	1	5	1	9	25
Stenotrophomonas	8	1	10	2	4	25
Enterococcus faecalis	5	0	3	0	9	17
Enterococcus faecium	3	0	1	0	5	9
others	20	3	18	7	35	83
**Atypical etiology**	5 (0.7%)	0 (0.0%)	2 (0.3%)	0 (0.0%)	0 (0.0%)	7 (0.9%)
Mycoplasma pneumoniae	6	0	1	0	0	7
Legionnella pneumoniae	0	1	2	0	0	3
Chlamydia pneumoniae	0	1	0	0	0	1
**Virus**	30 (4.1%)	8 (1.1%)	15 (2.0%)	1 (0.1%)	9 (1.2%)	63 (8.5%)
Influenza A virus	25	8	14	1	6	54
Rhinovirus	3	2	2	0	1	8
Influenza B virus	0	0	4	1	3	8
Adenovirus	6	1	0	0	0	7
Respiratory syncytial virus	1	0	0	0	0	1
Human metapneumovirus	0	0	1	0	0	1
Cytomegalovirus	1	1	0	0	0	2
**Bacterials+viruses**	4 (0.5%)	5 (0.7%)	6 (0.8%)	1 (0.1%)	4 (0.5%)	20 (2.7%)
**Viruses+atypical pathogens**	2 (0.3%)	2 (0.3%)	1 (0.1%)	0 (0.0%)	0 (0.0%)	5 (0.7%)

Appendix 8: Sub-group analysis of 30-day mortality

Item	30-day mortality	P value
Severity of illness		
CURB-65		<0.001
0’	63 (2.7%)	
1’	93 (4.2%)	
2’	64 (7.2%)	
3’	15 (10.2%)	
4’	1 (5.0%)	
5’	1 (100.0%)	
PSI risk class		<0.001
Ⅰ	26 (2.3%)	
Ⅱ	27 (3.0%)	
Ⅲ	24 (3.2%)	
Ⅳ	45 (7.0%)	
Ⅴ	22 (12.2%)	
Age		>0.05
14~64 ys	108 (3.9%)	
65~74 ys	44 (4.1%)	
75~89 ys	81 (4.6%)	
≥90 ys	13 (7.0%)	
Gender		>0.05
Male	144 (4.6%)	
Female	102 (3.8%)	
Underlying Diseases		
None of any underlying disease	47 (2.9%)	
Chronic congestive heart failure	14 (6.9%)	<0.001

COPD	51 (6.4%)	<0.001
Malignant solid tumors	15 (5.9%)	<0.001
Chronic Renal diseases	11 (5.5%)	<0.001
Cerebrovascular Diseases	42 (4.7%)	<0.001
Connective Tissue Diseases	5 (4.5%)	0.003
Coronary Heart Diseases	50 (4.3%)	<0.001
Bronchiectasis	27 (4.3%)	<0.001
Hypertension	87 (4.2%)	<0.001
Asthma	14 (4.1%)	<0.001
Diabetes	36 (3.9%)	<0.001
Chronic Liver diseases	2 (2.2%)	>0.05
ICU admission		
Yes	56 (15.3%)	<0.001
No	190 (3.5%)	
Systemic glucocorticosteroids use in admission		
Yes	87 (5.6%)	<0.001
No	159 (3.7%)	

Appendix Figure 1 Patient screening algorithm for hospitalized CAP

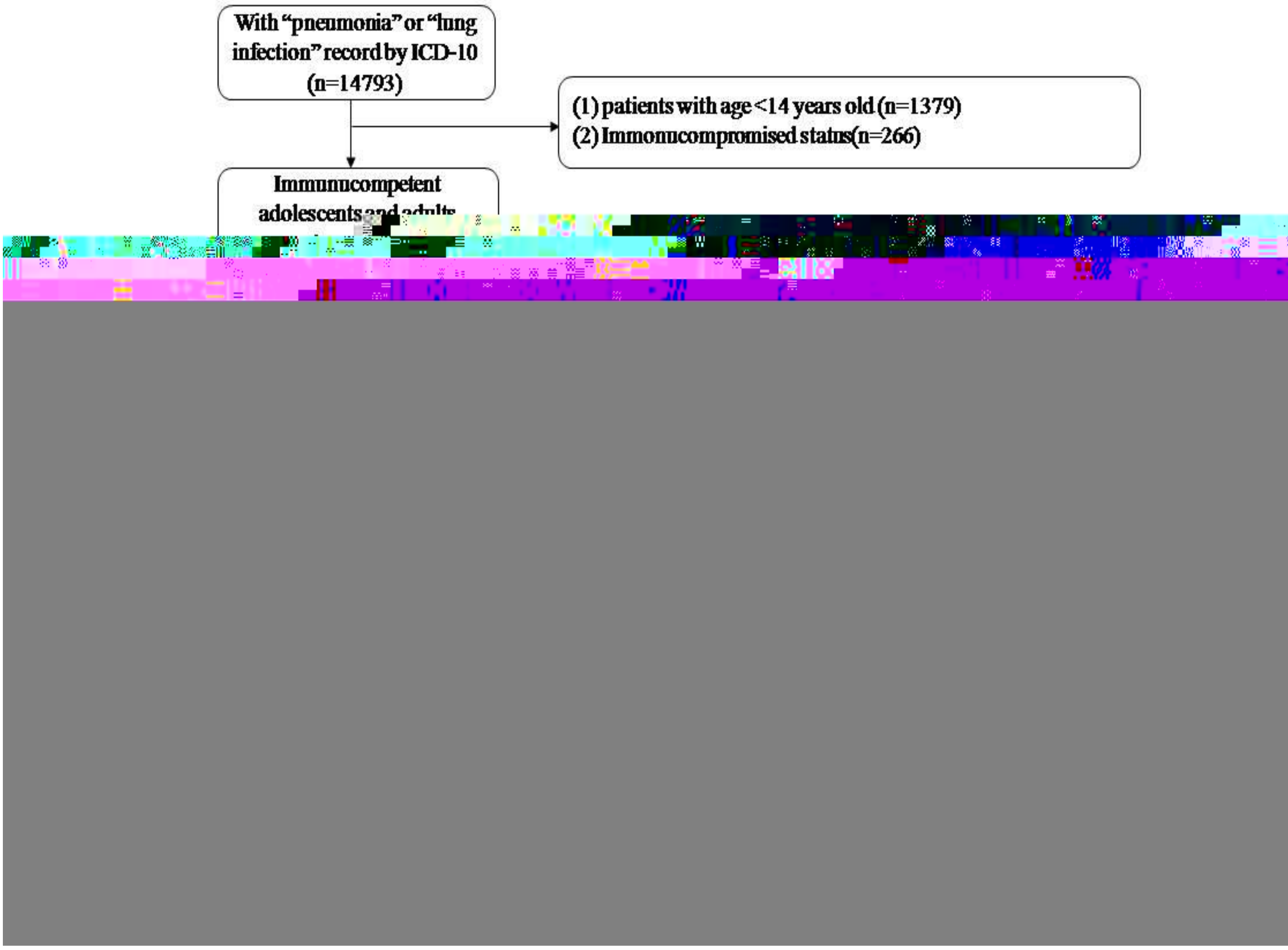

Supplement: Supplementary file 1 [file bmjopen-2017-018709supp001.pdf]
