# Supplementary material for: Disease characteristics and management of hospitalised adolescents and adults with community-acquired pneumonia in China: a retrospective multicentre survey
Source: BMJ Open. 2018 Feb 15;8(2):e018709. doi: 10.1136/bmjopen-2017-018709 (PMC5829872; doi:10.1136/bmjopen-2017-018709)
Supplement: Author's manuscript [file bmjopen-2017-018709.draft_revisions.pdf]

BMJ Open

BMJ Open is committed to open peer review. As part of this commitment we make the peer review history of every article we publish publicly available.

When an article is published we post the peer reviewers' comments and the authors' responses online. We also post the versions of the paper that were used during peer review. These are the versions that the peer review comments apply to.

The versions of the paper that follow are the versions that were submitted during the peer review process. They are not the versions of record or the final published versions. They should not be cited or distributed as the published version of this manuscript.

BMJ Open is an open access journal and the full, final, typeset and author-corrected version of record of the manuscript is available on our site with no access controls, subscription charges or pay-per-view fees (http://bmjopen.bmj.com).

If you have any questions on BMJ Open's open peer review process please email editorial.bmjopen@bmj.com

BMJ Open

Disease characteristics and management of hospitalized adolescents and adults with Community-Acquired Pneumonia in China

Journal:	*BMJ Open*
Manuscript ID	bmjopen-2017-018709
Article Type:	Research
Date Submitted by the Author:	17-Jul-2017
Complete List of Authors:	Chen, Liang; Department of Infectious Diseases and Clinical Microbiology, Beijing Chao-Yang Hospital, Capital Medical University<br>Zhou, Fei; Department of Infectious Diseases and Clinical Microbiology, Beijing Chao-Yang Hospital, Capital Medical University<br>Li, Hui; Department of Infectious Diseases and Clinical Microbiology, Beijing Chao-Yang Hospital, Capital Medical University<br>Xing, Xiqian; Department of Respiratory Medicine, Yan'an Hospital Affiliated to Kunming Medical University<br>Han, Xiudi<br>Wang, Yiming<br>Zhang, Chunxiao<br>Suo, Lijun<br>Wang, Jingxiang<br>Yu, Guohua<br>Wang, Guangqiang<br>Xiao, Ying<br>Yu, Hongxia<br>Wang, Lei<br>Meng, Liu<br>Xue, Chunxue<br>Liu, Bo<br>Zhu, Xiaoli<br>Li, Yanli<br>Xiao, Ying<br>Cui, Xiaojing<br>Li, Lijuan<br>Uyeki, Timothy; CDC<br>Wang, Chen<br>Cao, Bin
**Primary Subject Heading**:	Infectious diseases
Secondary Subject Heading:	Medical management
Keywords:	Community-acquired pneumonia, Disease characteristics, management, China

For peer review only

Disease characteristics and management of hospitalized adolescents and adults with Community-Acquired Pneumonia in China

Liang Chen[1,2], Fei Zhou[1], Hui Li[1], Xiqian Xing[3], Xiudi Han[4], Yimin Wang[5], Chunxiao Zhang[6], Lijun Suo[8], Jinxiang Wang[9], Guohua Yu[11], Guangqiang Wang[12], Xuexin Yao[13], Hongxia Yu[14], Lei Wang[7], Meng Liu[1], Chunxue Xue[1], Bo Liu[8], Xiaoli Zhu[10], Yanli Li[1], Ying Xiao[1], Xiaojing Cui[5], Lijuan Li[5], Timothy M. Uyeki[15], Chen Wang[5, 16], Bin Cao[5, 16] for CAP-China network

1. Department of Infectious Diseases and Clinical Microbiology, Beijing Chao-Yang Hospital, Capital Medical University, Beijing

2. Department of Infectious Disease, 4th Medical College of Peking University, Beijing Jishuitan Hospital, Beijing

3. Department of Respiratory Medicine, Yan'an Hospital Affiliated to Kunming Medical University, Kunming City, Yunnan Province

4. Department of Respiratory Medicine, Qingdao Municipal Hospital, Qingdao City, Shandong Province

5. Center for Respiratory Diseases, China-Japan Friendship Hospital; Department of Pulmonary and Critical Care Medicine, China-Japan Friendship Hospital; National Clinical Research Center of Respiratory Diseases

6. Department of Respiratory Medicine, Beijing Huimin Hospital, Beijing

7. Department of Respiratory Medicine, Rizhao Chinese Medical Hospital Affiliated to Shandong Chinese Medical University, RiZhao City, Shandong Province

For peer review only

8. Department of Respiratory Medicine, Linzi District People's Hospital, Zibo City, Shandong Province

9. Department of Respiratory Medicine, Beijing Luhe Hospital Affiliated to Capital Medical University, Beijing

10. Occupational Medicine and Toxicology Department, Beijing Chao-Yang Hospital, Capital Medical University, Beijing

11. Department of Pulmonary and Critical Care Medicine, Weifang NO.2 People's Hospital, Weifang City, Shandong Province

12. Department of Respiratory Medicine, Qilu Hospital Of Shandong University (Qindao), Qingdao City, Shandong Province

13. Department of Respiratory Medicine, The 2nd Hospital of Beijing Corps, Chinese Armed Police Forces, Beijing

14. Department of Infectious Disease, Yantai Yuhuangding Hospital, Yantai City, Shandong Province

15. Influenza Division, National Center for Immunization and Respiratory Diseases, Centers for Disease Control and Prevention-Atlanta, Georgia, USA

16. Department of Pulmonary Medicine; Capital Medical University, Beijing;

Corresponding Author: Dr. Bin Cao, MD. Center for Respiratory Diseases，Department of Pulmonary and Critical Care Medicine，China-Japan Friendship Hospital；National Clinical Research Center of Respiratory Diseases；Capital Medical University. Caobin_ben@vip.163.com; or Dr. Chen Wang at cyh-birm@263.net

For peer review only - http://bmjopen.bmj.com/site/about/guidelines.xhtml

Abstract

Objectives: To describe the clinical characteristics and management of patients hospitalized with CAP in China.

Design: This was a multicenter, retrospective, observational study.

Setting: 13 teaching hospitals in northern, central and southern China from 1 January 2014 to 31 December 2014

Participants: Information on hospitalized patients aged ≥14 years with radiographically-confirmed pneumonia with illness onset in the community was collected using standard case report forms.

Primary and secondary outcome measures: Resource use for CAP management.

Results: Of 14,793 patients screened, 6,056 with radiographically-confirmed CAP were included in the final analysis. Low mortality risk patients with a CURB-65 score 0-1 and PSI risk class Ⅰ-Ⅱ accounted for 73.2% (4434/5812) and 54.8% (2034/3710) of CAP patients respectively. 21.8% (1157/6056) patients had already achieved clinical stability on admission. 29.2% (1132/3880) patients without pseudomonal infection risk factors received antimicrobial over-treatment regimens. The median length of stay in hospital was 10 days. The median duration between clinical stability to discharge was 5.0 days with 30-day mortality of 4.2%.

Conclusions: These data demonstrated overuse of health resources in CAP management, indicating that there is the potential for improvement and substantial savings to health-care systems in China.

For peer review only - http://bmjopen.bmj.com/site/about/guidelines.xhtml

Strengths and limitations of this study

- This is the largest multi-center study to investigate demographic characteristics, severity and microbiological testing, empirical antimicrobial treatment, duration of hospitalization and 30-day mortality among adults and adolescents hospitalized with CAP in mainland China, including adolescents and adults of all ages admitted to general hospital wards or ICUs from the participating centers， patients who were critically ill, aged >90 years, and/or immunosuppressed.
- The participating hospital sites are teaching hospitals in seven cities in three provinces, and may not be representative of CAP in smaller, rural hospitals.
- The majority of patientsare adult CAP patients, our findings do not apply to children hospitalized with CAP.

For peer review only

Background

Community acquired pneumonia (CAP) is one of the most common infectious syndromes and is a leading cause of death worldwide [1-2]. In Europe, the reported rate of CAP ranges from 1·6 to 9 cases per 1,000 in the general adult population per year [3-5]. Despite advances in medical technology and global economic development, CAP-associated mortality remains high (e.g., 20.9/100,000 in the United States and 12.7/100,000 in Canada) [2,6]. Patients hospitalized in intensive care units for CAP have mortality in excess of 20% for immunocompetent patients and closer to 30% for those immunocompromised [7]. In Japan and Korea, the 30-day mortality of patients hospitalized with CAP is about 4-6% [8-9].

Although mainland China has nearly 19% of the world's population, there are limited data on CAP management and disease burden in China during the last ten years. According to a household interview survey published in the China Health and Family Planning Statistical Yearbook (2013), the two-week prevalence of pneumonia in China was estimated to be 11/1,000, and the direct cost due to bacterial pneumonia was about 320 million RMB (approximately $46.4 million)[10] . In 2015, CAP-China, a multicenter clinical network, was founded with the support of National Key Technology Support Program from Ministry of Science and Technology (2015BAI12B11) to provide data on CAP for clinical researchers and healthcare policy makers in China.

For peer review only - http://bmjopen.bmj.com/site/about/guidelines.xhtml

A multicenter retrospective study of all hospitalized CAP patients from 13 centers in northern, central and southern China among CAP-China members was implemented in 2015 (Clinicaltrial Registration No. NCT02489578). To our knowledge, this is the largest multi-center study to investigate demographic characteristics, severity and microbiological testing, empirical antimicrobial treatment, duration of hospitalization and 30-day mortality among adults and adolescents hospitalized with CAP in mainland China.

Methods

Study Design and Population

Data were collected from 13 hospitals in Northern (Beijing), Central (Yantai, Qindao, Weifang, Zibo, Rizhao cities in Shandong Province) and Southern (Kunming City in Yunan Province) China. A listing of participating centers can be found in Appendix 1. All patients admitted to the 13 hospitals during 1 January 2014 through 31 December 2014 with the relevant disease codes of pneumonia or pulmonary infection in the World Health Organization International Classification of Diseases 10th revision (ICD-10, Appendix 2) were eligible. Data on all eligible patients identified in screening were retrieved from the Hospital Information System (HIS) in each center. Trained physicians reviewed the medical case history and collected data on 786 variables for each patient. Chest radiographs and computerized tomography (CT) scans for each patient were reviewed by pulmonary physicians and radiologists in each center. Two-leveled review process was performed for data collection and entry. The CAP case definition includes (1) illness onset in the community; (2) chest

For peer review only - http://bmjopen.bmj.com/site/about/guidelines.xhtml

radiograph or CT scan showing infiltration or interstitial changes, with or without pleural effusion; (3) any one of pneumonia clinical manifestations: (a) recent cough, sputum or aggravation of respiratory symptoms, the emergence of purulent sputum, with or without chest pain; (b) fever (temperature ≥37.3°C) or hypothermia (temperature <36 °C); (c) signs of pulmonary consolidation and (or) moist crackles; or (d) WBC $>10\times10^9$/L, or $<4\times10^9$/L, with or without neutrophil predominance. Patients were excluded if (1) age <14 years; (2) pneumonia onset ≥48 hours after admission; (3) lung infiltrate or interstitial changes which were interpreted as lung cancer, pulmonary tuberculosis, non-infectious interstitial lung diseases, pulmonary edema, atelectasis, pulmonary embolism, pulmonary eosinophil infiltration, pulmonary vasculitis; (4) HIV positive; (5) re-admission within 72 hours after discharge.

The study design was approved by the Ethics committee of China-Japan Friendship Hospital (No. 2015-86). Given the retrospective nature of the study, the Ethics committee determined that informed consent was not necessary.

Quality control of the study

Key investigators, including clinicians, statisticians, microbiologists and radiologists worked together to draft the protocol and created a single formatted case report form (CRF) that was utilized by all centers. Before study initiation, all investigators from the thirteen centers received training on the protocol, screening process, definition of underlying diseases and formatted CRF（Appendix file 3). After data were collected, the CRF was reviewed by a trained researcher to ensure its completeness and data

For peer review only - http://bmjopen.bmj.com/site/about/guidelines.xhtml

quality. A second review was performed independently by a trained team of physicians in each center before being entering in duplicate into a computerized database.

Data Collection:

A total of 786 variables were included in the formatted CRF, including:

(1) Demographic data: age, gender, ID number, source of admission, types of medical insurance;

(2) Underlying diseases: chronic lung, heart, renal and liver diseases, diabetes, solid organ cancers, immunocompromised status, such as leukemia and lymphoma, chemotherapy or radiation within six months, bone marrow and solid organ transplantation, splenectomy. Definition of underlying diseases is listed in Appendix file 4.

(3) Factors for acquisition or prevention of CAP: pregnancy, postpartum within six months, current smoking history, excessive drinking, exposure to day care center children, bed-ridden longer than two months, chronic receipt of corticosteroids (dosage equivalent prednisolone ≥10mg/d for more than 30 days), statin use, *S. pneumoniae* or Influenza vaccination within one year.

(4) Clinical manifestations, clinical signs: recorded on the day of admission, on the 4th hospital day, change of antibiotics within 14 days of admission, and the day of discharge or death. Laboratory and radiological findings were also recorded if such tests were repeated by attending physicians. Pneumonia disease severity scores (PSI /CURB-65) were also recorded.

For peer review only - http://bmjopen.bmj.com/site/about/guidelines.xhtml

For peer review only

(5) Microbiological examination: Gram stain and culture of sputum within 48 hours, blood culture within 48 hours, urinary antigen testing, BALF and pleural fluid culture within one week after admission, serum antibody (including IgM and IgG) for atypical pathogens (*Mycoplasma pneumoniae, Chlamydia pneumoniae, Legionella pneumophila*) and respiratory virus, real-time PCR testing for atypical pathogens and respiratory viruses.

(6) Antimicrobial treatment before admission and change of antimicrobials during hospitalization. Use of corticosteroids, vasopressors, mechanical ventilation, Continuous renal replacement therapy (CRRT) and extracorporeal membrane oxygenation (ECMO) were also recorded.

(7) Clinical stability was defined as satisfying all of the following: temperature ≤37.8 °C more than 24 hours without use of antipyretic medications; resting heart rate ≤100 beats/min; respiratory rate ≤24 breaths/ minute; systolic blood pressure ≥90mmHg; SpO2 ≥ 90% on room air; ability to maintain oral intake; normal mental status[12].

(8) Over-treatment was defined as: (i) use of antipseudomonal β-lactams or β-lactams+ fluoquinolones in patients aged <65 years without risk factors for pseudomonal infection; (ii) use of (antipseudomal or not) β-lactams+ fluoquinolones in patients aged≥65 years without risk factors for pseudomonal infection and not in an ICU[13].

(9) Risk factors for pseudomonal infection was defined as chronic airway disease (bronchiectasis and COPD), immunocompromised status and at least one risk factor for HCAP as defined by the 2008 IDSA/ATS adult CAP guidelines. [11,13-16].

For peer review only - http://bmjopen.bmj.com/site/about/guidelines.xhtml

(10) Empirical antimicrobial regimens recommended by Chinese CAP guidelines were showed in Appendix 8.

Microbiology testing

The conditions that a pathogen was defined as the definite or probable etiology based on were showed in Appendix 6.

Statistical analysis

No formal sample size calculations were performed because of the retrospective descriptive study design. All data were analyzed by descriptive statistics with SPSS19. Measurement data were tested for normality by Kolmogorov-Smirnov. Measurement data of normal distribution was reported as mean ± standard deviation. Measurement data of non-normal distribution was reported as median. The $\chi 2$ test statistics were used for 30-day mortality subgroup analysis. A P-value of <0.05 was considered statistically significant.

Results

Screening Process

A total of 14,793 patients were screened to meet the inclusion and exclusion criteria for CAP and 6,056 patients were included in the final analysis (Appendix Figure 1).

Epidemiological characteristics

The proportions of male and female patients were similar. The median age was 65 years, range 14-103 years. Prevalent co-morbidities included hypertension (35.3%), coronary heart disease (19.8%), diabetes (15.8%), cerebrovascular diseases (15.2%) and COPD（13.7%）. 15.4% of CAP patients had at least one health care associated

For peer review only - http://bmjopen.bmj.com/site/about/guidelines.xhtml

pneumonia (HCAP) risk factor (according to IDSA/ATS HAP/HCAP guideline published in 2007 [7]). 45.6% patients received antibiotics before admission.

A substantial proportion of admitted patients had relatively mild disease as indicated by the following: i) CURB-65 score [17] 0-1 accounted for 76.3%, ii) PSI risk class [18]I~II accounted for 54.8%; iii) Shorr Score [19] 0~1 accounts for 98.7%; and iv) Aliberti Score low risk [20] group in 88.9%; v) only 12.1% (274/2260) patients had procalcitonin (PCT) more than 2 ng/ml; vi) as many as 65.2% (3854/5915) patients had normal peripheral leukocyte counts (4,000-10,000/ul). Most importantly, 21.8% patients had met criteria for clinical stability at hospital admission [12]. (Table 1-2)

Clinical and radiological features

Clinical and radiological features on admission are shown in Table 2. Cough, sputum, shortness of breath and fever were the most common. 64.3% patients had multi-lobar infiltrates and 20.8% of patients had pleural effusion.

Microbiological testing

75.9% patients had some type of microbiologic testing. 63.7% of patients had a sputum culture obtained within 48 hours of admission, although only 20.7% of patients were able to produce a sputum culture of acceptable quality. The proportion of patients with blood culture, BALF culture, and pleural effusion culture were 10.7%, 9.3% and 2·0% respectively. Only 0.1% of patients had a urinary antigen test sent to evaluate for *Legionella pneumophila*, and 2.6% had urinary antigen testing for *Streptococcus pneumoniae*. (Table 3)

Of all patients, serological testing for antibodies to *Mycoplasma pneumoniae* was only

For peer review only - http://bmjopen.bmj.com/site/about/guidelines.xhtml

performed on a single serum specimen for IgM (31%) and IgG antibodies (13.6%). Similarly, serological testing on a single serum specimen was done for *Chlamydia pneumoniae* IgM antibody in 22.2% of patients and for IgM antibodies to *Legionella pneumophila* and respiratory viruses in 11%. No convalescent serum specimens were collected for serological testing for any pathogens, limiting interpretation of serology results for a single serum specimen.

A definite or probable pathogen was identified only in 12.9% of patients (782/6,056): only bacteria in 86.7% (678/782), only atypical pathogens in 0.9% (7/782), only viruses in 8.6% (67/782), bacteria and viruses in 3.3% (26/782), viruses and atypical pathogens in 0.6% (5/782). The most common five pathogens identified were *Pseudomonas aeruginosa* 26.9% (210/782), *Klebsiella pneumoniae* 17.4% (136/782), *Escherichia. coli* 8.8% (69/782), *Acinetobacter* 8.4% (66/782) and influenza A virus 7.2% (56/782). (Appendix 6)

Empiric antimicrobial regimens

β-lactams (received by 72.4% of patients) and fluoquinolones (received by 42.2%) were the most common classes of antibiotics that were administered empirically. In patients without pseudomonal infection risk factors, 24.4% (471/1928) patients aged <65 years received empiric antibiotic regimens including antipseudomonal β-lactams, and 15.9% (306/1928) patients aged <65 years received β-lactams + fluoquinolones; 20.8% (355/1708) patients aged ≥65 years and not in ICU received β-lactams (antipseudomonal or not) + fluoquinolones combined regimens. The total percentage of patients who received over-treatment with empiric antibiotics was 29.2%

For peer review only - http://bmjopen.bmj.com/site/about/guidelines.xhtml

(1132/3880). (Table 4)

Clinical outcomes

Clinical outcomes are shown in Table 5. Overall, 6.5% of patients were admitted to an ICU, and 2.8% required invasive mechanical ventilation. Vasopressors were administered to 3.5% of patients, and 27.5% received corticosteroids during the hospitalization. The 30-day mortality was 4.2%. The median duration of hospitalization was 10 days. The median duration from admission to clinical stability was 4 days, and from clinical stability to discharge was 5 days. The median duration of ICU hospitalization was 8 days. The top five causes of death were severe pneumonia/multi-organ dysfunction syndrome (MODS) 66.9% (172/257), cardiac failure 3.1% (8/257), stroke 1.9% (5/257), acute myocardial infarction 1.9% (5/257), and gastrointestinal hemorrhage 1.9% (5/257).

Appendix 7 shows the results of sub-group analysis of 30-day mortality. Fatality increased with age and there was a jump up at 16.0% among those aged ≥90 years. Mortality was similar between male and female patients (4.9% vs 3.5%). Mortality in patients was >10% in patients with organ/bone marrow transplantation, immunosuppressive therapy, long-term oral corticosteroids use, chemotherapy/radiology within 6 months and splenectomy. Mortality in patients admitted to an ICU was 25.3%.

Discussion

This study represents the largest, multicenter, retrospective cohort study on the etiologies and outcomes in adolescents and adults with CAP in China. In this study,

For peer review only - http://bmjopen.bmj.com/site/about/guidelines.xhtml

we found that admission of patients with low mortality risk, overuse of antibiotics and unnecessary serological testing for *Mycoplasma pneumoniae*, *Chlamydia pneumoniae, Legionella pneumophila* and respiratory viruses, were the main challenges of CAP management.

We identified four major categories of overuse of health care resources in CAP management in China:

(1) A large number of low-risk patients were admitted to the hospitals. Guidelines for CAP management in China and the U.S. recommend that decisions for hospitalization should be based on illness severity [11,13]. It was estimated that over $8 billion dollars are spent in CAP treatment every year in the U.S, and the cost for inpatient CAP management is 25-30 times more than for outpatient CAP management [22-24]. Therefore, admission of low mortality risk CAP patients results in major unnecessary cost expenditures. Moreover, outpatients usually return to their baseline activity levels much sooner than inpatients, and enjoyed a higher quality of life [25-26]. Finally, hospitalization is associated with the risk of nosocomial infections, potentially caused by high virulent and multidrug-resistant organisms [27]. Admission of low-risk CAP patients was also observed in a recent large U.S. study [28], so it may not be unique to China.

(2) Length of stay in hospital was unnecessarily long. CAP guidelines recommended that patients should be discharged as soon as they achieve clinical stability and have no other active medical problems. Keeping patients in hospital and observing them while receiving oral antibiotic therapy, or waiting for normalization of all clinical

For peer review only - http://bmjopen.bmj.com/site/about/guidelines.xhtml

parameters are not indicated and are associated with increased costs and potentially with in-hospital adverse events [27,29-30]. We observed that CAP patients were discharged a median of 5 days after achieving clinical stability, and 22% met clinical stability criteria at admission. Given the median LOS of 10 days for all CAP patients, discharging CAP patients once they achieved clinical stability would lead to cost-savings of approximately half of the total hospitalization expenses.

(3) 29.1% patients without risk factors for *Pseudomonas* infection received over-treatment with empiric antimicrobial regimens. Antipseudomonal β-lactams (17.0%) or β-lactams + quinolones (12.1%) were the most common empiric regimens for over-treatment. This may be due to overestimation of illness severity, clinician unfamiliarity with CAP guidelines, or lack of microbiologic diagnostic testing. Moreover, we found quinolones use in more than 40% of CAP patients. The U.S. Food and Drug Administration (FDA) has released warnings of potential adverse effects of fluoroquinolones, such as Q-T prolongation, tendon injury, psychiatric disorder, etc [31-33]. As second-line anti-tuberculosis drugs, fluoroquinolones can also affect the diagnosis of tuberculosis and induce drug-resistance [34-35].

(4) Unnecessary serological testing was performed. We observed that many patients had an acute serum specimen collected for IgG serology testing for atypical bacteria and respiratory viruses without a convalescent serum specimen obtained for paired serological testing. Furthermore, many patients had testing for IgM antibodies for a variety of respiratory pathogens, but elevation of IgM antibodies with a low-normal IgG titer is uncommon during acute illness [36-38].Similarly, although a low IgM

For peer review only - http://bmjopen.bmj.com/site/about/guidelines.xhtml

antibody level with a high IgG titer would be suggestive of past infection, the performance characteristics of these assays may not be reliable. A follow-up convalescent serum specimen to document changes in IgG and IgM antibody levels is generally required for diagnosis [39-40] . Thus, the value of antibody testing on a single acute serum specimen to determine the etiology of CAP is questionable. The costs of more frequent use of PCR testing on lower respiratory specimens may be partially offset by not performing serological testing in CAP patients.

Although we identified substantial over-use of health-care resources, the outcome of patients hospitalized with CAP in China was not ideal. Although the 30-day mortality was low (4.2%), this should be interpreted in the context that approximately 70% had mild CAP as indicated by pneumonia scoring indices. Mortality for CAP patients with a CURB-65 score of ≥2 (15.8%) or PSI risk ≥Ⅲ risk class (9.1%) were higher than what has been reported in developed countries, especially in critically ill patients with a CURB-65 score of 3-5 and PSI risk Ⅳ-Ⅴ class [41-42].

The strengths of this study, in contrast to some past epidemiological investigations [21], included data on bacterial isolates obtained in current clinical practice, microbiologic testing ordered, and antimicrobials administered, according to Chinese standards-of-care, and the study population included adolescents and adults of all ages admitted to general hospital wards or ICUs from the participating centers to reduce selection bias. We also included patients who were critically ill, aged >90 years, with risk factors for HCAP, and/or immunosuppressed (e.g., cancer, chronic corticosteroid

For peer review only - http://bmjopen.bmj.com/site/about/guidelines.xhtml

use or receipt of other immunosuppressive agents, splenectomy, etc., excluding HIV infection).

This study had several limitations. First, given the retrospective study design, it is possible that selection bias was present and the study population may not have been representative of all CAP patients admitted to the 13 participating sites. Secondly, the participating hospital sites were teaching hospitals in seven cities in three provinces, and were not selected to be representative of CAP hospital management in China, especially in smaller, rural hospitals. Third, this study reports on CAP management during 2014; analysis of multiple years of data can allow assessment of changes in CAP management. Fourth, there was no standardization of CAP admission or discharge criteria and this may have varied among hospitals. We realize that admission of clinically stable patients or those in low-risk mortality groups, and prolonged hospitalization despite achieving clinical stability may be based upon social rather than clinical factors, such as lack of available family support, older age, mental illness and drug abuse, etc [30,43]. Fifth, the use of sputum to detect bacterial etiologies of CAP may represented identification of colonization rather than infection. Sixth, 45.6% of CAP patients received antibiotics before hospital admission and before specimen collection, and this likely reduced the detection of some bacterial infections, such as *Streptococcus pneumoniae*. Therefore, the bacterial pathogens identified in this study may not be representative of all bacterial causes of CAP in the source patient populations for this study. Finally, while we included adolescents, the majority

For peer review only - http://bmjopen.bmj.com/site/about/guidelines.xhtml

of patients were adult CAP patients, and our findings do not apply to children hospitalized with CAP.

In conclusion, we characterized adolescents and adults hospitalized for CAP in China and identified several problems suggesting the over-use of healthcare resources in CAP management. This suggests that education and training of clinicians on current CAP guidelines in China are needed to improve clinical management and could also result in substantial cost-saving in healthcare expenditures for CAP patients. The multi-center hospital network can serve as a platform for conducting intervention studies for hospitalized CAP patients in the future, utilizing the baseline data from this observational study.

Acknowledgments

We thank Dr. Jay Purdy (Senior Director, Anti-infectives, Pfizer Inc, 500 Arcola Rd, F3203, Collegeville, PA 19426), Francesco Blasi (Department of Pathophysiology and Transplantation, Università degli Studi di Milano, Cardio-thoracic Unit and Cystic Fibrosis Adult Center Fondazione IRCCS Cà Granda Ospedale Maggiore Policlinico Milano, Milan, Italy) , and Richard G. Wunderink (Division of Pulmonary and Critical Care Medicine, Department of Medicine, Northwestern University Feinberg School of Medicine, Chicago, IL, USA) for his valuable comments on the manuscript.

Contributors Study design: Liang Chen, Feizhou, Hui Li, Chen Wang, Bin Cao. Data collection: Liang Chen, Fei Zhou, Hui Li, Xiqian Xing, Xiudi Han,Yimin Wang, Chunxiao Zhang, Lijun Suo, Jinxiang Wang, Guohua Yu, Guangqiang Wang, Xuexin

For peer review only - http://bmjopen.bmj.com/site/about/guidelines.xhtml

Yao, Hongxia Yu, Lei Wang, Meng Liu, Chunxue Xue, Bo Liu, Xiaoli Zhu, Yanli Li, Ying Xiao, Xiaojing Cui, Lijuan Li. Statistical analysis**:** Liang Chen, Fei Zhou, Hui Li, Xiudi Han. Writing: Liang Chen, Bin Cao, Timothy M. Uyeki. All authorstake full responsibility for the study design, data analysis and interpretation, and preparation of the manuscript. All authors approved the final draft manuscript.

Funding This work was funded by National Science Grant for Distinguished Young Scholars (81425001/H0104);National Key Technology Support Program from Ministry of Science and Technology (2015BAI12B11);The Beijing Science and Technology Project (D151100002115004)

Disclaimer The views expressed are those of the authors and do not necessarily reflect the official policy of the Centers for Disease Control and Prevention.

Competing interests None declared.

Data sharing statement No additional data available

References

1. Miniño AM, Heron MP, Smith BL. Deaths: preliminary data for 2004. Natl Vital Stat Rep. 2006 Jun 28;54 (19):1-49.

2. File TM Jr, Marrie TJ. Burden of community-acquired pneumonia in North American adults. Postgrad Med. 2010 Mar;122(2):130-41. doi: 10.3810/pgm.2010.03.2130.

3. Almirall J, Bolíbar I, Vidal J, et al. Epidemiology of community-acquired pneumonia in adults: a population-based study. Eur Respir J. 2000 Apr;15 (4):757-63.

4. Jokinen C, Heiskanen L, Juvonen H, et al. Incidence of community-acquired

For peer review only - http://bmjopen.bmj.com/site/about/guidelines.xhtml

pneumonia in the population of four municipalities in eastern Finland. Am J Epidemiol. 1993 May 1; 137(9): 977-88.

5. Woodhead MA, Macfarlane JT, McCracken JS, et al. Prospective study of the aetiology and outcome of pneumonia in the community. Lancet. 1987 Mar 21; 1(8534):671-4.

6. Miniño AM, Heron MP, Smith BL. Deaths: preliminary data for 2004. Natl Vital Stat Rep. 2006; 54: 19

7. Health, United States, 2012: with special features on emergency care. Hyattsville, MD: National Center for Health Statistics, 2013: 297-299.

8. Uematsu H. Development of a risk-adjusted in-hospital mortality prediction model for community-acquired pneumonia: a retrospective analysis using a Japanese administrative database. BMC Pulm Med. 2014, 14: 203-210

9. Kim HI. Mortality of community-acquired pneumonia in Korea: assessed with the pneumonia severity index and the CURB-65 score. J Korean Med Sci. 2013 Sep; 28 (9):1276-82.

10. National Health and Family Planning Commission of the People's Republic of China. China health and family planning statistics yearbook 2013. (Available at: http://www.nhfpc.gov.cn/htmlfiles/zwgkzt/ptjnj/year2013/index2013.html)

11. American Thoracic Society. Infectious Diseases Society of America. Guidelines for the management of adults with hospital-acquired, ventilator-associated, and healthcare-associated pneumonia.Am J Respir Crit Care Med. 2005 Feb 15; 171(4):388-416

For peer review only

For peer review only - http://bmjopen.bmj.com/site/about/guidelines.xhtml

12. Ramirez JA, Vargas S, Ritter GW, et al. Early switch from intravenous to oral antibiotics and early hospital discharge: a prospective observational study of 200 consecutive patients with community-acquired pneumonia. Arch Intern Med 1999;159: 2449–54.

13. Guidelines for the diagnosis and treatment of adult community acquired pneumonia in China (2016 Edition)]. Zhonghua Jie He He Hu Xi Za Zhi. 2016 Apr 12; 39 (4): 241-2

14. Rañó A, Agustí C, Benito N,et al. Prognostic factors of non-HIV immunocompromised patients with pulmonary infiltrates.Chest. 2002 Jul;122(1): 253-61

15. Al-Anazi KA, Al-Jasser AM.Infections Caused by Acinetobacter baumannii in Recipients of Hematopoietic Stem Cell Transplantation.Front Oncol. 2014 Jul 14;4:186-95

16. Rañó A, Agustí C, Benito N,et al. Prognostic factors of non-HIV immunocompromised patients with pulmonary infiltrates. Chest. 2002 Jul;122 (1):253-61

17. Lim WS, van der Eerden MM, Laing R, et al. Defining community acquired pneumonia severity on presentation to hospital: an international derivation and validation study.Thorax. 2003 May;58 (5):377-82.

18. Fine MJ, Auble TE, Yealy DM, et al. A prediction rule to identify low-risk patients with community-acquired pneumonia. N Engl J Med 1997;336:243–50.

19. Shorr AF, Zilberberg MD, Reichley R, et al. Validation of a clinical score for

For peer review only - http://bmjopen.bmj.com/site/about/guidelines.xhtml

assessing the risk of resistant pathogens in patients with pneumonia presenting to the emergency department. Clin Infect Dis 2012;54:193–8

20. Aliberti S, Di Pasquale M, Zanaboni AM, et al. Stratifying risk factors for multidrug-resistant pathogens in hospitalized patients coming from the community with pneumonia. Clin Infect Dis. 2012;54:470–8

21. Liu YN, Chen MJ, Zhao TM, et al. A multicentre study on the pathogenic agents in 665 adult patients with community-acquired pneumonia in cities of China. Zhonghua Jie He He Hu Xi Za Zhi. 2006 Jan; 29 (1):3-8.

22. Broulette J, Yu H, Pyenson B, et al. The Incidence Rate and Economic Burden of Community-Acquired Pneumonia in a Working-Age Population。Am Health Drug Benefits. 2013 Sep; 6 (8):494-503.

23. Niederman MS, McCombs JS, Unger AN, et al. The cost of treating community-acquired pneumonia. Clin Ther. 1998 Jul-Aug; 20 (4):820-37.

24. Rozenbaum MH, Mangen MJ, Huijts SM, et al. Incidence, direct costs and duration of hospitalization of patients hospitalized with community acquired pneumonia: A nationwide retrospective claims database analysis. Vaccine. 2015 Jun 22;33 (28):3193-9. doi: 10.1016/j.vaccine.2015.05.001.

25. Coley CM, Li YH, Medsger AR, et al. Preferences for home vs hospital care among low-risk patients with community-acquired pneumonia. Arch Intern Med. 1996 Jul 22;156 (14):1565-71

26. Carratalà J, Fernández-Sabé N, Ortega L, et al. Outpatient care compared with hospitalization for community-acquired pneumonia:

For peer review only - http://bmjopen.bmj.com/site/about/guidelines.xhtml

a randomized trial inlow-risk patients.Ann Intern Med. 2005 Feb 1;142 (3):165-72.

27. Rhew DC, Riedinger MS, Sandhu M, et al. A prospective, multicenter study of a pneumonia practice guideline. Chest 1998;114:115–9.

28. Jain S, Self WH, Wunderink RG, et al. Community-Acquired Pneumonia Requiring Hospitalization among U.S. Adults. N Engl J Med. 2015 Jul 30;373 (5):415-27. doi: 10.1056/NEJMoa1500245. Epub 2015 Jul 14.

29. Ramirez JA, Vargas S, Ritter GW, et al. Early switch from intravenous to oral antibiotics and early hospital discharge: a prospective observational study of 200 consecutive patients with community-acquired pneumonia. Arch Intern Med 1999;159:2449–54.

30. Halm EA, Switzer GE, Mittman BS, et al. What factors influence physicians' decisions to switch from intravenous to oral antibiotics for community-acquired pneumonia? J Gen Intern Med 2001;16:599–605.

31. Aschenbrenner DS. The FDA Revises Boxed Warning For Fluoroquinolones-Again. Am J Nurs. 2016 Sep;116 (9):22-3. doi: 10.1097/01.NAJ.0000494691.55746.90.

32. Tillotson GS. FDA and the safe and appropriate antibiotic use of fluoroquinolones. Lancet Infect Dis. 2016 Mar;16 (3):e11-2. doi: 10.1016/S1473-3099(16)00051-7.

33. Lu ZK, Yuan J, Li M, et al. Cardiac risks associated with antibiotics: azithromycin and levofloxacin. Expert Opin Drug Saf. 2015

34.Rush B, Wormsbecker A, Stenstrom R, et al. Moxifloxacin Use and Its Association on the Diagnosis of Pulmonary Tuberculosis in An Inner City Emergency Department.

For peer review only - http://bmjopen.bmj.com/site/about/guidelines.xhtml

J Emerg Med. 2016 Mar;50 (3):371-5. doi: 10.1016/j.jemermed.2015.07.044. Epub 2015 Sep 26.

35. Mirza IA, Khan FA, Khan KA, et al. Extensively and pre-extensively drug resistant tuberculosis in clinical isolates of multi-drug resistant tuberculosis using classical second line (levofloxacin and amikacin). J Coll Physicians Surg Pak. 2015 May;25(5):337-41.

36.Qu J, Gu L, Wu J, et al. Accuracy of IgM antibody testing, FQ-PCR and culture in laboratory diagnosis of acute infection by Mycoplasma pneumoniae in adults and adolescents with community-acquired pneumonia. BMC Infect Dis. 2013 Apr 11;13:172-7.

37. Angrup A, Chaudhry R, Sharma S, et al. Application of real-time quantitative polymerase chain reaction assay to detect Legionella pneumophila in patients of community-acquired pneumonia in a tertiary care hospital. Indian J Med Microbiol. 2016 Oct-Dec;34(4):539-543. doi: 10.4103/0255-0857.195353.

38. Bartlett JG. Diagnostic test for etiologic agents of community-acquired pneumonia. Infect Dis Clin North Am. 2004 Dec;18(4):809-27.

39. Sayan M, Kilinç O, Yüce A, et al. Seropositivity against atypical pneumonia agents demonstrated in patients with community-acquiredpneumonia. Mikrobiyol Bul. 2003 Oct;37(4):247-53.

40. Salo P, Leinonen M. Microbiological investigations. Semin Respir Infect. 1999 Jun;14(2):128-34.

41. Arnold FW, Wiemken TL, Peyrani P, et al. Mortality differences among

For peer review only

hospitalized patients with community-acquired pneumonia in three world regions: results from the Community-Acquired Pneumonia Organization (CAPO) International Cohort Study. Respir Med. 2013 Jul;107(7):1101-11.

42. Blasi F, Garau J, Medina J, et al. Current management of patients hospitalized with community-acquired pneumonia across Europe: outcomes from REACH. Respir Res. 2013 Apr 15;14:44. doi: 10.1186/1465-9921-14-44.

43. Goss CH, Rubenfeld GD, Park DR, Sherbin VL, Goodman MS, Root RK. Cost and incidence of social comorbidities in low-risk patients with community-acquired pneumonia admitted to a public hospital. Chest 2003; 124:2148–55.

For peer review only - http://bmjopen.bmj.com/site/about/guidelines.xhtml

Table 1: Demographic characteristics and underlying diseases

Items	Cases (%)
Male	3245 (53.6)
Age (years,median, IQR)	65 (53-78)
14~64	2925 (48.3)
65~74	1134 (18.7)
75~89	1810 (29.9)
≥90	187 (3.1)
Source of admission（n=6051）	
From Out-patient Department	4355 (72.0)
From Emergency Room	1640 (27.1)
Transfer from other hospital	56 (0.9)
Days from illness onset to admission (n=6054, median, IQR)	6·0 (11.0)
Patients who received antibiotics before admission	2759 (45.6）
β-lactams	1031 (37.4)
Fluoquinolones	596 (21.6)
Macrolides	174 (6.3)
β-lactams+ fluoquinolones	459 (16.7)
β-lactams+ macrolides	216 (7.8)
Others	283 (10.3)
Systemic glucocorticosteroids use before admission	283 (4.7)
Underlying Diseases	
Hypertension	2136 (35.3)
Coronary Heart Disease	1202 (19.8)
Diabetes	955 (15.8)
Cerebrovascular Diseases	921 (15.2)
COPD	827 (13.7)
Bronchiectasis	652 (10.8)
Asthma	351 (5.8)
Malignant solid tumors	309 (5.10)
Chronic renal diseases	222 (3.8)
Congestive Heart Failure	211 (3.5)
Connective Tissue Diseases	171 (2.8)
Chronic Hepatic Diseases	96 (1.6)

For peer review only

Immunocompromised Status	
Long-term oral Corticosteroid use	100 (1.7)
Chemotherapy/Radiotherapy within 6 months	71 (1.2)
Hematological neoplasms	54 (0.9)
Immunosuppressive therapy	52 (0.9)
Organ/Bone Marrow Transplantation	13 (0.2)
Splenectomy	8 (0.1)
Smoking status	
Current smokers	1038 (17.1)
Ex-smokers	608 (10.0)
Alcoholism	424 (7.0)
Risk factors for aspiration	384 (6.3)
History of CAP within one year	391 (6.5)
History of vaccination	
Influenza vaccine within 1 year	12 (0.2)
Streptococcus pneumoniae vaccine within 5 years	8 (0.1)
Risk factors for HCAP according to IDSA/ATS criteria	933 (15.4)
Hospitalized in an acute care hospital for two or more days within 90 days	454 (7.5)
Received recent intravenous antibiotic therapy, chemotherapy, or wound care within the past 30 days	696 (11.5)
Attended a hospital or hemodialysis clinic	41 (0.7)
Residence in a nursing home or long-term care facility	19 (0.3)
CURB-65 score (n=5812)	
0	2185 (37.6)
1	2249 (38.7)
2	1036 (17.8)
3	288 (5.0)
4	52 (0.9)
5	2 (0.0)
PSI risk class (n=3710)	
[illegible]	1147 (30.9)
[illegible]	887 (23.9)
[illegible]	746 (20.1)
[illegible]	709 (11.7)

For peer review only

□	221 (6.0)
Shorr Score (n=5865)	
0	5106 (87.1)
1	685 (11.7)
2	71 (1.2)
3	3 (0.0)
4	0 (0.0)
Aliberti Score（n=6056)	
Low risk group	5386 (88.9)
High risk group	670 (11.1)
Clinical stability on admission (n=5311)	1157 (21.8)

COPD: chronic obstructive pulmonary disease; HCAP: healthcare associated pneumonia; IDSA/ATS: Infectious Diseases Society America/American Thoracic Society. PSI: pneumonia severity index. Clinical stability was defined as satisfying the following at the same time: temperature ≤37.8 °C more than 24 hours; heart rate ≤100 beats/min in resting state; breathing rate ≤24 breaths/minute; systolic blood pressure ≥90mmHg; SpO_2 ≥90% on room air; ability to maintain oral intake; normal mental status.

For peer review only

Table 2: Clinical and radiological features on admission

Items	Cases（%）
Fever (T≥38℃, n=6052)	2905 (48.0)
Hypothermia (T<36℃, n=6052)	51 (0.8)
Cough	5389 (89.0)
Sputum	4928 (81.4)
Shortness of breath	2191 (36.2)
Chest pain	732 (12.1)
Decrease of consciousness	308 (5.1)
Chest signs	
Moist rales	3033 (50.1)
Dry rales	1436 (23.7)
Edema of lower limbs	616 (10.2)
Cyanosis	565 (9.3)
SBP<90 mmHg	537 (8.9)
Radiology	
Infiltrate more than two lobes	3894 (64.3)
Plural effusion	1148 (20.8)
Cavitation	237 (3.9)
WBC (mm^{-3}, n=5915)	
>10,000	1692 (28.6)
<4,000	369 (6.2)
4,000~10,000	3854 (65.2)
BUN >7.0 $mmol \cdot L^{-1}$ (n=5819)	1222 (21.0)
PH <7.30 (n=3456)	90 (2.6)
PaO_2/FiO_2<300 mmHg (n=3453)	1348 (39.0)
PCT（$ng \cdot ml^{-1}$, n=2260)	
PCT≤0.25	1350 (59.7)
0.25<PCT<1	506 (22.4)
1≤PCT<2	130 (5.8)
PCT≥2	274 (12.1)

SBP: systolic blood pressure; WBC: white blood cell count; BUN: blood urea nitrogen; Scr: serum creatinine; PH: potential of hydrogen; PaO_2/FiO_2: arterial pressure of oxygen/fraction of inspiration oxygen; PCT: procalcitonin.

For peer review only

Table 3: Microbiological examination for CAP

**Items**	**Cases (%)**
Any Microbiological examination	4596 (75.9)
Microbiological examination for bacterial	4210 (69.5)
Microbiological examination for atypical etiology	2045 (33.8)
Microbiological examination for virus	2137 (34.2)
Bacterial or fungal Culture	
Qualified sputum culture*	1126 (18.6)
Blood culture **	645 (10.7)
BALF culture*[+]	565 (9.3)
Pleural effusion culture**	122 (2.0)
**Antibody-Based Assays on acute serum**	
Mycoplasma pneumoniae	IgM: 1878 (31.0)<br>IgG: 822 (13.6)
Chlamydia pneumoniae	IgM: 1342 (22.2)<br>IgG: 223 (3.7)
Legionella pneumoniae	IgM: 665 (11.0)<br>IgG: 230 (3.8)
Adenovirus	IgM: 665 (11.0)<br>IgG: 0 (0.0)
Respiratory syncytial virus	IgM: 664 (11.0)<br>IgG: 0 (0.0)
Influenza A virus	IgM: 664 (11.0)<br>IgG: 0 (0.0)
Influenza B virus	IgM: 661 (10.9)<br>IgG: 0 (0.0)
Parainfluenza virus	IgM: 664 (11.0)<br>IgG: 0 (0.0)
**Nucleic Acid-Based Molecular Diagnostics**	
From sputum	324(5.4)
Time Interval¶(days, median, IQR)	12.0 (15.5)
From BALF[+]	22 (0.4)

For peer review only

Time Interval¶(days, median, IQR)	15.0 (15.5)
Mycoplasma pneumoniae	337 (5.6)
Chlamydia spp	337 (5.6)
Legionella spp	337 (5.6)
Influenza A virus	337 (5.6)
Influenza B virus	337 (5.6)
Other respiratory virus#	337 (5.6)
**Antigen test**	
Streptococcus pneumoniae	159 (2.6)
Legionella spp	49 (0.8)
Influenza A virus	116 (1.9)
Influenza B virus	30 (0.5)

*： within 48hr after admission

**:within one week after admission

¶: days from illness onset to testings

# parainfluenza virus (PIV) types 1, 2, 3 and 4, rhinovirus (HRV), enterovirus (EV), coronovirus (hCoV) types 229E, NL63, OC43 and HKU1, parapneumovirus (hMPV), and adenovirus (AdV), bocavirus

$^{+}$**:** bronchoalveolar lavage fluid

For peer review only

Table 4: Empirical antimicrobial regimen for CAP patients (n=5942)*

Empirical antimicrobials (%)	Without risk factors for *P. seudomonas* infection (n=3880)				With risk factors for *P. seudomonas* infection (n=2062)
	age<65yr and not in ICU (n=1849)	age<65yr and in ICU (n=79)	age≥65yr and not in ICU (n=1708)	age≥65yr and in ICU (n=145)	
β-lactams (antipseudomonal)	183 (4.7)#	21 (0. 5)#	411 (10.6)	58 (1.5)	574 (27.8)
β-lactams	333 (8.6)	9 (0.2)	487 (12.6)	20 (0.5)	394 (19.1)
Fluoquinolones	509 (13.1)	10 (0.3)	275 (7.1)	6 (0.2)	288 (14.0)
Macrolides	20 (0.5)	0 (0.0)	17 (0.4)	0 (0.0)	15 (0.7)
β-lactams (antipseudomonal) + fluoquinolones	204 (5.3)#	13 (0.3)#	189 (4.9)#	30 (0.8)	256 (12.4)
β-lactams+ fluoquinolones	303 (7.8)#	3 (0.1)#	166 (4.3)#	9 (0.2)	191 (9.3)
β-lactams+ macrolides	161 (4.1)	2 (0.1)	64 (1.6)	2 (0. 1)	60 (2.9)
β-lactams (antipseudomonal) + macrolides	50 (1.3)#	0 (0.0%)#	45 (1.2%)	2 (0.·1%)	62 (3.0%)
Fluoquinolones + macrolides	24 (0.6)	0 (0.0)	11 (0.3)	0 (0.0)	7 (0.3)
Others	108 (2.8)	22 (0.6)	90 (2.3)	23 (0.6)	215 (10.4)

*: data on empirical antimicrobial regimens in 114 patients were missing.

#Overtreatment was defined as: 1) use of antipseudomonal β-lactams or β-lactams+ fluoquinolones in patients aged <65yr without risk factors for *P.seudomonas* infection; 2) use of (antipseudomal or not) β-lactams+ fluoquinolones in patients aged > 65yr without risk factors for *P. seudomonas* infection and not in ICU [13].

- Risk factors for *P. seudomonas* infection was defined as chronic airway disease (bronchiectasis or COPD), immunocompromised status and HCAP according to IDSA/ATS criteria [13].

For peer review only

Table 5: Supportive treatment and clinical outcomes of patients with CAP

**Items**	**Cases（%）**
ICU admission	391 (6.5)
Mechanical ventilation	
Non-invasive ventilation	304 (5.0)
Invasive ventilation in ICU	136 (2.2)
Invasive ventilation not in ICU	35 (0.6)
Vasopressor use	214 (3.5)
CRRT	18 (0.3)
ECMO	3 (0.0)
Systemic glucocorticosteroids use after diagnosis of CAP	1664 (27.5)
ICU patients who received systemic glucocorticoids	173 (10.4)
Patients on invasive mechanical ventilation who received systemic glucocorticoids	87 (5.2)
Patients on non-invasive mechanical ventilation who received systemic glucocorticoids	175 (10.5)
30-day mortality	257 (4.2)
Length of stay in Hospital (days, median, IQR)	10.0 (8.0-14.0)
Days between admission-clinical stability (median, n=5278, IQR)	4.0 (1.0-10.0)
Days between clinical stability-discharge (median, n=5311, IQR)	5.0 (1.0-9.0)
Length of stay in ICU (days, median, n=373,IQR)	8.0 (4.0-16.0)
Treatment failure within 14 days	459 (7.6)
Needs non-invasive ventilation	180 (3.0)
Needs invasive ventilation	161 (2.7)
Needs vasopressors	144 (2.4)
Death	168 (2.8)
Direct causes of death	
Severe pneumonia/MODS	172 (66.9)
Heart failure	8 (3.1)
Stroke	5 (1.9)
Acute myocardial infarction	5 (1.9)
Hemorrhage of digestive tract	5 (1.9)

For peer review only

Acute renal failure	3 (1.2)
Arhythmia	3 (1.2)
Accident aspiration	2 (0.8)
Others	54 (21.0)

ICU: intensive care unit; CRRT: continuous renal replacement therapy; ECMO: extracorporeal membrane oxygenation; MODS: multiple organ dysfunction syndrome; DIC: disseminated intravascular coagulation.

For peer review only

Appendix 1: Details of Participating centers

**Name of the hospital**	**Province, city**	**2nd and 3rd level hospital**	**Teaching Hospital**	**Beds**	**Staff of Clinical Microbioloy Lab**
Beijing Chao-Yang Hospital Affiliated to Capital Medical University	Beijing	3rd	Yes	1400	11
Beijing Jishuitan Hospital 4th Medical College of Peking University	Beijing	3rd	Yes	1500	10
Beijing Luhe Hospital Affiliated to Capital Medical University	Beijing	3rd	Yes	1042	5
Qingdao Municipal Hospital	ShanDong, Qingdao	3rd	Yes	1200	4
Qilu Hospital Of Shandong University(Qindao)	ShanDong, Jinan	3rd	Yes	1200	6
Beijing Huimin Hospital	Beijing	2nd	Yes	500	2
Linzi District People's Hospital	ShanDong, Zibo	2nd	Yes	1200	5
The 2nd Hospital of Beijing Corps, Chinese Armed Police Forces	Beijing	3rd	Yes	450	2
China-Japan Friendship Hospital	Beijing	3rd	Yes	1610	9
Yan'an Hospital Affiliated to Kunming Medical University	Kunming, Yan'an	3rd	Yes	1302	4

For peer review only

For peer review only - http://bmjopen.bmj.com/site/about/guidelines.xhtml

Yantai Yuhuangding Hospital	Shangdong, Yantai	3rd	Yes	3000	6
Rizhao Chinese Medical Hospital Affiliated to Shandong Chinese Medical University	Shangdong, Rizhao	3rd	Yes	1212	8
Weifang NO.2 People's Hospital	Shangdong, Weifang	3rd	Yes	1006	8

Definition of 2nd and 3rd level hospital in China:

The 2nd level hospital was defined as a hospital providing medical, prevention, health care and rehabilitation services to multiple communities (with a radius of population more than 100,000 peoples); the 3rd level hospital was defined as a hospital providing medical service to the whole country beyond cities and provinces, with comprehensive medical, teaching and research ability.

For peer review only - http://bmjopen.bmj.com/site/about/guidelines.xhtml

Appendix 2: ICD-10

Influenza with pneumonia, other influenza virus identified	J10.0
Influenza with pneumonia, virus not identified	J11.0
Virus pneumonia, not elsewhere classified	J12
Adenoviral pneumonia	J12.0
Respiratory syncytial virus pneumonia	J12.1
Parainfluenza virus pneumonia	J12.2
Other virus pneumonia	J12.8
Viral pneumonia,unspecified	J12.9
Pneumonia due to *Streptococcus pneumoniae*	J13
Pneumonia due to *Haemophilus influenzae*	J14
Bacterial pneumonia, not elsewhere classified	J15
Pneumonia due to *Klebsiella pneumoniae*	J15.0
Pneumonia due to *Pseudomonas spp.*	J15.1
Pneumonia due to *Staphylococcus*	J15.2
Pneumonia due to *Streptococcus spp*., group B	J15.3
Pneumonia due to other *streptococci*	J15.4
Pneumonia due to *Escherichia coli*	J15.5
Pneumonia due to other aerobic Gram-negative bacteria	J15.6
Pneumonia due to Mycoplasma pneumoniae	J15.7
Other bacterial pneumonia	J15.8

For peer review only

For peer review only - http://bmjopen.bmj.com/site/about/guidelines.xhtml

Bacterial pneumonia, unspecified	J15.9
Pneumonia due to other infectious organisms, not elsewhere classified	J16
Chlamydia pneumonia	J16.0
Pneumonia due to other specified infectious organisms	J16.8
Pneumonia due to other specified infectious organism	J16.8
Pneumonia in diseases classified elsewhere	J17*
Pneumonia in bacterial diseases classified elsewhere	J17.0*
Pneumonia in viral diseases classified elsewhere	J17.1*
Pneumonia in mycoses	J17.2*
Pneumonia in other diseases classified elsewhere	J17.8*
Pulmonary mycobacterial infection	A31.0
Pulmonary actinomycosis	A42.0
Pulmonary nocardiosis	A43.0
Legionnaires' disease	A48.1
Varicella pneumonia	B01.2+
Measles complicated by pneumonia	B05.1+
Cytomegaloviral pneumonitis	B25.0+
Pulmonary candidiasis	B37.1
Acute pulmonary coccidioidomycosis	B38.0
Acute pulmonary histoplasmosis capsulati	B39.0
Acute pulmonary blastomycosis	B40.0

For peer review only - http://bmjopen.bmj.com/site/about/guidelines.xhtml

Pulmonary paracoccidioidomycosis	B41.0
Pulmonary sporotrichosis	B42.0+
Invasive pulmonary aspergillosis	B44.0
Other pulmonary aspergillosis	B44.1
Pulmonary cryptococcosis	B45.0
Pulmonary mucormycosis	B46.0
Pneumonia, organism unspecified	J18
Bronchopneumonia, unspecified organism	J18.0
Lobar pneumonia, unspecified	J18.1
Hypostatic, pneumonia, unspecified	J18.2
Other pneumonia, organism unspecified	J18.8
Pneumonia, unspecified	J18.9

For peer review only

Appendix 3: Case Report Form Of Patients Hospitalized With CAP/HCAP

**Code：**	**R-**
**Name：**	**Gender： ○Male ○Female**
**Age： _____years old**	**Nationality： ○Han ○Others**
**Eight： _____cm**	**Weight： __ kg**
**ID Number：**	
**Date Of Admission： _____Y___M___D**	
**Case Number ：**	**ID Number:**
**Admission Form：**	**○Outpatience ○Emergency ○Transfers**
**Tel：**	**Cell Phone：**
**Provider Payments： ○Social Medical Insurance ○New Rural Cooperative Medical System ○ Medical Services At State Expense ○ Commercial Medical Insurance ○Self-paying ○Others**	

Study Director： Bin Cao

Team Members： Liang Chen、Hu Li、Meng Liu、Xiudi Han、Xiaoli Zhu 、Bo Liu、Jinxiang Wang、Xuexin Yao、Chunxiao Zhang、Shujing Shi、Fei Zhou、Chunxue Xue、Yanli Li、Donghao Yu （Beijing Chao-Yang Hospital 001；Beijing Jishuitan Hospital 002；Beijing Luhe Hospital 003；Qingdao Municipal Hospital 004;Qilu Hospital Of Shandong University(Qindao) 005； Beijing Huimin Hospital 006；Linzi District People's Hospital 007；The 2nd Hospital of Beijing Corps,Chinese Armed Police Forces 008； China-Japan Friendship Hospital 009;Yan'an Hospital Affliated to Kunming Medical University 010）

For peer review only

Inclusion Criteria：

1. **Age ≥14 years old**
2. **Onset in community**
3. **Chest X-ray or CT scan showing infiltration or interstitial changes, with or without pleural effusion**
4. **Any one of pneumonia clinical manifestations, including：**

(a) Recent cough, sputum or aggravation of respiratory symptoms, the emergence of purulent sputum, with or without chest pain;

(b) Fever (temperature ≥37.3°C) or hypothermia (temperature <36 °C);

(c) Signs of pulmonary consolidation and (or) moist rales;

(d) WBC>10×10^9/L, or <4×10^9/L, with or without nucleus left.

Meet criteria 1,2 ,3 and anyone of criteria 4

Exclusion Criteria：

1. **Lung infiltrate or interstitial changes which can be interpreted as lung cancer, pulmonary tuberculosis, non-infectious interstitial lung diseases, pulmonary edema, atelectasis, pulmonary embolism, pulmonary eosinophil infiltration, pulmonary vasculitis;**
2. **HIV positive**
3. **Readmission within 72 hours after discharging.**

For peer review only

For peer review only - http://bmjopen.bmj.com/site/about/guidelines.xhtml

Part 1：Baseline Characteristics

Underlying Disease	
COPD ○Y ○N	Asthma ○Y ○N
Bronchiectasis ○Y ○N	Malignancy ○Y ○N
Sleep Apnea Syndrome ○Y ○N	Congestive Heart Failure ○Y ○N
Coronary Heart Disease ○Y ○N	Hypertention ○Y ○N
Peripheral Vascular Diseases ○Y ○ N	Diabetes Mellitus ○Y ○N
Cerebrovascular Disease ○Y ○N	Autoimmune Diseases [a] ○Y ○ N
Chronic Viral Hepatitis ○Y ○N	Cirrhosis ○Y ○N
Hematological Malignancy ○Y ○N	Organ /bone Marrow Transplantation ○Y ○N
Immunosuppressive Therapy [b] ○Y ○N	Chemotherapy/Radiotherapy Within 6 Months ○Y ○N
Chronic Renal Diseases ○Y ○N	Splenectomy ○Y ○N

Note：aSLE, Sjogren's syndrome, rheumatoid arthritis, polymyositis / dermatomyositis, systemic vasculitis, ankylosing spondylitis, inflammatory bowel disease, hyperthyroidism, etc;； b.Anti-rejection drugs

With The Following Situation	
Pregnancy	○Y ○N ○Unknown；<br>If Y，Pregnancy__weeks。
Within 6 months after delivery	○Y ○N ○Unknown；<br>If Y，__weeks after delivery
Smoking	○Y ○N ○Former Smoker ○Unknown<br>If Y，Smoked For_years，__cigarettes/day；<br>If Former Smoker，Smoked For__years，<br>__cigarettes/day ,GivenUp For____years
Alcoholism [a]	○Y ○N ○Unknown
Risk factors for inhalation [b]	○Y ○N ○Unknown
Contact Children In Day-care Center	○Y ○N ○Unknown
Bed Ridden（≥2months）	○Y ○N ○Unknown
Long-term inhaled Corticosteroid use[d]	○Y ○N ○Unknown
Long-term oral Corticosteroid use[c]	○Y ○N ○Unknown；<br>If Y，Name Of Corticosteroid：______,

For peer review only - http://bmjopen.bmj.com/site/about/guidelines.xhtml

	Dose__mg/day，For_____days
Oral Statin Drugs	○Y ○N ○Unknown
History Of CAP Within One Year	○Y ○N ○Unknown
Influenza Vaccine Within 1 Year	○Y ○N ○Unknown
Streptococcus pneumoniae Vaccine Within 5 Years	○Y ○N ○Unknown

Note：**a**：drinking more than 5 bottles of beer (500ml / bottle) or half a catty liquor once in 2 weeks; or drinking more than 2.5 bottles of beer (500ml / bottle) or 2 ounc of white spirit per day for more than five years；**b:** Inhalation risk factors included choking, drowning, nasal, pseudobulbar palsy, dementia, coma, poisoning, Parkinson's disease, etc； **c:** Long-term oral corticosteroids was defined as: oral prednisone ≥10mg / d or equivalent doses of other corticosteroids for more than 3 weeks;**d**：Long-term inhaled corticosteroids was defined as: inhaled corticosteroid for more than 30 days, the daily dose wasn't limited.

Risk Factors Of Health-Care Acquired Pneumonia	
Hospitalization For 2d Or More In The Preceding 90 Days	○Y ○N ○Unknown
Home Infusion Therapy (Including Antibiotics) Or Home Wound Care In 30 Days	○Y ○N ○Unknown
Chronic dialysis within 30 Days	○Y ○N ○Unknown
Residence In A Nursing Home Or Extended Care Facility	○Y ○N ○Unknown

Part 2：**Data of This Hospitalization**

1.Signs And Symptoms

History Of Present Illness	
Clinical Manifestation	
Date Of Illness Onset ：_______Y_____M_____D	
Fever？ （T≥37.3 ℃）	○Y ○N；If Y，Tmax：____℃
Hypothermia？ （T＜36℃）	○Y ○N；If Y，Tmin：____℃
Cough？	○Y ○N
Sputum？	○Y ○N；<br>If Y，○Yellow Phlegm ○White Phlegm<br>○Bloody Sputum ○Unknown
Chest Pain？	○Y ○N
Shortness Of Breath？	○Y ○N
Sore Throat Or Rhinorrhea	○Y ○N
Chill/Shiver	○Y ○N
Exhaustion/	○Y ○N

For peer review only

For peer review only - http://bmjopen.bmj.com/site/about/guidelines.xhtml

Muscle And Joint Aches//Headache	
Darrhea?	○Y ○N
Familial Aggregation（2 Epidemiological Related People Suffered From Pneumonia In Two Weeks）?	○Y ○N
Physical Examination (The Worst Value Of The Day On Admission）	
Tmax，℃	
Tmin，℃	
HR, beats/min	
RR, breaths/min	
BP(Systolic Pressure / Diastolic Pressure), mmHg	
Disorder Of Consciousness?	○Y ○N
Cyanosis?	○Y ○N
Physical Signs Of Lung:	Moist rales ○Y ○N<br>Dry rales ○Y ○N
Edema Of Legs?	○Y ○N;<br>If Y，Asymmetric Edema Of Legs? ○Y ○N

3.Pre-hospital Medical Data ○Y ○N

Radiology		
Chest X-ray<br>○Y<br>○N<br>○Unknown<br>Date of Examination:<br>___Y__M_D	Site Of Pneumonia	○Bilateral Lung ○Unilateral Lung
	Site Of Pneumonia	○Superior Lobe Of Right Lung<br>○Middle Lobe Of Right Lung<br>○Inferior Lobe Of Right Lung<br>○Superior Lobe Of Left Lung<br>○Inferior Lobe Of Left Lung<br>○Unknown
	Plural effusion	○N ○Left ○Right ○Bilateral
	Cavity	○Y ○N
	consolidation	○Y ○N
	Interstitial Change	○Y ○N
	Infiltration	○Y ○N
Lung CT<br>○Y<br>○N<br>○Unknown<br>Date of Examination:<br>___Y__M_D	Alveolar Infiltration	○Superior Lobe Of Right Lung<br>○Middle Lobe Of Right Lung<br>○Inferior Lobe Of Right Lung<br>○Superior Lobe Of Left Lung<br>○Inferior Lobe Of Left Lung<br>○Bilateral Diffuse Infiltration<br>○Unilateral Diffuse iInfiltration

For peer review only - http://bmjopen.bmj.com/site/about/guidelines.xhtml

	Plural effusion	○N ○Left ○Right ○Bilateral		
	Cavity	○Y ○N		
	consolidation	○Y ○N		
	Abscesses	○Y ○N		
	Patchy Shadow	○Y ○N		
	Interstitial change	○Y ○N		
**Microbiological Examination**				
**Microbiological Examination Before Admission** ○Y ○N ○Unknown				
**If Y：**<br>**Date Of Specimen Collection：_____Y____M____D**<br>**Specimen Type: ○Sputum ○Blood ○BALF ○Asopharyngeal Swab**<br>**○Endotracheal Aspirate ○Plural Effusion ○Urine**<br>**Microbiological Examination Results:___________________________**				
**Treatment Before Admission**				
**Antimicrobials Before Admission** ○Y ○N ○Unknown				
**Drug name（Generic Name And Trade Name）**	**Route Of Administration**	**Drug Regimen**	**Start Time**	**TerminalTime**
eg：Ceftriaxone（罗氏芬）	● Intravenous ○Oral	2.0g ，Qd	2014．9．1	2014．9．8
	○ Intravenous ○Oral			
	○ Intravenous ○Oral			
	○ Intravenous ○Oral			
	○ Intravenous ○Oral			
**Antiviral Drug Use Before Admission** ○Y ○N ○Unknown				
	○Intravenous ○Oral<br>○ Inhalation			
	○Intravenous ○Oral<br>○ inhalation			
**Corticosteroid Use Before Admission** ○Y ○N ○Unknown				
	○Intravenous ○Oral<br>○ inhalation			
	○Intravenous ○Oral<br>○ inhalation			
**Vasopressor Use Before Admission** ○Y ○N ○Unknown				
**If Y，Start Time：____________** **Terminal Time：____________**				
**Invasive Ventilation Before Admission** ○Y ○N ○Unknown				
**If Y，Start Time：____________** **Terminal Time：____________**				

For peer review only

For peer review only - http://bmjopen.bmj.com/site/about/guidelines.xhtml

Note:b.Vasopressors：Norepinephrine、Dopamine、Dobutamine、Metaraminol、adrenaline

4.Laboratory Examination In 24hr On Admission

Category	Item	Value	Unit
Blood Routine	WBC		*10^9/L
	Neu		*10^9/L
	Lym		*10^9/L
	HGB		g/L
	HCT		%
	PLT		*10^9/L
Biochemistry	ALB		g/L
	LDH		U/L
	AST		U/L
	ALT		U/L
	ALP		U/L
	TBIL		umol/L
	DBIL		umol/L
	CK		U/L
	BUN		mmol/L
	Cr		mmol/L
	Glu		mmol/L
	K		mmol/L
	Na		mmol/L
Serum Detection	ESR		mm/h
	CRP		mg/dL
	PCT		ng/ml
	D-dimer		ng/ml
	PT		s
	APTT		s
	INR		
	BNP		pg/ml
	Ferritin		ug/l

5.Blood Gas Analysis、Radiology and Ultrasonography After Admission

Category	Item	Value

For peer review only - http://bmjopen.bmj.com/site/about/guidelines.xhtml

Blood gas analysis (The Worst Value In 24hr On Admission)	Oxygen Therapy※<br>○Y ○N	○Oxygen Inhalation Through Nasal Tube____L/min<br>○Oxygen Inhalation Through Venturi Mask_____%<br>○Oxygen Inhalation Through Oxygen Masks__L/min<br>○Non-invasive Ventilation<br>○Invasive Ventilation<br>○Unknowen	
	FiO_2		
	pH		
	PO_2(mmHg)		
	PCO_2(mmHg)		
	SaO_2		
	Actual Bicarbonate (mmol/l)		
	Lac (mmol/l)		
Radiology (In 24hr On Admission)	Chest X-ray<br>○Y ○N	Alveolar Infiltration	○Superior Lobe Of Right Lung<br>○Middle Lobe Of Right Lung<br>○Inferior Lobe Of Right Lung<br>○Superior Lobe Of Left Lung<br>○Inferior Lobe Of Left Lung<br>○Bilateral Diffuse Infiltration<br>○Unilateral Diffuse Infiltration
		Plural effusion	○N ○Left ○Right<br>○Bilateral
		Cavity	○Y ○N
		consolidation	○Y ○N
		Patchy Shadow	○Y ○N
		Interstitial Change	○Y ○N
	Lung CT<br>○Y ○N	Alveolar Infiltration	○Superior Lobe Of Right Lung<br>○Middle Lobe Of Right Lung<br>○Inferior Lobe Of Right Lung<br>○Superior Lobe Of Left Lung<br>○Inferior Lobe Of Left Lung<br>○Bilateral Diffuse Infiltration<br>○Unilateral Diffuse Infiltration
		Plural effusion	○N ○Left ○Right<br>○Bilateral
		Cavity	○Y ○N
		consolidation	○Y ○N
		Patchy Shadow	○Y ○N

For peer review only - http://bmjopen.bmj.com/site/about/guidelines.xhtml

		**Interstitial Change**	**○Y ○N**
		**Alveolar Infiltration**	**○Superior Lobe Of Right Lung**<br>**○Middle Lobe Of Right Lung**<br>**○Inferior Lobe Of Right Lung**<br>**○Superior Lobe Of Left Lung**<br>**○Inferior Lobe Of Left Lung**<br>**○Bilateral Diffuse Infiltration**<br>**○Unilateral Diffuse Infiltration**
**Ultrasonography**	**Lower Limb Vascular Ultrasound Exam**	**Venous Thrombosis**	**○N ○Left ○Right**<br>**○Bilateral ○ Unexamined**

Note※The Worst Value Of Blood Gas Analysis And FiO2 At That Time.

For peer review only

6.Keep Detailed Records Of The Following Time Points,And Write down The Code In The First Row Of The Table：

①The 4th day（The Day On Admission Is The 1st Day）；②The day of changing Antibiotics in 14 days After Admission；③The 14th day after Admission ；④The Day Of Discharging

For peer review only

For peer review only - http://bmjopen.bmj.com/site/about/guidelines.xhtml

Category	Item(Unit)	The Reason and The Date						
Vital Signs	Disorder Of Consciousness							
	Tmax（℃）							
	Tmin(℃)							
	HR（beats/min）							
	RR（breaths/min）							
	BP(/ mmHg）							
Symptoms<br>1. Exacerbation<br>2. Alleviation<br>3. No-change	Cough							
	Sputum							
	Chest Pain							
	Shortness Of Breath							
	Moist Rales							
	Dry Rales							
Blood Routine	WBC（*10^9/L）							
	Neu（*10^9/L）							
	Lym（*10^9/L）							
	HGB（g/L）							
	HCT（%）							
	PLT（*10^9/L）							
Biochemistry	ALB（g/L）							
	LDH（U/L）							
	AST（U/L）							
	ALT（U/L）							
	ALP（U/L）							
	TBIL（umol/L）							
	DBIL（umol/L）							
	CK（U/L）							
	CTNI（ng/ml）							
	BUN（mmol/L）							
	Cr（mmol/L）							
	[illegible]							

For peer review only

For peer review only - http://bmjopen.bmj.com/site/about/guidelines.xhtml

Note：a. Direct Microscopy of sputum is not included

For peer review only

For peer review only - http://bmjopen.bmj.com/site/about/guidelines.xhtml

7.Treatment During Hospitalization

Antibiotics Use ○Y ○N				
Drug Name (Generic Name And Trade Name)	Route Of Administration	Drug Regimen	Start Time	Terminal Ttime
eg: Ceftriaxone (罗氏芬)	● Intravenous ○Oral	2.0g，Qd	2014-3-2	2014-4-5
	○ Intravenous ○Oral			
	○ Intravenous ○Oral			
	○ Intravenous ○Oral			
	○ Intravenous ○Oral			
	○ Intravenous ○Oral			
	○ Intravenous ○Oral			
	○ Intravenous ○Oral			
Antiviral Drugs Use ○Y ○N				
Drug name (Generic Name And Trade Name)	Route Of Administration	Drug Regimen	Start Time	Terminal Time
	○ Intravenous ○Oral ○ Inhalation			
	○ Intravenous ○Oral ○ Inhalation			
	○ Intravenous ○Oral ○ Inhalation			
Glucocorticoids Use ○Y ○N				
Drug name (Generic Name And Trade Name)	Route Of Administration	Drug Regimen	Start Time	Terminal Time
	○ Intravenous ○Oral ○Inhalation			
	○ Intravenous ○Oral ○ Inhalation			
Vasopressors Use ○Y ○N				
Drug Name	Route Of Administration	Drug Regimen	Start Time	Terminal Ttime
Immunoregulation Drugs（Including Intravenous Immunoglobulin 、Thymosins）○Y ○N				
Drug Name	Route of administration	Drug Regimen	Start time	Terminal time

For peer review only

For peer review only - http://bmjopen.bmj.com/site/about/guidelines.xhtml

Alternative/ Supportive Treatment			
Item	Use	Start Time	Terminal Time
Continuous Venous-venous Hemofiltration	○Y ○N		
Extracorporeal Membrane Oxygenation （ECMO）	○Y ○N		
Non-invasive Ventilation	○Y ○N		
Invasive Ventilation	○Y ○N		

8、Measurement Of T Lymphocyte Subsets

Date of specimen collection: __Y ___M ___D

T lymphocyte subsets	CD4		/ml
	CD8		/ml
	CD4%		
	CD8%		
	NK		/ml
	NKT		/ml
	CD4/CD8		

Note：Without Time Limitation

9.Microbiological Examination

(1).Microbiological Examination In 48hrs After Admission ○Y ○N

Microbiological Examination For Sputum Or eEndotracheal aAspiration<br>Date Of Specimen Collection：__Y__M__D	
Item	Results
Direct Microscopy	○Good Quality Sputum（ > 25 leukocytes and < 10 epithelial cells per × 100 magnification field）<br>○Not Good Quality Sputum<br>○Unknown
	○G+ Cocci ○G+ Bacillus ○G- Cocci<br>○G- Bacillus ○Positive Acid-fast Stain ○None

For peer review only - http://bmjopen.bmj.com/site/about/guidelines.xhtml

Bacteria Culture	○Streptococcus pneumoniae ○Moraxella catarrhalis<br>○Haemophilus influenzae ○staphylococcus aureus<br>○Pseudomonas aeruginosa ○Klebsiella pneumoniae<br>○Enterobacter cloacae ○Proteus spp<br>○Acinetobacter spp ○Serratia marcescens<br>○Stenotrophomonas maltophilia ○Enterobacter aerogenes<br>○Escherichia coli ○Enterococcus faecalis<br>○Enterococcus faecium ○Others: ____<br>○None Or Normal oropharyngeal flora
	Drug Resistant Bacteria
	○Methicillin Resistance Staphylococcus aureus（MRSA)<br>○Vancomycin-resistant Enterococcus<br>Bacteria producing ESBLs:<br>○Escherichia coli ○Klebsiella pneumoniae<br>○Enterobacter cloacae ○Serratia marcescens<br>non - fermentative bacteria.:<br>○Acinetobacter baumannii ○Pseudomonas aeruginosa<br>○Others: ____
	If Streptococcus pneumoniae ， MIC for penicillin___ug/ml;<br>○Not detected
	If MRSA，MIC for Vancomycin____ug/ml;<br>○Not detected
Direct Microscopy	○Fungal Spore ○Fungal Hyphae<br>○Cryptococcus neoformans ○None
Fungi Culture	○Spergillus Fumigatus ○Aspergilusflavus<br>○Aspergillus terreus ○Mucor Mucedo<br>○Candida Spp ○Cryptococcus Neoformans<br>○Undetected ○Others: ____
Nucleic Acid Test For Respiratory Virus	○Influenza A H1N1 ○Avian influenza H7N9<br>○Influenza A H2N3 ○Influenza A H5N1<br>○Nontypeable Influenza A ○Influenza B<br>○Adenovirus ○Parainfluenza virus 1<br>○Parainfluenza virus2 ○Parainfluenza virus 3<br>○Parainfluenza virus 4 ○Respiratory syncytial virus A<br>○Rhinovirus ○Respiratory syncytial virus B<br>○Coronavirus OC43HKU1 ○Enterovirus<br>○Coronavirus 229ENL63 ○Herpes simplex virus<br>○Bocavirus ○Cytomegalovirus<br>○EB virus ○MERS-CoV

For peer review only

Nucleic Acid Test For Atypical Etiology	○Mycoplasma pneumoniae<br>○Legionella spp	○Chlamydia pneumoniae

(2).Microbiological Examination For BALF? ○Y ○N

Microbiological examination for BALF（Within One Week After Admission）<br>Date Of Specimen Collection：__Y__M__D	
Item	Results
Direct Microscopy	○G+ Cocci ○G+ Bacillus ○G- Cocci<br>○G- Bacillus ○Positive Acid-fast Stain ○None
Bacteria Culture	○Streptococcus pneumoniae ○Moraxella catarrhalis<br>○Haemophilus influenzae ○staphylococcus aureus<br>○Pseudomonas aeruginosa ○Klebsiella pneumoniae<br>○Enterobacter cloacae ○Proteus spp<br>○Acinetobacter spp ○Serratia marcescens<br>○Stenotrophomonas maltophilia ○Enterobacter aerogenes<br>○Escherichia coli ○Enterococcus faecalis<br>○Enterococcus faecium ○Others：____<br>○None Or Normal oropharyngeal flora
	Drug Resistant Bacteria
	○Methicillin Resistance Staphylococcus aureus（MRSA）<br>○Vancomycin-resistant Enterococcus<br>Bacteria producing ESBLs：<br>○Escherichia coli ○Klebsiella pneumoniae<br>○Enterobacter cloacae ○Serratia marcescens<br>non - fermentative bacteria.：<br>○Acinetobacter baumannii ○Pseudomonas aeruginosa<br>○Others：___
	If Streptococcus pneumoniae ， MIC for penicillin___ug/ml；<br>○Not Detected
	If MRSA，MIC for Vancomycin____ug/ml；<br>○Not Detected
Direct Microscopy	○Fungal Spore ○Fungal Hyphae<br>○Cryptococcus neoformans ○None

For peer review only - http://bmjopen.bmj.com/site/about/guidelines.xhtml

**Fungi Culture**	**○Spergillus Fumigatus ○Aspergilusflavus ○Aspergillus terreus ○Mucor Mucedo ○Candida Spp ○Cryptococcus Neoformans ○Undetected ○Others: ___**
**Nucleic Acid For Respiratory Virus**	**○Influenza A H1N1 ○Avian influenza H7N9 ○Influenza A H2N3 ○Influenza A H5N1 ○Nontypeable Influenza A ○Influenza B ○Adenovirus ○Parainfluenza virus 1 ○Parainfluenza virus2 ○Parainfluenza virus 3 ○Parainfluenza virus 4 ○Respiratory syncytial virus A ○Rhinovirus ○Respiratory syncytial virus B ○Coronavirus OC43HKU1 ○Enterovirus ○Coronavirus 229ENL63 ○Herpes simplex virus ○Bocavirus ○Cytomegalovirus ○EB virus ○MERS-CoV**
**Nucleic Acid Test For Atypical Etiology**	○Mycoplasma pneumoniae **○Chlamydia pneumoniae** ○Legionella spp

(3).Blood Culture In One Week After Admission? ○Y ○N

**Blood Culture (In One Week After Admission) Date Of Specimen Collection: __Y__M__D**	
**Item**	**Results**
**Bacteria Culture**	**○Staphylococcus aureus ○Moraxella catarrhalis ○Haemophilus influenzae ○Pseudomonas aeruginosa ○Klebsiella pneumoniae ○Enterobacter cloacae ○Proteus spp ○Acinetobacter spp ○Serratia marcescens ○Stenotrophomonas maltophilia ○Enterobacter aerogenes ○Escherichia coli ○Enterococcus faecalis ○Enterococcus faecium ○Others: _________ ○None or normal oropharyngeal flora**
	**Drug Resistant Bacteria**
	**○Methicillin resistance staphylococcus aureus (MRSA) ○Vancomycin-resistant Enterococcus Bacteria producing ESBLs: ○Escherichia coli ○Klebsiella pneumoniae ○Enterobacter cloacae ○Serratia marcescens non - fermentative bacteria.: ○Acinetobacter baumannii ○Pseudomonas aeruginosa ○Others: ___**

For peer review only - http://bmjopen.bmj.com/site/about/guidelines.xhtml

	If Streptococcus pneumoniae ，MIC for penicillin___ug/ml；<br>○Not Detected
	If MRSA，MIC for Vancomycin____ug/ml；<br>○Not Detected
Fungi Culture	○Candidiasis albicans ○Candida krusei<br>○Candida tropicalis ○Candida glabrata<br>○Candida parapsilosis ○Cryptococcus neoformans<br>○Aspergillus fumigatus ○Aspergilus flavus<br>○Aspergillus terreus ○Mucor Mucedo<br>○Undetected ○Others：___

（4）、Pleural Effusion？ ○Y ○N

Pleural Effusion Test？ ○Y ○N

Microbiological Examination For Pleural Effusion（Without Time Limitation）<br>Date of Specimen collection： _Y__M__D	
Pleural Effusion Routine	
Total Cell Count： __$\times10^6$/L；Multinuclear Cell： __$\times10^6$/L；<br>Mononuclear Cells： __$\times10^6$/L	
Pleural Effusion Biochemistry	
LDH： __U/L； ADA： __U/L； Pr： __g/L<br>Glu:__mmol/L Cl: __mmol/L	
Item	Results
Bacteria Culture	○Staphylococcus aureus ○Moraxella catarrhalis<br>○Haemophilus influenzae ○Pseudomonas aeruginosa<br>○Klebsiella pneumoniae ○Enterobacter cloacae<br>○Proteus spp ○Acinetobacter spp<br>○Serratia marcescens ○Stenotrophomonas maltophilia<br>○Enterobacter aerogenes ○Escherichia coli<br>○Enterococcus faecalis ○Enterococcus faecium<br>○Others： _________ ○None or Normal Oropharyngeal Flora
	Drug Resistant Bacteria
	○Methicillin resistance staphylococcus aureus（MRSA)<br>○Vancomycin-resistant Enterococcus<br>Bacteria producing ESBLs：<br>○Escherichia coli ○Klebsiella pneumoniae<br>○Enterobacter cloacae ○Serratia marcescens<br>non - fermentative bacteria.：<br>○Acinetobacter baumannii ○Pseudomonas aeruginosa<br>○Others： ___
Fungi Culture	○Candidiasis albicans ○Candida krusei<br>○Candida tropicalis ○Candida glabrata

For peer review only - http://bmjopen.bmj.com/site/about/guidelines.xhtml

	○Candida parapsilosis	○Cryptococcus neoformans
	○Aspergillus fumigatus	○Aspergilus flavus
	○Aspergillus terreus	○Mucor Mucedo
	○Undetected	○Others：_

（5）、Antigen Test In 48hr After Admission？ ○Y ○N

Urinary antigen（in 48hr after admission）<br>Date of specimen collection：__Y___M___D	
Urinary Antigen For *Legionella spp*	○Positive ○Negative ○Undetected
Urinary Antigen For *Streptococcus pneumoniae*	○Positive ○Negative ○Undetected
**Throat Swab Aantigen Test（In 48hr After Admission）<br>Date Of Specimen Collection：__Y___M___D**	
Respiratory Syncytial Virus Antigen Test	○Positive ○Negative ○Undetected
Influenza A Antigen Test	○Positive ○Negative ○Undetected
Influenza B Antigen Test	○Positive ○Negative ○Undetected

（6）、Antibody Test？ a）○Y ○N

b）If Y , Titer Of Antibody In Paired Serum? ○Y ,Interval__days

○N

Antibody Test（Without Time Limitation）<br>Date Of Specimen Collection：__Y___M___D	
○IgM for *Mycoplasmal pneumonia*	○IgM for Influenza A
○IgG for *Mycoplasmal pneumonia*	○IgM for Parainfluenza
○IgM for *Chlamydia* spp	○IgM for Q fever
○IgG for *Chlamydia* spp.	○IgM for Adenovirus
○IgM for *Legionella spp*	○IgM for Respiratory syncytial virus
○IgG for *Legionella spp*	○IgM for Parainfluenza 1,2,3

For peer review only - http://bmjopen.bmj.com/site/about/guidelines.xhtml

10、Outcomes

(1).Treatment Failure Within 14 Days

Treatment Failure Within 14 Days（Multiple choices）<br>（The Value Of The 1st Day On Admission As The Baseline Data）	
**1.Needs Invasive Ventilation**	○Y ○N
**2.Needs Non-invasive Ventilation**	○Y ○N
**3.Needs Vasopressors**	○Y ○N
**4.Death**	○Y ○N

The Reasons For Treatment Failure	
**1.CAP Progression**	Pneumonia Progression ○Y ○N
**2.CAP Complications**	Pyothorax ○Y ○N<br>Endocarditis ○Y ○N<br>Meningitis ○Y ○N<br>Others：__________
**3.Severe Sepsis Due To CAP**	ARDS ○Y ○N<br>Sepsis ○Y ○N<br>Hepatic Failure ○Y ○N<br>Renal Ffailure ○Y ○N<br>Clotting Disorders, ○Y ○N<br>Encephalopathy ○Y ○N<br>Others：________
**4.Complications Or Underlying Disease Deterioration**	Pulmonary Embolism ○Y ○N<br>Myocardial Infarction ○Y ○N<br>Arrhythmia ○Y ○N<br>Gastrointestinal Bleeding ○Y ○N<br>Congestive Heart Failure ○Y ○N<br>COPD ○Y ○N<br>Diabetes Mellitus ○Y ○N<br>Nephropathy ○Y ○N<br>Others：________
**5.Complications Due To Treatment**	Hemopneumothorax ○Y ○N<br>Allergic To Antibiotics ○Y ○N<br>HAP/VAP ○Y ○N<br>Vascular Catheter Infection ○Y ○N<br>C. Difficile Infection ○Y ○N<br>Iatrogenic Urinary Tract Infection ○Y ○N<br>Others：________
**6.Unknown**	○Y ○N

For peer review only

(2). Complications During Hospitalization

Complications During Hospitalization			
Complications（Multiple Choices）	○Y ○N		
Respiratory Failure	○Y ○N	ARDS	○Y ○N
Heart Failure	○Y ○N	Acute Myocardial Infarction	○Y ○N
Acute Liver Failure	○Y ○N	AcuteRenal Failure	○Y ○N
Septic Shock	○Y ○N	Stroke	○Y ○N
DIC	○Y ○N	Antibiotic Associated Diarrhea	○Y ○N
Arrhythmia	○Y ○N	MODS	○Y ○N
Pulmonary Embolism	○Y ○N	Deep Venous Thrombosis	○Y ○N
Ventilator Associated Pneumonia	○Y ○N	Gastrointestinal Bleeding	○Y ○N
Invasive Aspergillosis	○Y ○N	Mediastinal Emphysema	○Y ○N
Pneumothorax	○Y ○N	Nosocomial Bloodstream Infection	○Y ○N
Others	○Y ○N	If Y: _______	

（3）. Outcomes

Clinical Stability Before Discharge	○Y ○N<br>If Y，the date of clinical stability ______Y ______M ______D.<br>Meet the following seven criteria：Temperature<37.8℃ for more than 24hr；Heart rate ≤100 beats/min ；Respiratory rate ≤24 breaths/min ;Systolic blood pressure ≥90 mm Hg ； Arterial oxygen saturation ≥90% or pO2 ≥ 60 mm Hg on room air ； Ability to maintain oral intake；Normal mental status.
Admitted to RICU/ICU?	○Y ○N<br>If Y，The Date Of Admitted To RICU/ICU：___Y___M___D<br>The Date Of Transfer From RICU/ICU：___Y___M___D
Discharging	The Date Of Discharging___Y___M___D<br>Outcome ○Improvement ○Against-advice discharge<br>○Death<br>If death，The Death Date____Y___M___D
Direct Cause Of Death (only one choice)	○Severe Pneumonia ○Respiratory Failure<br>○Shock○Heart Failure ○Acute Myocardial Infarction

For peer review only - http://bmjopen.bmj.com/site/about/guidelines.xhtml

	○Acute renal Failure ○Hepatic failure<br>○DIC ○Stroke<br>○Gastrointestinal Bleeding ○Others: _______

10. Cost And Economy Data

Total Expenses ______Yuan:

Drugs Cost: _________Yuan , Antimicrobials Cost ________Yuan

Laboratory Testing Expenses: _______Yuan

Bed Charge: _________Yuan

Health Care Worker Labor Cost: ________Yuan

For peer review only

For peer review only - http://bmjopen.bmj.com/site/about/guidelines.xhtml

Appendix 4: Definition of underlying diseases

1) Long-term smoking was defined as: cigarette smokers of 10 cigarettes/d during at least the previous year；

2) Alcoholism was defined as: drinking more than 5 bottles of beer (500ml / bottle) or half a catty liquor once in 2 weeks; or drinking more than 2.5 bottles of beer (500ml / bottle) or 2 ounc of white spirit per day for more than five years；

3) Long-term oral corticosteroids was defined as: oral prednisone ≥10mg / d or equivalent doses of other corticosteroids for more than 3 weeks [1].

4) Long-term inhaled corticosteroids was defined as: inhaled corticosteroid for more than 30 days, the daily dose wasn't limited;

5) COPD was defined as: persistent airflow limitation, FEV1 / FVC < 70% post bronchodilator;

6) Asthma was defined by the history of respiratory symptoms such as wheeze, cough that varied over time and intensity, together with variable respiratory airway limitation;

7) Hypertension was defined as systolic blood pressure≥140mmHg and /or diastolic blood pressure≥90mmHg in resting status;

8) Coronary heart disease included angina pectoris, myocardial infarction, ischemic cardiomyopathy;

9) Chronic congestive heart failure was defined as cardiomegaly and ejection fraction ≤40%;

10) Cerebrovascular diseases included transient ischemic attack, cerebral hemorrhage, subarachnoid hemorrhage, cerebral infarction, etc;

For peer review only - http://bmjopen.bmj.com/site/about/guidelines.xhtml

11) Diebetes mellitus: included diabetes mellitus type 1 and diabetes mellitus type 2, not included impaired glucose tolerance and impaired fasting glycaemia;

12) Chronic liver disease included chronic viral hepatitis, chronic alcoholic liver disease, chronic fatty liver disease, etc;

13) Chronic kidney disease included diabetic nephropathy, hypertensive renal damage, chronic glomerulonephritis, chronic pyelonephritis, lupus nephritis, IgA nephropathy, nephrotic syndrome, hereditary kidney disease, etc;

14) Connective Tissue Diseases include SLE, Sjogren's syndrome, rheumatoid arthritis, polymyositis / dermatomyositis, systemic vasculitis, ankylosing spondylitis, inflammatory bowel disease, hyperthyroidism, etc;

15) Organ transplantation or bone marrow transplantation included solid organ transplantating, such as liver transplantation, kidney transplantation, lung transplantation or pancreas transplantation, etc and bone marrow transplantation;

16) Aspiration risk factors included choking, drowning, nasal, pseudobulbar palsy, dementia, coma, poisoning, Parkinson's disease, etc.

17) Immunosuppressive therapy: was defined as systmatic glucocorticosteroid (such as prednisone ≥10mg/d for more than 3 weeks in the last month); cyclosporine or azathioprine use within 3 months, and methotrexate use ≥12.5mg/week within 3 months; biological modifiers such as etanercept and infiximab within 3 weeks.

18) Immunocompromised status included chemotherapy/radiotherapy within 6 months, immunosuppressive therapy, organ/bone marrow transplantation, splenectomy, hematological neoplasms [2].

For peer review only - http://bmjopen.bmj.com/site/about/guidelines.xhtml

19) Risk factors for pseudomonal infection was defined as chronic airway disease (bronchiectasis or COPD), immunocompromised status and HCAP risk factors according to IDSA/ATS criteria [3-7] .

20) Overtreatment was defined as: i) use of antipseudomonal β-lactams or β-lactams+ fluoquinolones in patients aged <65yr without risk factors for pseudomonal infection; i i) use of (antipseudomal or not)β-lactams+ fluoquinolones in patients aged≥ 65yr without risk factors for pseudomonal infection and not in ICU [3].

References:

1. Jouneau S, Poineuf JS, Minjolle S, et al. Which patients should be tested for viruses on bronchoalveolar lavage fluid? Eur J Clin Microbiol Infect Dis. 2013 May;32(5):671-7.

2. Sousa D, Justo I, Domínguez A,et al. Community-acquired pneumonia in immunocompromised older patients: incidence, causative organisms and outcome. Clin Microbiol Infect. 2013 Feb;19(2):187-92

3. Guidelines for the diagnosis and treatment of adult community acquired pneumonia in China(2016 Edition)]. Zhonghua Jie He He Hu Xi Za Zhi. 2016 Apr 12; 39 (4): 241-2

4. American Thoracic Society.Infectious Diseases Society of America.Guidelines for the management of adults with hospital-acquired, ventilator-associated, and healthcare-associated pneumonia.Am J Respir Crit Care Med. 2005 Feb 15; 171(4):388-416

For peer review only - http://bmjopen.bmj.com/site/about/guidelines.xhtml

5. Rañó A, Agustí C, Benito N,et al. Prognostic factors of non-HIV immunocompromised patients with pulmonary infiltrates.Chest. 2002 Jul;122(1):253-61

6 .Al-Anazi KA, Al-Jasser AM.Infections Caused by Acinetobacter baumannii in Recipients of Hematopoietic Stem Cell Transplantation.Front Oncol. 2014 Jul 14;4:186-95

7. Rañó A, Agustí C, Benito N,et al. Prognostic factors of non-HIV immunocompromised patients with pulmonary infiltrates.Chest. 2002 Jul;122 (1):253-61

For peer review only

Appendix 5: Definition of microbiological criteria of CAP:

Definite, if one of the following criteria was met:

4. Positive urinary antigen for *Legionella pneumophila* (LP, Binax Now L

For peer review only - http://bmjopen.bmj.com/site/about/guidelines.xhtml

pneumophila urinary antigen test; Trinity Biotech, Bray, Ireland);

5. Positive urinary antigen for *Streptococcus pneumoniae* (Binax Now *S pneumoniae* urinary antigen test; Emergo Europe, The Netherlands);
6. Positive bacterial culture from blood or plural fluid except for coagulase negative *Staphylococcus spp*.
7. Paired sera with a fourfold or more increase in the titers of antibodies to *Mycoplasma pneumoniae* (MP), *Chlamydia pneumonia*, *L pneumophila or* respiratory viruses (Influenza A and B, Parainfluenza, Adenovirus, Respiratory syncytial virus). Or Serum IgM antibody (MIF) ≥ 1:16 for *Chlamydia pneumonia.*

Probable, if one of the following criteria was met:

a. Detection of respiratory virus in sputum/bronchoalveolar lavage (BALF)/throat swabs by Realtime-PCR (Zhijiang, Shanghai, China) according to manufacturer's instructions, including respiratory syncytial virus (RSV) types A and B, influenza virus (IFV) types A and B, parainfluenza virus (PIV) types 1, 2, 3 and 4, rhinovirus (HRV), enterovirus (EV), coronavirus (hCoV) types 229E, NL63, OC43 and HKU1, parapneumovirus (hMPV), and adenovirus (AdV), bocavirus;
b. Bacteria isolated form purulent sputum (defined as an adequate quality sputum sample with > 25 leukocytes and < 10 epithelial cells per × 100 magnification field) with compatible findings of Gram staining;
c. Detection of *Mycoplasma pneumoniae* (MP), *Chlamydia pneumonia* or *L pneumophila* in sputum/BALF/throat swabs by Realtime-PCR (Zhijiang, Shanghai, China)
d. Positive antigen for Influenza A/B (Alere ™, Clearview Exact Influenza A& B)
e. Serum IgM antibody positive for *Mycoplasma pneumoniae* (MP), or Serum IgG antibody (MIF) ≥ 1:512 for *Chlamydia pneumonia.*

For peer review only - http://bmjopen.bmj.com/site/about/guidelines.xhtml

Appendix 6: CAP patients with definite and probable microbiological diagnosis

For peer review only

For peer review only - http://bmjopen.bmj.com/site/about/guidelines.xhtml

Etiology	Without risk factors for pseudomonal infection (n=409)				With risk factors for pseudomonal infection (n=373)	Total (n=782)
	age<65yr and not in ICU (n=182)	age<65yr and in ICU (n=29)	age≥65yr and not in ICU (n=162)	age≥65yr and in ICU (n=36)		
**Bacterial**	146 (83·9%)	19 (4·6%)	144 (3·2%)	35 (8·6%)	31 (8·3%)	704 (90·0%)
Pseudomonas aeruginosa	27	0	31	6	146	210
Klebsiella pneumoniae	30	9	27	10	60	136
E. coli	15	1	17	2	34	69
Acinetobacter	13	3	20	3	27	66
Staphylococcus aureus	7	3	10	7	26	53
Enterobacter cloacae	9	1	8	3	17	38
Streptococcus pneumoniae	9	1	5	1	11	27
Stenotrophomonas	8	1	10	2	6	27
Enterococcus faecalis	5	0	3	0	13	21
Enterococcus faecium	3	0	1	0	5	9
others	20	3	18	7	40	88
**Atypical etiology**	7 (1·7%)	5 (1·2%)	3 (0·7%)	0 (0·0%)	0 (0·0%)	12 (1·5%)
Mycoplasma pneumoniae	6	0	1	0	0	7
Legionnella pneumoniae	0	1	2	0	0	3
Chlamydia pneumoniae	0	1	0	0	0	1
**Virus**	36 (8·8%)	15 (3·7%)	23 (5·6%)	2 (0·5%)	22 (5·9%)	98 (12·5%)
Influenza A virus	25	8	14	1	10	58
Rhinovirus	3	2	2	0	2	9
Influenza B virus	0	0	4	1	3	8
Adenovirus	6	1	0	0	0	7
Respiratory syncytial virus	1	0	0	0	0	1
Human metapneumovirus	0	0	1	0	0	1
Cytomegalovirus	1	1	0	0	3	5
**Bacterials+viruses**	4 (1·0%)	5 (1·2%)	7 (1·7%)	1 (0·2%)	9 (2·4%)	26 (3·3%)
**Viruses+atypical pathogens**	2 (0·5%)	2 (0·5%)	1 (0·2%)	0 (0·0%)	0 (0·0%)	5 (0·6%)

For peer review only

For peer review only - http://bmjopen.bmj.com/site/about/guidelines.xhtml

For peer review only

For peer review only - http://bmjopen.bmj.com/site/about/guidelines.xhtml

Appendix 7: Sub-group analysis of 30-day mortality

Severity of illness	30-day mortality	P value
CURB-65		<0.001
0’	25 (1·0%)	
1’	65 (2·9%)	
2’	96 (10·4%)	
3’	50 (32·5%)	
4’	6 (30·0%)	
5’	1 (50·0%)	
PSI risk class		<0.001
I	8 (0·7%)	
II	10 (1·1%)	
III	23 (3·0%)	
IV	70 (10·3%)	
V	59 (30·6%)	
Age		<0.001
14~64 ys	54 (1·8%)	
65~74 ys	49 (4·3%)	
75~89 ys	124 (6·9%)	
≥90 ys	30 (16·0%)	
Gender		0.005
Male	160 (4·9%)	
Female	97 (3·5%)	
Underlying Diseases		
None of any underlying disease	19 (0·3%)	
Organ/Bone marrow Transplantation	3 (23·1%)	<0.001
Immunosuppressive therapy	9 (17·3%)	<0.001
Chemotherapy/Radiology within 6 months	12 (16·9%)	<0.001

For peer review only

For peer review only - http://bmjopen.bmj.com/site/about/guidelines.xhtml

Hematological neoplasms	8 (14·8%)	<0.001
Chronic Renal diseases	32 (14·4%)	<0.001
Long-term oral corticosteroids use	14 (14·0%)	<0.001
Chronic congestive heart failure	23 (10·9%)	<0.001
Cerebrovascular Diseases	93 (10·1%)	<0.001
Malignant solid tumors	29 (9·4%)	<0.001
Coronary Heart Diseases	83 (6·9%)	<0.001
Diabetes	57 (6·0%)	<0.001
Hypertension	124 (5·8%)	<0.001
Connective Tissue Diseases	9 (5·3%)	0.001
Chronic Liver diseases	5 (5·2%)	0.009
COPD	38 (4·6%)	<0.001
Asthma	8 (2·5%)	0.126
Bronchiectasis	10 (1·5%)	0.536
ICU admission		
Yes	99 (25·3%)	<0.001
No	158 (2·8%)	
Systemic glucocorticosteroids use in admission		
Yes	97 (5·8%)	<0.001
No	160 (3·6%)	

For peer review only

For peer review only - http://bmjopen.bmj.com/site/about/guidelines.xhtml

For peer review only

For peer review only - http://bmjopen.bmj.com/site/about/guidelines.xhtml

Appendix 8 Empirical antimicrobial regimens according to Chinese CAP guideline

Populations	Common pathogens	Anti-infective agents for initial empirical therapy	Comment
Outpatient treatment (Oral administration is recommended)			
Young adults without underlying disease(s)	*S. pneumoniae*, *M. pneumoniae*, *H. influenzae*, *C. pneumoniae*, influenza virus, adenovirus, *M. catarrhalis*	(1) Aminopenicillins, penicillins-β-lactamase -inhibitor combinations; (2) I or II generation cephalosporins; (3) doxycycline or minocycline; (4) respiratory quinolones; (5) macrolides	(1) Differentiate among bacterial pneumonia, *Mycoplasma*, *Chlamydia* and viral pneumonia based on clinical characteristics; (2) Mild pneumonia caused by *Mycoplasma*, *Chlamydia*, and virus is usually self-limited
Patients with underlying disease(s) or elderly patients (age ≥ 65 years)	*S. pneumoniae*, *H. influenzae*, *Enterobacteriaceae* such as *K. pneumoniae*, *C. pneumoniae*, influenza virus, RSV, *M. catarrhalis*	(1) Penicillins-β-lactamase-inhibitor combinations; (2) II or III generation cephalosporins (oral); (3) respiratory quinolones; (4) penicillins-lactamase -inhibitor combinations, II generation cephalosporins, III generation cephalosporins combined with doxycycline or minocycline or macrolides	Monotherapy with doxycycline or minocycline or macrolides is not recommended in patients with risk factors of resistant *S. pneumoniae* (1), such as age > 65 years, underlying diseases (chronic cardiac, pulmonary, or renal diseases, diabetes mellitus, and immunosuppression), alcoholism, and β-lactams treatment within 3 months.
Inpatient treatment, non-ICU (Intravenous or oral administration)			

For peer review only - http://bmjopen.bmj.com/site/about/guidelines.xhtml

Young adults without underlying disease(s)	*S. pneumoniae*, *H. influenzae*, *M. catarrhalis*, *S. aureus*, *M. pneumoniae*, *C. pneumoniae*, influenza virus, adenovirus, other respiratory tract viruses	(1) Penicillin G, aminopenicillins, penicillins-β-lactamase-inhibitor combinations; (2) II or III generation cephalosporins, cephamycins, oxacephems; (3) the above drugs combined with doxycycline, minocycline or macrolides; (4) respiratory quinolones; (5) macrolides	(1) Only 1.9% the *S. pneumoniae* isolates from adult CAP are resistant to intravenous penicillins in China. The percentage of intermediate strains is only about 9%. Intravenous penicillins are still effective in hospitalized patients infected with penicillin-intermediate *S. pneumoniae* when increasing the dosage (23, 161); (2) When atypical pathogens are suspected, doxycycline or minocycline or respiratory quinolones are preferred. Macrolides can be used in regions with lower resistance rate to mycoplasma
Patients with underlying disease(s) or elderly patients (age ≥ 65 years)	*S. pneumoniae*, *H. influenzae*, *Enterobacteriaceae* such as *K. pneumoniae*, influenza virus, RSV, *M. catarrhalis*, anaerobic bacteria, *Legionella*	(1) Penicillins-β-lactamase-inhibitor combinations; (2) III generation cephalosporins or their enzyme-inhibitor combinations, carbapenems such as cephamycins, oxacephems, ertapenem; (3) monotherapy of the above drugs or in combination with macrolides; (4) respiratory quinolones	(1) *Enterobacteriaceae* infection must be considered in patients with underlying disease(s) and elderly patients. The patients must be further evaluated for the risk of infection with ESBLs-producing *Enterobacteriaceae*; (2) Elderly patients should be monitored for the risk factors of aspiration
Requirement for ICU admission (Intravenous administration is recommended)			
Young adults without underlying disease(s)	*S. pneumoniae*, *S. aureus*, influenza virus, adenovirus, *Legionella*	(1) Penicillins-β-lactamase-inhibitor combinations, III generation cephalosporins, cephamycins, oxacephems, ertapenem combined with macrolides; (2) respiratory quinolones	(1) *S. pneumoniae* is the most common pathogen. The other pathogens such as *S. aureus*, *Legionella*, influenza virus should also be considered (1, 2, 162-166); (2) During influenza seasons, attention must be paid to influenza viral infections. Combination with neuraminidase inhibitors should be considered. Attention should be paid to secondary *S. aureus* infection (167). The agents active against MRSA can be used in combination if necessary

For peer review only - http://bmjopen.bmj.com/site/about/guidelines.xhtml

Patients with underlying disease(s) or elderly patients (age ≥ 65 years)	*S. pneumoniae*, *Legionella*, *Enterobacteriaceae* such as *K. pneumoniae*, *S. aureus*, anaerobic bacteria, influenza virus, RSV	(1) Penicillins-β-lactamase-inhibitor combinations, III generation cephalosporins or in combination with beta-lactamase inhibitors, carbapenems such as ertapenem combined with macrolides; (2) penicillins-β-lactamase-inhibitor combinations, III generation cephalosporins or in combination with beta-lactamase inhibitors, carbapenems such as ertapenem combined with respiratory quinolones	(1) Evaluate the risk of infection with ESBLs-producing *Enterobacteriaceae*; (2) Physicians should be aware of the risk factors for aspiration and antimicrobial coverage of relevant pathogens
CAP with risk factors for *P. aeruginosa* infection and requirement for inpatient treatment or ICU admission (Intravenous administration is recommended)			
Patients with structural lung disease	*P. aeruginosa*, *S. pneumoniae*, *Legionella*, *Enterobacteriaceae* such as *K. pneumoniae*, *S. aureus*, anaerobic bacteria, influenza virus, RSV virus	(1) β-lactams with antipseudomonal activity; (2) quinolones with antipseudomonal activity; (3) β-lactams with antipseudomonal activity combined with quinolones or aminoglycosides with antipseudomonal activity; (4) combination of β-lactams, aminoglycosides and quinolones with antipseudomonal activity	Risk factors include: (1) airway *P. aeruginosa* colonization; (2) repeated doses of antibacterial drugs or glucocorticoids due to chronic airway disease. Combination therapy is recommended for patients with severe CAP or proven antimicrobial resistance

For peer review only - http://bmjopen.bmj.com/site/about/guidelines.xhtml

For peer review only

Appendix Figure 1 Patient screening algorithm for hospitalized CAP

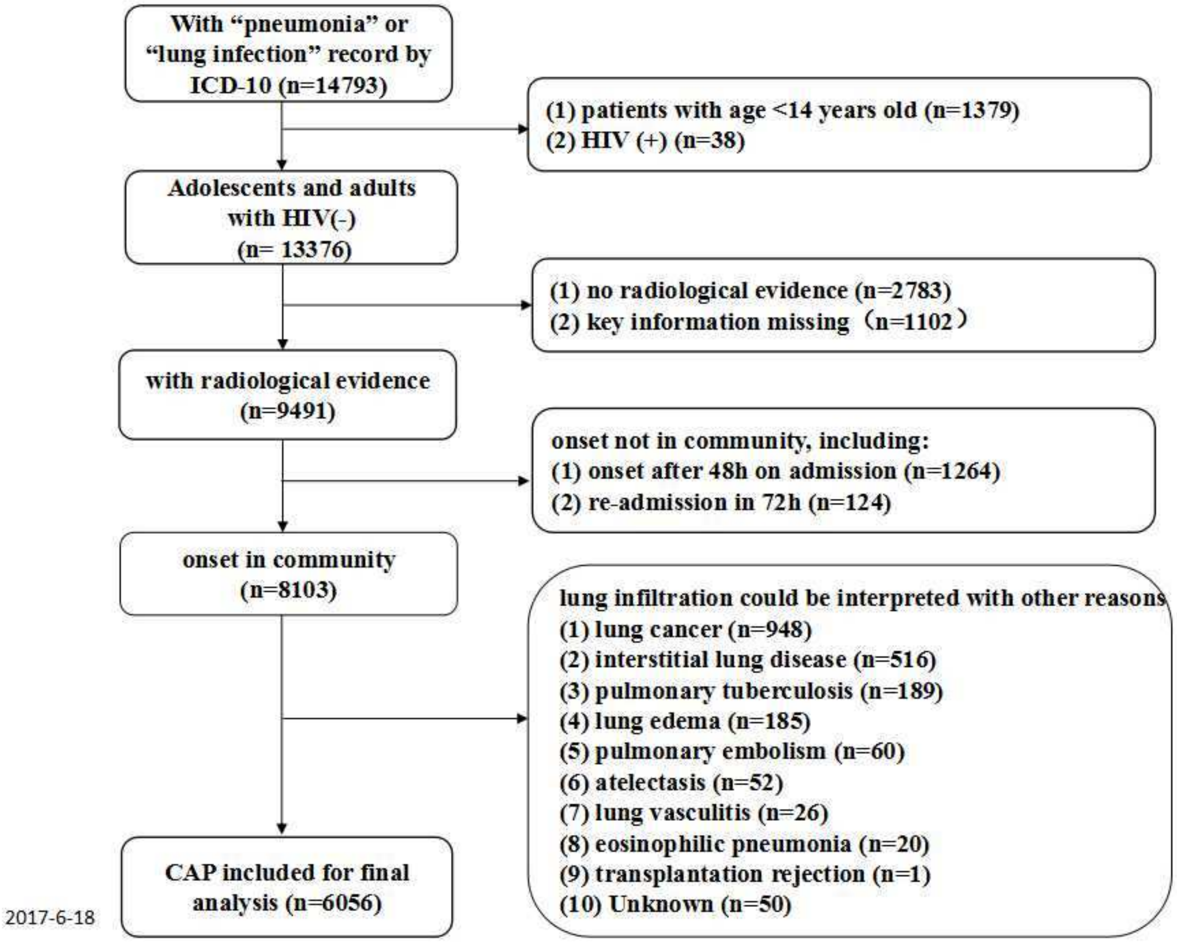

For peer review only

For peer review only - http://bmjopen.bmj.com/site/about/guidelines.xhtml

BMJ Open

Disease characteristics and management of hospitalized adolescents and adults with Community-Acquired Pneumonia in China：a retrospective multicenter survey

Journal:	*BMJ Open*
Manuscript ID	bmjopen-2017-018709.R1
Article Type:	Research
Date Submitted by the Author:	06-Sep-2017
Complete List of Authors:	Chen, Liang; Department of Infectious Diseases and Clinical Microbiology, Beijing Chao-Yang Hospital, Capital Medical University<br>Zhou, Fei; Department of Infectious Diseases and Clinical Microbiology, Beijing Chao-Yang Hospital, Capital Medical University<br>Li, Hui; Department of Pulmonary and Critical Care Medicine, Center for Respiratory Diseases, National Clinical Research Center of Respiratory Diseases; China-Japan Friendship Hospital<br>Xing, Xiqian; Department of Respiratory Medicine, Yan'an Hospital Affiliated to Kunming Medical University, Department of Respiratory Medicine, Yan'an Hospital Affiliated to Kunming Medical University, Kunming City, Yunnan Province<br>Han, Xiudi; Department of Respiratory Medicine, Qingdao Municipal Hospital, Qingdao City, Shandong Province<br>Wang, Yiming; Department of Pulmonary and Critical Care Medicine, Center for Respiratory Diseases, National Clinical Research Center of Respiratory Diseases; China-Japan Friendship Hospital<br>Zhang, Chunxiao; Department of Respiratory Medicine, Beijing Huimin Hospital, Beijing<br>Suo, Lijun; Department of Respiratory Medicine, Linzi District People's Hospital, Zibo City, Shandong Province<br>Wang, Jingxiang; Department of Respiratory Medicine, Beijing Luhe Hospital Affiliated to Capital Medical University, Beijing<br>Yu, Guohua; Department of Pulmonary and Critical Care Medicine, Weifang NO.2 People's Hospital, Weifang City, Shandong Province<br>Wang, Guangqiang; Department of Respiratory Medicine, Qilu Hospital Of Shandong University (Qindao), Qingdao City, Shandong Province<br>Yao, xuexin; Department of Respiratory Medicine, The 2nd Hospital of Beijing Corps, Chinese Armed Police Forces, Beijing<br>Yu, Hongxia; Department of Infectious Disease, Yantai Yuhuangding Hospital, Yantai City, Shandong Province<br>Wang, Lei; Department of Respiratory Medicine, Rizhao Chinese Medical Hospital Affiliated to Shandong Chinese Medical University, RiZhao City, Shandong Province<br>Liu, Meng; Department of Infectious Diseases and Clinical Microbiology, Beijing Chao-Yang Hospital, Capital Medical University, Beijing<br>Xue, Chunxue; Department of Infectious Diseases and Clinical Microbiology, Beijing Chao-Yang Hospital, Capital Medical University, Beijing<br>Liu, Bo; Department of Respiratory Medicine, Linzi District People's Hospital, Zibo City, Shandong Province

	Zhu, Xiaoli; Occupational Medicine and Toxicology Department, Beijing Chao-Yang Hospital, Capital Medical University, Beijing<br>Li, Yanli; Department of Infectious Diseases and Clinical Microbiology, Beijing Chao-Yang Hospital, Capital Medical University, Beijing<br>Xiao, Ying; Department of Infectious Diseases and Clinical Microbiology, Beijing Chao-Yang Hospital, Capital Medical University, Beijing<br>Cui, Xiaojing; Department of Pulmonary and Critical Care Medicine, Center for Respiratory Diseases, National Clinical Research Center of Respiratory Diseases; China-Japan Friendship Hospital<br>Li, Lijuan; Department of Pulmonary and Critical Care Medicine, Center for Respiratory Diseases, National Clinical Research Center of Respiratory Diseases; China-Japan Friendship Hospital<br>Uyeki, Timothy; Influenza Division, National Center for Immunization and Respiratory Diseases, Centers for Disease Control and Prevention-Atlanta, Georgia, USA<br>Wang, Chen; Department of Pulmonary and Critical Care Medicine, Center for Respiratory Diseases, National Clinical Research Center of Respiratory Diseases; China-Japan Friendship Hospital<br>Cao, Bin; Department of Pulmonary and Critical Care Medicine, Center for Respiratory Diseases, National Clinical Research Center of Respiratory Diseases; China-Japan Friendship Hospital
**Primary Subject Heading**:	Infectious diseases
Secondary Subject Heading:	Medical management
Keywords:	disease characteristics, management, Community-Acquired Pneumonia, China

SCHOLARONE™
Manuscripts

For peer review only - http://bmjopen.bmj.com/site/about/guidelines.xhtml

Disease characteristics and management of hospitalized adolescents and adults with Community-Acquired Pneumonia in China：a retrospective multicenter survey

Liang Chen[1,2], Fei Zhou[1], Hui Li[3], Xiqian Xing[4], Xiudi Han[5],Yimin Wang[3], Chunxiao Zhang[6], Lijun Suo[8], Jinxiang Wang[9], Guohua Yu[11], Guangqiang Wang[12], Xuexin Yao[13], Hongxia Yu[14], Lei Wang[7], Meng Liu[1], Chunxue Xue[1], Bo Liu[8], Xiaoli Zhu[10],Yanli Li[1]， Ying Xiao[1], Xiaojing Cui[3], Lijuan Li[3], Timothy M. Uyeki[15], Chen Wang[3,16]， Bin Cao[3,16] for CAP-China network

1. Department of Infectious Diseases and Clinical Microbiology, Beijing Chao-Yang Hospital, Capital Medical University, Beijing

2. Department of Infectious Disease, 4th Medical College of Peking University, Beijing Jishuitan Hospital, Beijing

3. Department of Pulmonary and Critical Care Medicine, Center for Respiratory Diseases, National Clinical Research Center of Respiratory Diseases; China-Japan Friendship Hospital

4. Department of Respiratory Medicine, Yan'an Hospital Affiliated to Kunming Medical University, Kunming City, Yunnan Province

5. Department of Respiratory Medicine, Qingdao Municipal Hospital, Qingdao City，Shandong Province

6. Department of Respiratory Medicine, Beijing Huimin Hospital, Beijing

7. Department of Respiratory Medicine, Rizhao Chinese Medical Hospital Affiliated to Shandong Chinese Medical University, RiZhao City, Shandong Province

For peer review only

8. Department of Respiratory Medicine, Linzi District People's Hospital, Zibo City, Shandong Province

9. Department of Respiratory Medicine, Beijing Luhe Hospital Affiliated to Capital Medical University, Beijing

10. Occupational Medicine and Toxicology Department, Beijing Chao-Yang Hospital, Capital Medical University, Beijing

11. Department of Pulmonary and Critical Care Medicine, Weifang NO.2 People's Hospital, Weifang City, Shandong Province

12. Department of Respiratory Medicine, Qilu Hospital Of Shandong University (Qindao), Qingdao City, Shandong Province

13. Department of Respiratory Medicine, The 2nd Hospital of Beijing Corps, Chinese Armed Police Forces, Beijing

14. Department of Infectious Disease, YantaiYuhuangding Hospital, Yantai City, Shandong Province

15. Influenza Division, National Center for Immunization and Respiratory Diseases, Centers for Disease Control and Prevention-Atlanta, Georgia, USA

16. Department of Pulmonary Medicine; Capital Medical University, Beijing;

Corresponding Author: Dr. Bin Cao, MD. Center for Respiratory Diseases，Department of Pulmonary and Critical Care Medicine，China-Japan Friendship Hospital；National Clinical Research Center of Respiratory Diseases；Capital Medical University. caobin1999@gmail.com

For peer review only - http://bmjopen.bmj.com/site/about/guidelines.xhtml

Abstract

Objectives To describe the clinical characteristics and management of patients hospitalized with CAP in China.

Design This was a multicenter, retrospective, observational study.

Setting 13 teaching hospitals in northern, central and southern China from 1 January 2014 to 31 December 2014

Participants Information on hospitalized patients aged ≥14 years with radiographically-confirmed pneumonia with illness onset in the community was collected using standard case report forms.

Primary and secondary outcome measures Resource use for CAP management.

Results Of 14,793 patients screened, 5828 with radiographically-confirmed CAP were included in the final analysis. Low mortality risk patients with a CURB-65 score 0-1 and PSI risk class Ⅰ-Ⅱ accounted for 81.2% (4434/5594) and 56.4% (2034/3609) of CAP patients respectively. 21.7% (1111/5130) patients had already achieved clinical stability on admission. 40.9% (1575/3852) patients without pseudomonal infection risk factors received antimicrobial over-treatment regimens. The median length of stay in hospital was 11 days. The median duration between clinical stability to discharge was 5.0 days with 30-day mortality of 4.2%.

Conclusions These data demonstrated overuse of health resources in CAP management, indicating that there is the potential for improvement and substantial savings to health-care systems in China.

Strengths and limitations of this study

For peer review only - http://bmjopen.bmj.com/site/about/guidelines.xhtml

- This is the largest multi-center study to investigate demographic characteristics, severity and microbiological testing, empirical antimicrobial treatment, duration of hospitalization and 30-day mortality among adults and adolescents hospitalized with CAP in mainland China, including adolescents and adults of all ages admitted to general hospital wards or ICUs from the participating centers, patients who were critically ill and aged >90 years.
- The participating hospital sites are teaching hospitals in seven cities in three provinces, and may not be representative of CAP in smaller, rural hospitals.
- The majority of patients are adult CAP patients, our findings do not apply to children hospitalized with CAP.

Background

Community acquired pneumonia (CAP) is one of the most common infectious syndromes and is a leading cause of death worldwide.[1 2] In Europe, the reported rate of CAP ranges from 1.6 to 9 cases per 1,000 in the general adult population per year.[3 4 5] Despite advances in medical technology and global economic development, CAP-associated mortality remains high (e.g., 20.9/100,000 in the United States and 12.7/100,000 in Canada).[2 6] Patients hospitalized in intensive care units for CAP have mortality in excess of 20% for immunocompetent patients and closer to 30% for those immunocompromised.[7] In Japan and Korea, the 30-day mortality of patients hospitalized with CAP is about 4-6%.[8 9]

Although mainland China has nearly 19% of the world’s population, there are limited data on CAP management and disease burden in China during the last ten

For peer review only - http://bmjopen.bmj.com/site/about/guidelines.xhtml

years. According to a household interview survey published in the China Health and Family Planning Statistical Yearbook (2013), the two-week prevalence of pneumonia in China was estimated to be 11/1,000, and the direct cost due to bacterial pneumonia was about 320 million RMB (approximately $46.4 million).[10] In 2015, CAP-China, a multicenter clinical network, was founded with the support of National Key Technology Support Program from Ministry of Science and Technology (2015BAI12B11) to provide data on CAP for clinical researchers and healthcare policy makers in China.

A multicenter retrospective study of all hospitalized CAP patients from 13 centers in northern, central and southern China among CAP-China members was implemented in 2015 (Clinicaltrial Registration No.NCT02489578). To our knowledge, this is the largest multi-center study to investigate demographic characteristics, severity and microbiological testing, empirical antimicrobial treatment, duration of hospitalization and 30-day mortality among adults and adolescents hospitalized with CAP in mainland China.

Methods

Study Design and Population

Data were collected from 13 hospitals in Northern (Beijing), Central (Yantai, Qindao, Weifang, Zibo, Rizhao cities in Shandong Province) and Southern (Kunming City in Yunan Province) China. A listing of participating centers can be found in Appendix 1. All patients admitted to the 13 hospitals during 1 January 2014 through 31 December 2014 with the relevant disease codes of pneumonia or pulmonary infection in the

For peer review only - http://bmjopen.bmj.com/site/about/guidelines.xhtml

World Health Organization International Classification of Diseases 10th revision (ICD-10, Appendix 2) were eligible. Data on all eligible patients identified in screening were retrieved from the Hospital Information System (HIS) in each center. Trained physicians reviewed the medical case history and collected data on 786 variables for each patient. Chest radiographs and computerized tomography (CT) scans for each patient were reviewed by pulmonary physicians and radiologists in each center. Two-leveled review process was performed for data collection and entry.

The CAP case definition includes (1) illness onset in the community(defined as community acquired infection among those who have not been hospitalized during recent 28 days)[11]; (2) chest radiograph or CT scan showing infiltrate or interstitial changes, with or without pleural effusion; (3) any one of pneumonia clinical manifestations: (a) recent cough, sputum or aggravation of respiratory symptoms, the emergence of purulent sputum, with or without chest pain; (b) fever (defined as axillary temperature ⩾37.3°C)[12] or hypothermia (axillary temperature <36 °C); (c) signs of pulmonary consolidation and (or) moist crackles; or (d) WBC $>10\times10^9$/L, or $<4\times10^9$/L, with or without neutrophil predominance.

Patients were excluded if (1) age <14 years; (2) pneumonia onset ⩾48 hours after admission; (3) lung infiltrate or interstitial changes which were interpreted as lung cancer, pulmonary tuberculosis, non-infectious interstitial lung diseases, pulmonary edema, atelectasis, pulmonary embolism, pulmonary eosinophil infiltrate, pulmonary vasculitis; (4) immunocompromised status; (5) re-admission within 72 hours after discharge.

For peer review only - http://bmjopen.bmj.com/site/about/guidelines.xhtml

The study design was approved by the Ethics committee of China-Japan Friendship Hospital (No.2015-86). Given the retrospective nature of the study, the Ethics committee determined that informed consent was not necessary.

Quality control of the study

Key investigators, including clinicians, statisticians, microbiologists and radiologists worked together to draft the protocol and created a single formatted case report form (CRF) that was utilized by all centers. Before study initiation, all investigators from the thirteen centers received training on the protocol, screening process, definition of underlying diseases and formatted CRF（Appendix file 3). After data were collected, the CRF was reviewed by a trained researcher to ensure its completeness and data quality. A second review was performed independently by a trained team of physicians in each center before being entering in duplicate into a computerized database.

Data Collection:

A total of 786 variables were included in the formatted CRF, including:

(1) Demographic data: age, gender, ID number, source of admission, types of medical insurance;

(2) Underlying diseases: chronic lung, heart, renal and liver diseases, diabetes, hypertension, solid organ cancers. Definition of underlying diseases is listed in Appendix file 4.

(3) Factors for acquisition or prevention of CAP: pregnancy, postpartum within six months, current smoking history, excessive drinking, exposure to day care center

For peer review only

children, bed-ridden longer than two months, chronic receipt of corticosteroids (dosage equivalent prednisolone ≥10mg/d for more than 30 days), statin use, *S. pneumonia* or Influenza vaccination within one year.

(4) Clinical manifestations, clinical signs: recorded on the day of admission, on the 4th hospital day, change of antibiotics within 14 days of admission, and the day of discharge or death. Laboratory and radiological findings were also recorded if such tests were repeated by attending physicians. Pneumonia disease severity scores (PSI /CURB-65) were also recorded.

(5) Microbiological examination: Gram stain and culture of sputum within 48 hours, blood culture within 48 hours, BALF and pleural fluid culture within one week after admission, serum antibody (including IgM and IgG) for atypical pathogens (*Mycoplasma pneumoniae, Chlamydia pneumoniae, Legionella pneumophila*). Urinary antigen testing was performed for *Streptococcus pneumonia* and *Legionella spp.* Real-time PCR testing was done for respiratory virus and atypical pathogens with sputum and BALF. Nasopharyngeal (NP) swab was used for antigen testing for Influenza A and Influenza B. Aspirate was not routinely used for antigen testing.

(6) Antimicrobial treatment before admission and change of antimicrobials during hospitalization. Use of corticosteroids, vasopressors, mechanical ventilation, Continuous renal replacement therapy (CRRT) and extracorporeal membrane oxygenation (ECMO) were also recorded.

(7) Clinical stability was defined as satisfying all of the following: axillary temperature ≤37.8 °C more than 24 hours without use of antipyretic medications;

For peer review only - http://bmjopen.bmj.com/site/about/guidelines.xhtml

resting heart rate ≤100 beats/min; respiratory rate ≤24 breaths/ minute; systolic blood pressure ≥90mmHg; SpO2 ≥ 90% on room air; ability to maintain oral intake; normal mental status.[13]

(8) Over-treatment was defined as: i) use of antipseudomonal β-lactams or β-lactams+ fluoquinolones in hospitalized (not in ICU) patients without risk factors for pseudomonal infection; ii) use of β-lactams (antipseudomonal or not)+ fluoquinolones in ICU patients aged< 65yr without risk factors for pseudomonas infection；iii) use of anti-MRSA drugs in hospitalized (not in ICU) patients.[14]

(9) Risk factors for pseudomonal infection was defined as chronic airway disease (bronchiectasis and COPD) and at least one risk factor for HCAP as defined by the 2005 IDSA/ATS adult CAP guidelines.[14 15 16 17 18]

(10) Empirical antimicrobial regimens recommended by Chinese CAP guidelines were showed in Appendix 5.

Microbiology testing

The conditions that a pathogen was defined as the definite or probable etiology based on were showed in Appendix 6.

Statistical analysis

No formal sample size calculations were performed because of the retrospective descriptive study design. All data were analyzed by descriptive statistics with SPSS19. Measurement data were tested for normality by Kolmogorov-Smirnov. Measurement data of normal distribution was reported as mean ± standard deviation. Measurement data of non-normal distribution was reported as median. The χ2 test statistics were

For peer review only - http://bmjopen.bmj.com/site/about/guidelines.xhtml

used for 30-day mortality subgroup analysis. A P-value of <0.05 was considered statistically significant.

Results

Screening Process

A total of 14,793 patients were screened to meet the inclusion and exclusion criteria for CAP and 5828 patients were included in the final analysis (Appendix Figure 1).

Epidemiological characteristics

The proportions of male and female patients were similar. The median age was 65 years, range 14-103 years. Prevalent co-morbidities included hypertension (35.2%), coronary heart disease (20.0%), diabetes (15.7%), cerebrovascular diseases (15.3%) and COPD（13.7%）. 14.9% of CAP patients had at least one healthcare associated pneumonia (HCAP) risk factor (according to IDSA/ATS HAP/HCAP guideline published in 2005[15]). 45.7% patients received antibiotics before admission.

A substantial proportion of admitted patients had relatively mild disease as indicated by the following: i) CURB-65 score[19] 0-1 accounted for 81.2%, ii) PSI risk class[20]Ⅰ~Ⅱ accounted for 56.3%; iii) Shorr Score[21] 0~1 accounts for 99.6%; and iv) Aliberti Score[22] low riskgroup in 89.7%; v) only 12.0% (261/2172) patients had procalcitonin (PCT) more than 2 ng/ml; vi) as many as 65.7% (3741/5698) patients had normal peripheral leukocyte counts (4,000-10,000/ul). Most importantly, 21.7% patients had met criteria for clinical stability at hospital admission.[13] (Table 1-2)

For peer review only

For peer review only - http://bmjopen.bmj.com/site/about/guidelines.xhtml

Table 1: Demographic characteristics and underlying diseases

Items	Cases (%)
Male	3117 (53.5)
Age (years,median, IQR)	65 (53-78)
14~64	2802 (48.1)
65~74	1081 (18.5)
75~89	1760 (30.2)
≥90	185 (3.2)
Source of admission（n=5823）	
From Out-patient Department	4183 (71.8)
From Emergency Room	1588 (27.3)
Transfer from other hospital	52 (0.9)
Days from illness onset to admission (n=5826, median, IQR)	6.0 (3.0-14.0)
Patients who received antibiotics before admission	2664 (45.7）
β-lactams	1015 (38.1)
Fluoquinolones	586 (22.0)
Macrolides	170 (6.4)
β-lactams+ fluoquinolones	413 (15.5)
β-lactams+ macrolides	201 (7.5)
Others	279 (10.5)
Systemic glucocorticosteroids use before admission	250 (4.3)
Underlying Diseases	4219 (72.4)
Hypertension	2053 (35.2)
Coronary Heart Disease	1163 (20.0)
Diabetes	913 (15.7)
Cerebrovascular Diseases	890 (15.3)
COPD	801 (13.7)
Bronchiectasis	629 (10.8)
Asthma	339 (5.8)
Malignant solid tumors	254 (4.4)
Congestive Heart Failure	202 (3.5)
Chronic renal diseases	201 (3.4)
Connective Tissue Diseases	110 (1.9)
Chronic Hepatic Diseases	90 (1.5)

For peer review only

For peer review only - http://bmjopen.bmj.com/site/about/guidelines.xhtml

Smoking status	
Current smokers	1009 (17.3)
Ex-smokers	590 (10.1)
Alcoholism	407 (7.0)
Risk factors for aspiration*	377 (6.5)
History of CAP within one year	368 (6.3)
History of vaccination	
Influenza vaccine within 1 year	12 (0.2)
Streptococcus pneumoniae vaccine within 5 years	8 (0.1)
Risk factors for HCAP according to IDSA/ATS criteria	868 (14.9)
Hospitalized in an acute care hospital for two or more days within 90 days	404 (6.9)
Received recent intravenous antibiotic therapy, chemotherapy, or wound care within the past 30 days	656 (11.3)
Attended a hospital or hemodialysis clinic	36 (0.6)
Residence in a nursing home or long-term care facility	19 (0.3)
CURB-65 score (n=5594)	
0	2343 (41.9)
1	2199 (39.3)
2	884 (15.8)
3	147 (2.6)
4	20 (0.4)
5	1 (0.0)
PSI risk class (n=3609)	
I	1130 (31.3)
II	904 (25.0)
III	748 (20.7)
IV	646 (17.9)
V	181 (5.0)
Shorr Score (n=5650)	
0	5084 (90.0)
1	541 (9.6)
2	23 (0.4)
3	2 (0.0)
4	0 (0.0)

For peer review only

Aliberti Score（n=5828)	
Low risk group	5226 (89.7)
High risk group	602 (10.3)
Clinical stability on admission § (n=5130)	1111 (21.7)

COPD: chronic obstructive pulmonary disease; HCAP: healthcare associated pneumonia; IDSA/ATS: Infectious Diseases Society America/American Thoracic Society. PSI: pneumonia severity index. *Risk factors for aspiration included choking, drowning, nasal,pseudobulbar palsy, dementia, coma, poisoning, Parkinson's disease.

§ Clinical stability was defined as satisfying the following at the same time: axillary temperature ≤37.8 °C more than 24 hours; heart rate ≤100 beats/min in resting state; breathing rate ≤24 breaths/minute; systolic blood pressure ≥90mmHg; SpO2 ≥90% on room air; ability to maintain oral intake; normal mental status.

Table 2: Clinical and radiological features on admission

**Items**	**Cases（%）**
Axillary Temperature≥38℃ (n=5826)	2783 (47.8)
Axillary Temperature<36℃(n=5793)	44 (0.8)
Cough	5192 (89.1)
Sputum	4751 (81.5)
Shortness of breath	2116 (36.3)
Chest pain	709 (12.2)
Decrease of consciousness	294 (5.0)
Chest signs	
Moist rales	2919 (50.1)
Dry rales	1387 (23.8)
Edema of lower limbs	592 (10.2)
Cyanosis	547 (9.4)
SBP<90 mmHg	45 (0.8)
Radiology	
Infiltrate more than two lobes	3776 (64.8)
Plural effusion	1205 (20.7)
Cavitation	228 (3.9)
WBC (mm^{-3}, n=5698)	

For peer review only
For peer review only - http://bmjopen.bmj.com/site/about/guidelines.xhtml

>10,000	1626 (28.5)
<4,000	331 (5.8)
4,000~10,000	3741 (65.7)
BUN >7.0 mmol·L^{-1} (n=5601)	1166 (20.8)
PH <7.30 (n=3330)	87 (2.6)
PaO_2/FiO_2<300 mmHg (n=3327)	1196 (35.9)
PCT（ng·ml^{-1}, n=2172)	
PCT≤0.25	1307 (60.2)
0.25<PCT<1	479 (22.1)
1≤PCT<2	125 (5.8)
PCT≥2	261 (12.0)

SBP: systolic blood pressure; WBC: white blood cell count; BUN: blood urea nitrogen; Scr: serum creatinine; PH: potential of hydrogen; PaO_2/FiO_2: arterial pressure of oxygen/fraction of inspiration oxygen; PCT:procalcitonin.

Clinical and radiological features

Clinical and radiological features on admission are shown in Table 2. Cough, sputum, shortness of breath and fever were the most common. 64.8% patients had multi-lobar infiltrates and 20.7% of patients had pleural effusion.

Microbiological testing

75.0% patients had some types of microbiologic testing. 68.9% of patients had a sputum culture obtained within 48 hours of admission, although only 18.5% of patients were able to produce a sputum culture of acceptable quality. The proportion of patients with blood culture, BALF culture, and pleural effusion culture were 10.3%, 9.1% and 1.9% respectively. Only 0.8% of patients had a urinary antigen test sent to evaluate for *Legionella pneumophila*, and 2.6% had urinary antigen testing for *Streptococcus pneumoniae*. (Table 3)

For peer review only - http://bmjopen.bmj.com/site/about/guidelines.xhtml

Table 3: Microbiological examination for CAP

**Items**	**Cases (%)**
Any Microbiological examination	4371 (75.0)
Microbiological examination for bacterial	4015 (68.9)
Microbiological examination for atypical etiology	1983 (34.0)
Microbiological examination for virus	2014 (34.6)
Bacterial or fungal Culture	4015 (68.9)
Qualified sputum culture*	1078 (18.5)
Blood culture **	602 (10.3)
BALF culture*$^{+}$	532 (9.1)
Pleural effusion culture**	108 (1.9)
**Antibody-Based Assays on acute serum**	
Mycoplasma pneumoniae	IgM: 1821 (31.2)<br>IgG: 794 (13.6)
Chlamydia pneumoniae	IgM: 1294 (22.2)<br>IgG: 220 (3.8)
Legionella pneumoniae	IgM: 645 (11.1)<br>IgG: 227 (3.9)
Adenovirus	IgM: 644 (11.1)<br>IgG: 0 (0.0)
Respiratory syncytial virus	IgM: 643 (11.0)<br>IgG: 0 (0.0)
Influenza A virus	IgM: 643 (11.0)<br>IgG: 0 (0.0)
Influenza B virus	IgM: 640 (11.0)<br>IgG: 0 (0.0)
Parainfluenza virus	IgM: 643 (11.0)<br>IgG: 0 (0.0)
**Nucleic Acid-Based Molecular Diagnostics**	
From sputum	297(5.1)
Time Interval¶(days, median, IQR)	9.0 (6.0-16.0)
From BALF^{+}	19 (0.3)

For peer review only - http://bmjopen.bmj.com/site/about/guidelines.xhtml

Time Interval¶(days, median, IQR)	13.0 (9.0-24.0)
Mycoplasma pneumoniae	270 (4.6)
Chlamydia spp	270 (4.6)
Legionella spp	270 (4.6)
Influenza A virus	270 (4.6)
Influenza B virus	270 (4.6)
Other respiratory virus#	270 (4.6)
**Urinary Antigen test**	
Streptococcus pneumoniae	150 (2.6)
Legionella spp	47 (0.8)
**Nasopharyngeal swab antigen testing**	
Influenza A virus	41 (0.7)
Influenza B virus	21 (0.4)

*： within 48hr after admission

**:within one week after admission

¶: days from illness onset to testings

#parainfluenza virus (PIV) types 1, 2, 3 and 4, rhinovirus (HRV), enterovirus (EV), coronovirus (hCoV) types 229E, NL63, OC43 and HKU1, parapneumovirus (hMPV), and adenovirus (AdV), bocavirus

+**BALF:** bronchoalveolar lavage fluid

Of all patients, serological testing for antibodies to *Mycoplasma pneumoniae* was only performed on a single serum specimen for IgM (31.2%) and IgG antibodies (13.6%). Similarly, serological testing on a single serum specimen was done for *Chlamydia pneumoniae* IgM antibody in 22.2% of patients and for IgM antibodies to *Legionella pneumophila* and respiratory viruses in 11.1%. No convalescent serum specimens were collected for serological testing for any pathogens, limiting interpretation of serology results for a single serum specimen.

For peer review only - http://bmjopen.bmj.com/site/about/guidelines.xhtml

A definite or probable pathogen was identified only in 12.7% of patients (738/5828): only bacteria in 87.1% (643/738), only atypical pathogens in 0.9% (7/738), only viruses in 8.5% (63/738), bacteria and viruses in 2.7% (20/738), viruses and atypical pathogens in 0.7% (5/738). The most common five pathogens identified were *Pseudomonas aeruginosa* 26.7% (197/738), *Klebsiella pneumonia* 17.6% (130/738), *Escherichia.coli* 8.9%(66/738), *Acinetobacter* 8.4% (62/738) and influenza A virus 7.3% (54/738). (Appendix 7)

Empiric antimicrobial regimens

β-lactams (received by 72.7% of patients) and fluoquinolones (received by 42.2%) were the most common classes of antibiotics that were administered empirically. In patients (not in ICU) without pseudomonal infection risk factors, 27.8% (1070/3852) patients received empiric antibiotic regimens including antipseudomonal β-lactams, and 12.1% (468/3852) patients received β-lactams + fluoquinolones; 0.4% (16/3852) patients aged <65 years and not in ICU received β-lactams (antipseudomonal or not) + fluoquinolones combined regimens. Overall, 40.9% (1575/3852) patients without pseudomonal infection risk factors received antimicrobial over-treatment regimens.(Table 4)

Table 4: Empirical antimicrobial regimen for CAP patients (n=5716)*

**Empirical antimicrobials (%)**	**Without risk factors for *P. seudomonas* infection （n=3852）**				**With risk factors for *P. seudomonas* infection (n=1864)**
	age<65yr and not in ICU (n=1881)	age<65yr and in ICU (n=79)	age≥65yr and not in ICU (n=1742)	age≥65yr and in ICU (n=150)	

For peer review only - http://bmjopen.bmj.com/site/about/guidelines.xhtml

β-lactams (antipseudomonal)	178 (4.6)[#]	21 (0. 5)	407 (10.6)[#]	58 (1.5)	541 (29.0)
β-lactams	331 (8.6)	9 (0.2)	482 (12.5)	20 (0.5)	345 (18.5)
Fluoquinolones	502 (13.0)	10 (0.3)	273 (7.1)	6 (0.2)	252 (13.5)
Macrolides	20 (0.5)	0 (0.0)	17 (0.4)	0 (0.0)	10 (0.5)
β-lactams (antipseudomonal) + fluoquinolones	201 (5.2)[#]	13 (0.3)[#]	189 (4.9)[#]	30 (0.8)	238 (12.8)
β-lactams+ fluoquinolones	302 (7.8)[#]	3 (0.1)[#]	166 (4.3)[#]	9 (0.2)	177 (9.5)
β-lactams+ macrolides	160 (4.2)	2 (0.1)	64 (1.7)	2 (0. 1)	55 (3.0)
β-lactams (antipseudomonal) + macrolides	50 (1.3)[#]	0 (0.0)	45 (1.2)[#]	2 (0.1)	58 (3.1)
Fluoquinolones + macrolides	24 (0.6)	0 (0.0)	11 (0.3)	0 (0.0)	6 (0.3)
anti-MRSA drugs	9 (0.2)[#]	8 (0.2)	12 (0.3)[#]	6 (0.2)	29 (1.6)
Others	104 (2.7)	13 (0.3)	76 (2.0)	17 (0.4)	153 (8.2)

*: data on empirical antimicrobial regimens in 112 patients were missing.

[#]Overtreatment was defined as: i) use of antipseudomonal β-lactams or β-lactams+ fluoquinolones in hospitalized (not in ICU) patients without risk factors for pseudomonal infection; i i) use ofβ-lactams(antipseudomonal or not)+ fluoquinolones in ICU patients aged< 65yr without risk factors for pseudomonas infection；iii) use of anti-MRSA drugs in hospitalized (not in ICU) patients. [14]

- Risk factors for *P. seudomonas* infectionwas defined as chronic airway disease (bronchiectasis or COPD) and HCAP according to IDSA/ATS criteria.[15]

Clinical outcomes

Clinical outcomes are shown in Table 5. Overall, 6.3% of patients were admitted to an ICU, and 2.7% required invasive mechanical ventilation. Vasopressors were administered to 3.4% of patients, and 26.4% received corticosteroids during the hospitalization. The 30-day mortality was 4.2%. The median duration of hospitalization was 11 days. The median duration from admission to clinical stability

For peer review only - http://bmjopen.bmj.com/site/about/guidelines.xhtml

was 4 days, and from clinical stability to discharge was 5 days. The median duration of ICU hospitalization was 8 days. The top five causes of death were severe pneumonia/multi-organ dysfunction syndrome (MODS) 69.1% (170/246), cardiac failure2.8% (7/246), acute myocardial infarction 2.0% (5/246), stroke 1.6% (4/246) and gastrointestinal hemorrhage 1.6% (4/246).

Table 5: Supportive treatment and clinical outcomes of patients with CAP

Items	Cases（%）
ICU admission	367 (6.3)
Mechanical ventilation	
Non-invasive ventilation	286 (4.9)
Invasive ventilation in ICU	123 (2.1)
Invasive ventilation not in ICU	33 (0.6)
Vasopressor use	197 (3.4)
CRRT	16 (0.3)
ECMO	3 (0.1)
Systemic glucocorticosteroids use after diagnosis of CAP	1540 (26.4)
ICU patients who received systemicglucocorticoids	154 (2.6)
Patients on invasive mechanical ventilation who received systemic glucocorticoids	75 (1.3)
Patients on non-invasive mechanical ventilation who received systemic glucocorticoids	158 (2.7)
30-day mortality	246 (4.2)
Length of stay in Hospital (days, median,IQR)	11.0 (5.0-24.0)
Days between admission-clinical stability (median, n=5130,IQR)	4.0 (1.0-10.0)
Days between clinical stability-discharge (median, n=5130,IQR)	5.0 (1.0-9.0)
Length of stay in ICU (days, median, n=350,IQR)	8.0 (4.0-16.0)
Treatment failure within 14 days	427 (7.3)
Needs non-invasive ventilation	169 (2.9)
Needs invasive ventilation	145 (2.5)

For peer review only - http://bmjopen.bmj.com/site/about/guidelines.xhtml

Needs vasopressors	130 (2.2)
Death	147 (2.5)
Direct causes of death	
Severe pneumonia/MODS	170 (69.1)
Heart failure	7 (2.8)
Acute myocardial infarction	5 (2.0)
Stroke	4 (1.6)
Hemorrhage of digestive tract	4 (1.6)
Acute renal failure	2 (0.8)
Arhythmia	2 (0.8)
Accident aspiration	1 (0.4)
Others	51 (20.7)

ICU: intensive care unit; CRRT: continuous renal replacement therapy; ECMO: extracorporeal membrane oxygenation; MODS: multiple organ dysfunction syndrome; DIC: disseminated intravascular coagulation.

Appendix8 shows the results of sub-group analysis of 30-day mortality. Fatality increased with age. Mortality was similar between male and female patients (4.9% vs 3.5%). Mortality in patients admitted to an ICU was 15.3%.

Discussion

This study represents the largest, multicenter, retrospective cohort study on the etiologies and outcomes in adolescents and adults with CAP in China. In this study, we found that admission of patients with low mortality risk, overuse of antibiotics and incorrect serological testing for *Mycoplasma pneumoniae*, *Chlamydia pneumoniae, Legionella pneumophila* and respiratory viruses, were the main challenges of CAP management.

We identified four major categories of overuse of health care resources in CAP management in China:

For peer review only - http://bmjopen.bmj.com/site/about/guidelines.xhtml

(1) A large number of low-risk patients were admitted to the hospitals. Guidelines for CAP management in China and the U.S. recommend that decisions for hospitalization should be based on illness severity.[14 23] It was estimated that over $8 billion dollars are spent in CAP treatment every year in the U.S, and the cost for inpatient CAP management is 25-30 times more than for outpatient CAP management.[24 25 26] Therefore, admission of low mortality risk CAP patients results in major unnecessary cost expenditures. Moreover, outpatients usually return to their baseline activity levels much sooner than inpatients, and enjoyed a higher quality of life.[27 28] Finally, hospitalization is associated with the risk of nosocomial infections, potentially caused by high virulent and multidrug-resistant organisms.[29] Admission of low-risk CAP patients was also observed in a recent large U.S. study,[11] so it may not be unique to China. However, there are many other factors that play an important role in deciding the need for hospitalization such as comorbidities, lack of available family support, older age, mental illness and drug abuse, etc.[30 31]

(2) Length of stay in hospital was unnecessarily long. CAP guidelines recommended that patients should be discharged as soon as they achieve clinical stability and have no other active medical problems. Keeping patients in hospital and observing them while receiving oral antibiotic therapy, or waiting for normalization of all clinical parameters are not indicated and are associated with increased costs and potentially with in-hospital adverse events.[13 29 30] We observed that CAP patients were discharged a median of 5 days after achieving clinical stability, and 22% met clinical stability criteria at admission. Given the median LOS of 11 days for all CAP patients,

For peer review only - http://bmjopen.bmj.com/site/about/guidelines.xhtml

discharging CAP patients once they achieved clinical stability would lead to cost-savings of approximately half of the total hospitalization expenses. Similarly, the length of stay in hospital may be influenced by other social factors.

(3) 40.9% patients without risk factors for *Pseudomonal* infection received over-treatment with empiric antimicrobial regimens. Antipseudomonal β-lactams (28.2%) or β-lactams + quinolones (12.2%) were the most common empiric regimens for over-treatment. This may be due to overestimation of illness severity, clinician unfamiliarity with CAP guidelines, or lack of microbiologic diagnostic testing. Moreover, we found quinolones use in more than 40% of CAP patients. The U.S. Food and Drug Administration (FDA) has released warnings of potential adverse effects of fluoroquinolones, such as Q-T prolongation, tendon injury, psychiatric disorder, etc.[32 33 34] As second-line anti-tuberculosis drugs, fluoroquinolones can also affect the diagnosis of tuberculosis and induce drug-resistance.[35 36]

(4) Incorrect serological testing was performed. We observed that many patients had an acute serum specimen collected for IgG serology testing for atypical bacteria and respiratory viruses without a convalescent serum specimen obtained for paired serological testing. Furthermore, many patients had testing for IgM antibodies for a variety of respiratory pathogens, but elevation of IgM antibodies with a low-normal IgG titer is uncommon during acute illness.[37 38 39] Paired serology for virus and atypical pathogens is recommended for epidemiological purpose. A follow-up convalescent serum specimen to document changes in IgG and IgM antibody levels is generally required for diagnosis.[40 41] Thus, the value of antibody testing on a single acute serum

For peer review only - http://bmjopen.bmj.com/site/about/guidelines.xhtml

specimen to determine the etiology of CAP is questionable. The costs of more frequent use of PCR testing on lower respiratory specimens may be partially offset by not performing serological testing in CAP patients.

The strengths of this study, in contrast to some past epidemiological investigations,[42] included data on bacterial isolates obtained in current clinical practice, microbiologic testing ordered, and antimicrobials administered, according to Chinese standards-of-care, and the study population included adolescents and adults of all ages admitted to general hospital wards or ICUs from the participating centers to reduce selection bias. We also included patients who were critically ill, aged >90 years and with risk factors for HCAP.

This study had several limitations. First, given the retrospective study design, it is possible that selection bias was present and the study population may not have been representative of all CAP patients admitted to the 13 participating sites. Secondly, the participating hospital sites were teaching hospitals in seven cities in three provinces, and were not selected to be representative of CAP hospital management in China, especially in smaller, rural hospitals. Third, this study reports on CAP management during 2014; analysis of multiple years of data can allow assessment of changes in CAP management. Fourth, 45.7% of CAP patients received antibiotics before hospital admission and before specimen collection, which may reduce the detection of some bacterial infections, such as *Streptococcus pneumoniae*. Urinary antigen testing for *Streptococcus pneumoniae* was performed only in 2.6% of total population. Therefore, the bacterial pathogens identified in this study may not be representative of all

For peer review only - http://bmjopen.bmj.com/site/about/guidelines.xhtml

bacterial causes of CAP in the source patient populations for this study. Finally, while we included adolescents, the majority of patients were adult CAP patients, and our findings do not apply to children hospitalized with CAP.

In conclusion, we characterized adolescents and adults hospitalized for CAP in China and identified several problems suggesting the over-use of healthcare resources in CAP management. This suggests that education and training of clinicians on current CAP guidelines in China are needed to improve clinical management and could also result in substantial cost-saving in healthcare expenditures for CAP patients. The multi-center hospital network can serve as a platform for conducting intervention studies for hospitalized CAP patients in the future, utilizing the baseline data from this observational study.

Acknowledgments

We thank Dr. Jay Purdy (Senior Director, Anti-infectives, Pfizer Inc, 500 Arcola Rd, F3203, Collegeville, PA 19426), Francesco Blasi (Respiratory Unit, IRCCS Fondazione Ca' Granda Ospedale Maggiore Policlinico, Dept of Pathophysiology and Transplantation, University of Milan, Milan, Italy) , and Richard G. Wunderink (Division of Pulmonary and Critical Care Medicine, Department of Medicine, Northwestern University Feinberg School of Medicine, Chicago, IL, USA) for his valuable comments on the manuscript.

Contributors Study design: Liang Chen, Fei zhou, Hui Li, Chen Wang, Bin Cao. Data collection: Liang Chen, Fei Zhou, Hui Li, Xiqian Xing, Xiudi Han,Yimin Wang, Chunxiao Zhang, Lijun Suo, Jinxiang Wang, Guohua Yu, Guangqiang Wang, Xuexin

For peer review only - http://bmjopen.bmj.com/site/about/guidelines.xhtml

Yao, Hongxia Yu, Lei Wang, Meng Liu, Chunxue Xue, Bo Liu, Xiaoli Zhu, Yanli Li, Ying Xiao, Xiaojing Cui, Lijuan Li. Statistical analysis: Liang Chen, Fei Zhou, Hui Li, Xiudi Han. Writing: Liang Chen, Bin Cao, Timothy M. Uyeki. All authors take full responsibility for the study design, data analysis and interpretation, and preparation of the manuscript. All authors approved the final draft manuscript.

Funding This work was funded by National Science Grant for Distinguished Young Scholars (81425001/H0104); National Key Technology Support Program from Ministry of Science and Technology (2015BAI12B11); The Beijing Science and Technology Project (D151100002115004)

Disclaimer The views expressed are those of the authors and do not necessarily reflect the official policy of the Centers for Disease Control and Prevention.

Competing interests None declared.

Data sharing statement No additional data available

References

1. Miniño AM, Heron MP, Smith BL. Deaths: preliminary data for 2004. *Natl Vital Stat Rep* 2006;54:1-49.

2.File TM Jr, Marrie TJ. Burden of community-acquired pneumonia in North American adults. *Postgrad Med* 2010;122:130-41.

3. Almirall J, Bolíbar I, Vidal J, *et al*. Epidemiology of community-acquired pneumonia in adults: a population-based study. *EurRespir J* 2000;15:757-63.

4. Jokinen C, Heiskanen L, Juvonen H, *et al*. Incidence of community-acquired pneumonia in the population of four municipalities in eastern Finland. *Am J*

For peer review only

Epidemiol 1993;137:977-88.

5. Woodhead MA, Macfarlane JT, McCracken JS, *et al*. Prospective study of the aetiology and outcome of pneumonia in the community. *Lancet* 1987;1:671-4.

6. Miniño AM, Heron MP, Smith BL. Deaths: preliminary data for 2004. *Natl Vital Stat Rep* 2006;54:19

7. Health, United States, 2012: with special features on emergency care. Hyattsville, MD: National Center for Health Statistics, 2013:297-299.

8. Uematsu H, Kunisawa S, Sasaki N, *et al*. Development of a risk-adjusted in-hospital mortality prediction model for community-acquired pneumonia: a retrospective analysis using a Japanese administrative database. *BMC Pulm Med* 2014;14:203-210

9.Kim HI, Kim SW, Chang HH, *et al*. Mortality of community-acquired pneumonia in Korea: assessed with the pneumonia severity index and the CURB-65 score. *J Korean Med Sci* 2013;28:1276-82.

10. National Health and Family Planning Commission of the People's Republic of China. China health and family planning statistics yearbook 2013. (Available at: http://www.nhfpc.gov.cn/htmlfiles/zwgkzt/ptjnj/year2013/index2013.html)

11. Jain S, Self WH, Wunderink RG, *et al*. Community-Acquired Pneumonia Requiring Hospitalization among U.S. Adults. *N Engl J Med* 2015;373:415-27.

12. Zhou QT, He B, Zhu H. Potential for cost-savings in the care of hospitalized low-risk community-acquired pneumonia patients in China.*Value Health* 2009;12:40-6.

For peer review only - http://bmjopen.bmj.com/site/about/guidelines.xhtml

13. Ramirez JA, Vargas S, Ritter GW, *et al*. Early switch from intravenous to oral antibiotics and early hospital discharge: a prospective observational study of 200 consecutive patients with community-acquired pneumonia. *Arch Intern Med* 1999;159: 2449–54.

14. Guidelines for the diagnosis and treatment of adult community acquired pneumonia in China (2016 Edition). *Zhong hua Jie He He Hu Xi Za Zhi* 2016;39: 241-2

15. American Thoracic Society. Infectious Diseases Society of America. Guidelines for the management of adults with hospital-acquired, ventilator-associated, and healthcare-associated pneumonia. *Am J Respir Crit Care Med* 2005; 171:388-416

16. Maruyama T, Fujisawa T, Okuno M, *et al*. A new strategy for healthcare-associated pneumonia: a 2-year prospective multicenter cohort study using risk factors for multidrug-resistant pathogens to select initial empiric therapy. *Clin Infect Dis* 2013;57:1373-83.

17. Qi F, Zhang GX, She DY *et al*.Healthcare-associated Pneumonia: Clinical Features and Retrospective Analysis Over 10 Years. *Chin Med J (Engl)* 2015;128:2707-13.

18. Seong GM, Kim M, Lee J, *et al*. Healthcare-Associated Pneumonia among Hospitalized Patients: Is It Different from Community Acquired Pneumonia? *Tuberc Respir Dis (Seoul)* 2014;76:66-74.

19. Lim WS, van der Eerden MM, Laing R, *et al*. Defining community acquired pneumonia severity on presentation to hospital: an international derivation and validation study. *Thorax* 2003;58:377-82.

For peer review only - http://bmjopen.bmj.com/site/about/guidelines.xhtml

20. Fine MJ, Auble TE, Yealy DM, *et al*. A prediction rule to identify low-risk patients with community-acquired pneumonia. *N Engl J Med* 1997;336:243–50.

21. Shorr AF, Zilberberg MD, Reichley R, *et al*. Validation of a clinical score for assessing the risk of resistant pathogens in patients with pneumonia presenting to the emergency department. *Clin Infect Dis* 2012;54:193–8

22. Aliberti S, Di Pasquale M, Zanaboni AM, *et al*. Stratifying risk factors for multidrug-resistant pathogens in hospitalized patients coming from the community with pneumonia. *Clin Infect Dis* 2012;54:470–8

23.American Thoracic Society. Infectious Disease Society / American Thoracic Society consensus guideline on the management of community-acquired pneumonia in adults. *Clin Infect Dis* 2007; 44 Suppl 2:S27-72

24. Broulette J, Yu H, Pyenson B, *et al*. The Incidence Rate and Economic Burden of Community-Acquired Pneumonia in a Working-Age Population. *Am Health Drug Benefits* 2013;6:494-503.

25. Niederman MS, McCombs JS, Unger AN, *et al*. The cost of treating community-acquired pneumonia. *Clin Ther* 1998;20:820-37.

26. Rozenbaum MH, Mangen MJ, Huijts SM, *et al*. Incidence, direct costs and duration of hospitalization of patients hospitalized with community acquired pneumonia: A nationwide retrospective claims database analysis. *Vaccine* 2015;33 :3193-9.

27. Coley CM, Li YH, Medsger AR, *et al*. Preferences for home vs hospital care among low-risk patients with community-acquired pneumonia. *Arch Intern Med*

For peer review only - http://bmjopen.bmj.com/site/about/guidelines.xhtml

1996;156:1565-71

28. Carratalà J, Fernández-Sabé N, Ortega L, *et al*. Outpatient care compared with hospitalization for community-acquired pneumonia: a randomized trial inlow-risk patients. *Ann Intern Med* 2005;142:165-72.

29. Rhew DC, Riedinger MS, Sandhu M, *et al*. A prospective, multicenter study of a pneumonia practice guideline. *Chest* 1998;114:115–9.

30.Halm EA, Switzer GE, Mittman BS, *et al*. What factors influence physicians' decisions to switch from intravenous to oral antibiotics for community-acquired pneumonia? *J Gen Intern Med* 2001;16:599–605.

31. Goss CH, Rubenfeld GD, Park DR, *et al*. Cost and incidence of social comorbidities in low-risk patients with community-acquired pneumonia admitted to a public hospital. *Chest* 2003; 124:2148–55.

32. Aschenbrenner DS. The FDA revises Boxed Warning For Fluoroquinolones-Again. *Am J Nurs* 2016;116:22-3.

33. Tillotson GS. FDA and the safe and appropriate antibiotic use of fluoroquinolones. *Lancet Infect Dis* 2016;16:e11-2.

34. Lu ZK, Yuan J, Li M, *et al*. Cardiac risks associated with antibiotics: azithromycin and levofloxacin. *Expert Opin Drug Saf* 2015;14:295-303.

35.Rush B, Wormsbecker A, Stenstrom R, *et al*. Moxifloxacin Use and Its Association on the Diagnosis of Pulmonary Tuberculosis in An Inner City Emergency Department. *J Emerg Med* 2016;50:371-5.

36. Mirza IA, Khan FA, Khan KA, *et al*. Extensively and pre-extensively drug

For peer review only - http://bmjopen.bmj.com/site/about/guidelines.xhtml

resistant tuberculosis in clinical isolates of multi-drug resistant tuberculosis using classical second line (levofloxacin and amikacin). *J Coll Physicians Surg Pak* 2015;25:337-41.

37.Qu J, Gu L, Wu J, *et al*. Accuracy of IgM antibody testing, FQ-PCR and culture in laboratory diagnosis of acute infection by Mycoplasma pneumoniae in adults and adolescents with community-acquired pneumonia. *BMC Infect Dis* 2013;13:172-7.

38. Angrup A, Chaudhry R, Sharma S, *et al*. Application of real-time quantitative polymerase chain reaction assay to detect Legionella pneumophila in patients of community-acquired pneumonia in a tertiary care hospital. *Indian J Med Microbiol* 2016;34:539-543.

39. Bartlett JG. Diagnostic test for etiologic agents of community-acquired pneumonia. *Infect Dis Clin North Am* 2004;18:809-27.

40. Sayan M, Kilinç O, Yüce A, *et al*. Seropositivity against atypical pneumonia agents demonstrated in patients with community-acquired pneumonia. *Mikrobiyol Bul* 2003;37:247-53.

41. Salo P, Leinonen M. Microbiological investigations. *Semin Respir Infect* 1999;14:128-34.

42. Liu YN, Chen MJ, Zhao TM, *et al*. A multicentre study on the pathogenic agentsin 665 adult patients with community-acquired pneumonia in cities of China. *Zhong hua Jie He He Hu Xi Za Zhi* 2006;29:3-8.

For peer review only - http://bmjopen.bmj.com/site/about/guidelines.xhtml

For peer review only

For peer review only - http://bmjopen.bmj.com/site/about/guidelines.xhtml

Appendix 1: Details of Participating centers

Name of the hospital	Province, city	2nd and 3rd level hospital	Teaching Hospital	Beds	Staff of Clinical Microbioloy Lab
Beijing Chao-Yang Hospital Affiliated to Capital Medical University	Beijing	3rd	Yes	1400	11
Beijing Jishuitan Hospital 4th Medical College of Peking University	Beijing	3rd	Yes	1500	10
Beijing Luhe Hospital Affiliated to Capital Medical University	Beijing	3rd	Yes	1042	5
Qingdao Municipal Hospital	ShanDong, Qingdao	3rd	Yes	1200	4
Qilu Hospital Of Shandong University(Qindao)	ShanDong, Jinan	3rd	Yes	1200	6
Beijing Huimin Hospital	Beijing	2nd	Yes	500	2
Linzi District People's Hospital	ShanDong, Zibo	2nd	Yes	1200	5
The 2nd Hospital of Beijing Corps, Chinese Armed Police Forces	Beijing	3rd	Yes	450	2
China-Japan Friendship Hospital	Beijing	3rd	Yes	1610	9
Yan'an Hospital Affiliated to Kunming Medical University	Kunming, Yan'an	3rd	Yes	1302	4

For peer review only

Yantai Yuhuangding Hospital	Shangdong, Yantai	3rd	Yes	3000	6
Rizhao Chinese Medical Hospital Affiliated to Shandong Chinese Medical University	Shangdong, Rizhao	3rd	Yes	1212	8
Weifang NO.2 People's Hospital	Shangdong, Weifang	3rd	Yes	1006	8

Definition of 2nd and 3rd level hospital in China:

The 2nd level hospital was defined as a hospital providing medical, prevention, health care and rehabilitation services to multiple communities (with a radius of population more than 100,000 peoples); the 3rd level hospital was defined as a hospital providing medical service to the whole country beyond cities and provinces, with comprehensive medical, teaching and research ability.

For peer review only - http://bmjopen.bmj.com/site/about/guidelines.xhtml

Appendix 2: ICD-10

Influenza with pneumonia, other influenza virus identified	J10.0
Influenza with pneumonia, virus not identified	J11.0
Virus pneumonia, not elsewhere classified	J12
Adenoviral pneumonia	J12.0
Respiratory syncytial virus pneumonia	J12.1
Parainfluenza virus pneumonia	J12.2
Other virus pneumonia	J12.8
Viral pneumonia,unspecified	J12.9
Pneumonia due to *Streptococcus pneumoniae*	J13
Pneumonia due to *Haemophilus influenzae*	J14
Bacterial pneumonia, not elsewhere classified	J15
Pneumonia due to *Klebsiella pneumoniae*	J15.0
Pneumonia due to *Pseudomonas spp.*	J15.1
Pneumonia due to *Staphylococcus*	J15.2
Pneumonia due to *Streptococcus spp*., group B	J15.3
Pneumonia due to other *streptococci*	J15.4
Pneumonia due to *Escherichia coli*	J15.5
Pneumonia due to other aerobic Gram-negative bacteria	J15.6
Pneumonia due to Mycoplasma pneumoniae	J15.7
Other bacterial pneumonia	J15.8

For peer review only

Bacterial pneumonia, unspecified	J15.9
Pneumonia due to other infectious organisms, not elsewhere classified	J16
Chlamydia pneumonia	J16.0
Pneumonia due to other specified infectious organisms	J16.8
Pneumonia due to other specified infectious organism	J16.8
Pneumonia in diseases classified elsewhere	J17*
Pneumonia in bacterial diseases classified elsewhere	J17.0*
Pneumonia in viral diseases classified elsewhere	J17.1*
Pneumonia in mycoses	J17.2*
Pneumonia in other diseases classified elsewhere	J17.8*
Pulmonary mycobacterial infection	A31.0
Pulmonary actinomycosis	A42.0
Pulmonary nocardiosis	A43.0
Legionnaires' disease	A48.1
Varicella pneumonia	B01.2+
Measles complicated by pneumonia	B05.1+
Cytomegaloviral pneumonitis	B25.0+
Pulmonary candidiasis	B37.1
Acute pulmonary coccidioidomycosis	B38.0
Acute pulmonary histoplasmosis capsulati	B39.0
Acute pulmonary blastomycosis	B40.0

For peer review only

Pulmonary paracoccidioidomycosis	B41.0
Pulmonary sporotrichosis	B42.0+
Invasive pulmonary aspergillosis	B44.0
Other pulmonary aspergillosis	B44.1
Pulmonary cryptococcosis	B45.0
Pulmonary mucormycosis	B46.0
Pneumonia, organism unspecified	J18
Bronchopneumonia, unspecified organism	J18.0
Lobar pneumonia, unspecified	J18.1
Hypostatic, pneumonia, unspecified	J18.2
Other pneumonia, organism unspecified	J18.8
Pneumonia, unspecified	J18.9

For peer review only

Appendix 3: Case Report Form Of Patients Hospitalized With CAP/HCAP

**Code:** R-			
**Name:**	**Gender:** ○Male ○Female		
**Age:** ______years old	**Nationality:** ○Han ○Others		
**Eight:** ______cm	**Weight:** ___ kg		
**ID Number:**			
**Date Of Admission:** ______Y____M____D			
**Case Number :**	**ID Number:**		
**Admission Form:**	○Outpatience	○Emergency	○Transfers
**Tel:**	**Cell Phone:**		
**Provider Payments:** ○Social Medical Insurance ○New Rural Cooperative Medical System ○ Medical Services At State Expense ○ Commercial Medical Insurance ○Self-paying ○Others			

Study Director: Bin Cao

Team Members: Liang Chen、Hu Li、Meng Liu、Xiudi Han、Xiaoli Zhu 、Bo Liu、Jinxiang Wang、Xuexin Yao、Chunxiao Zhang、Shujing Shi、Fei Zhou、Chunxue Xue、Yanli Li、Donghao Yu （Beijing Chao-Yang Hospital 001；Beijing Jishuitan Hospital 002；Beijing Luhe Hospital 003；Qingdao Municipal Hospital 004;Qilu Hospital Of Shandong University(Qindao) 005； Beijing Huimin Hospital 006； Linzi District People's Hospital 007； The 2nd Hospital of Beijing Corps,Chinese Armed Police Forces 008； China-Japan Friendship Hospital 009;Yan'an Hospital Affliated to Kunming Medical University 010）

For peer review only - http://bmjopen.bmj.com/site/about/guidelines.xhtml

For peer review only

Inclusion Criteria:

1. **Age ≥14 years old**
2. **Onset in community**
3. **Chest X-ray or CT scan showing infiltration or interstitial changes, with or without pleural effusion**
4. **Any one of pneumonia clinical manifestations, including:**

(a) Recent cough, sputum or aggravation of respiratory symptoms, the emergence of purulent sputum, with or without chest pain;

(b) Fever (axillary temperature ≥37.3°C) or hypothermia (axillary temperature <36 °C);

(c) Signs of pulmonary consolidation and (or) moist rales;

(d) WBC>10×10^9/L, or <4×10^9/L, with or without nucleus left.

Meet criteria 1,2 ,3 and anyone of criteria 4

Exclusion Criteria:

1. **Lung infiltrate or interstitial changes which can be interpreted as lung cancer, pulmonary tuberculosis, non-infectious interstitial lung diseases, pulmonary edema, atelectasis, pulmonary embolism, pulmonary eosinophil infiltration, pulmonary vasculitis;**
2. **HIV positive**
3. **Readmission within 72 hours after discharging.**

For peer review only - http://bmjopen.bmj.com/site/about/guidelines.xhtml

Part 1: Baseline Characteristics

Underlying Disease	
COPD ○Y ○N	Asthma ○Y ○N
Bronchiectasis ○Y ○N	Malignancy ○Y ○N
Sleep Apnea Syndrome ○Y ○N	Congestive Heart Failure ○Y ○N
Coronary Heart Disease ○Y ○N	Hypertention ○Y ○N
Peripheral Vascular Diseases ○Y ○ N	Diabetes Mellitus ○Y ○N
Cerebrovascular Disease ○Y ○N	Autoimmune Diseases [a] ○Y ○ N
Chronic Viral Hepatitis ○Y ○N	Cirrhosis ○Y ○N
Hematological Malignancy ○Y ○N	Organ /bone Marrow Transplantation ○Y ○N
Immunosuppressive Therapy [b] ○Y ○N	Chemotherapy/Radiotherapy Within 6 Months ○Y ○N
Chronic Renal Diseases ○Y ○N	Splenectomy ○Y ○N

Note: aSLE, Sjogren's syndrome, rheumatoid arthritis, polymyositis / dermatomyositis, systemic vasculitis, ankylosing spondylitis, inflammatory bowel disease, hyperthyroidism, etc;; b.Anti-rejection drugs

With The Following Situation	
Pregnancy	○Y ○N ○Unknown;<br>If Y, Pregnancy___weeks。
Within 6 months after delivery	○Y ○N ○Unknown;<br>If Y, ___weeks after delivery
Smoking	○Y ○N ○Former Smoker ○Unknown<br>If Y, Smoked For__years, ___cigarettes/day;<br>If Former Smoker, Smoked For___years, ___cigarettes/day ,GivenUp For______years
Alcoholism [a]	○Y ○N ○Unknown
Risk factors for inhalation [b]	○Y ○N ○Unknown
Contact Children In Day-care Center	○Y ○N ○Unknown
Bed Ridden (≥2months)	○Y ○N ○Unknown
Long-term inhaled Corticosteroid used[d]	○Y ○N ○Unknown
Long-term oral Corticosteroid use[c]	○Y ○N ○Unknown;<br>If Y, Name Of Corticosteroid: _______,

For peer review only - http://bmjopen.bmj.com/site/about/guidelines.xhtml

	Dose___mg/day，For______days
**Oral Statin Drugs**	○Y ○N ○Unknown
**History Of CAP Within One Year**	○Y ○N ○Unknown
**Influenza Vaccine Within 1 Year**	○Y ○N ○Unknown
***Streptococcus pneumoniae* Vaccine Within 5 Years**	○Y ○N ○Unknown

Note：**a**：drinking more than 5 bottles of beer (500ml / bottle) or half a catty liquor once in 2 weeks; or drinking more than 2.5 bottles of beer (500ml / bottle) or 2 ounc of white spirit per day for more than five years；**b:** Inhalation risk factors included choking, drowning, nasal feeding, pseudobulbar palsy, dementia, coma, poisoning, Parkinson's disease； **c:** Long-term oral corticosteroids was defined as: oral prednisone ≥10mg / d or equivalent doses of other corticosteroids for more than 3 weeks;**d**：Long-term inhaled corticosteroids was defined as: inhaled corticosteroid for more than 30 days, the daily dose wasn't limited.

**Risk Factors Of Health-Care Acquired Pneumonia**	
**Hospitalization For 2d Or More In The Preceding 90 Days**	○Y ○N ○Unknown
**Home Infusion Therapy (Including Antibiotics) Or Home Wound Care In 30 Days**	○Y ○N ○Unknown
**Chronic dialysis within 30 Days**	○Y ○N ○Unknown
**Residence In A Nursing Home Or Extended Care Facility**	○Y ○N ○Unknown

Part 2：**Data of This Hospitalization**

1.Signs And Symptoms

**History Of Present Illness**	
**Clinical Manifestation**	
**Date Of Illness Onset ： ________Y______M______D**	
**Fever?** （T≥37.3 ℃）	○Y ○N；If Y，Tmax: _____℃
**Hypothermia?** （T＜36℃）	○Y ○N；If Y，Tmin: _____℃
**Cough?**	○Y ○N
**Sputum?**	○Y ○N；<br>If Y，○Yellow Phlegm ○White Phlegm<br>○Bloody Sputum ○Unknown
**Chest Pain?**	○Y ○N
**Shortness Of Breath?**	○Y ○N
**Sore Throat Or Rhinorrhea**	○Y ○N
**Chill/Shiver**	○Y ○N
**Exhaustion/**	○Y ○N

For peer review only - http://bmjopen.bmj.com/site/about/guidelines.xhtml

**Muscle And Joint Aches//Headache**	
**Darrhea?**	○Y ○N
**Familial Aggregation （2 Epidemiological Related People Suffered From Pneumonia In Two Weeks）?**	○Y ○N
**Physical Examination<br>(The Worst Value Of The Day On Admission)**	
**Tmax，℃**	
**Tmin，℃**	
**HR, beats/min**	
**RR, breaths/min**	
**BP(Systolic Pressure / Diastolic Pressure), mmHg**	
**Disorder Of Consciousness?**	○Y ○N
**Cyanosis?**	○Y ○N
**Physical Signs Of Lung:**	**Moist rales ○Y ○N<br>Dry rales ○Y ○N**
**Edema Of Legs?**	○Y ○N;<br>**If Y，Asymmetric Edema Of Legs?** ○Y ○N

3.Pre-hospital Medical Data ○Y ○N

**Radiology**		
**Chest X-ray**<br>○Y<br>○N<br>○Unknown<br>**Date of Examination:**<br>____Y___M__D	**Site Of Pneumonia**	○**Bilateral Lung** ○**Unilateral Lung**
	**Site Of Pneumonia**	○**Superior Lobe Of Right Lung**<br>○**Middle Lobe Of Right Lung**<br>○**Inferior Lobe Of Right Lung**<br>○**Superior Lobe Of Left Lung**<br>○**Inferior Lobe Of Left Lung**<br>○**Unknown**
	**Plural effusion**	○N ○Left ○Right ○Bilateral
	**Cavity**	○Y ○N
	**consolidation**	○Y ○N
	**Interstitial Change**	○Y ○N
	**Infiltration**	○Y ○N
**Lung CT**<br>○Y<br>○N<br>○Unknown<br>**Date of Examination:**<br>____Y___M__D	**Alveolar Infiltration**	○**Superior Lobe Of Right Lung**<br>○**Middle Lobe Of Right Lung**<br>○**Inferior Lobe Of Right Lung**<br>○**Superior Lobe Of Left Lung**<br>○**Inferior Lobe Of Left Lung**<br>○**Bilateral Diffuse Infiltration**<br>○**Unilateral Diffuse iInfiltration**

For peer review only - http://bmjopen.bmj.com/site/about/guidelines.xhtml

	Plural effusion	○N ○Left ○Right ○Bilateral		
	Cavity	○Y ○N		
	consolidation	○Y ○N		
	Abscesses	○Y ○N		
	Patchy Shadow	○Y ○N		
	Interstitial change	○Y ○N		
**Microbiological Examination**				
**Microbiological Examination Before Admission** ○Y ○N ○Unknown				
**If Y：**<br>**Date Of Specimen Collection：______Y_____M_____D**<br>**Specimen Type: ○Sputum ○Blood ○BALF ○Asopharyngeal Swab**<br>**○Endotracheal Aspirate ○Plural Effusion ○Urine**<br>**Microbiological Examination Results:________________________________**				
**Treatment Before Admission**				
**Antimicrobials Before Admission** ○Y ○N ○Unknown				
**Drug name（Generic Name And Trade Name）**	**Route Of Administration**	**Drug Regimen**	**Start Time**	**TerminalTime**
eg：Ceftriaxone（罗氏芬）	● Intravenous ○Oral	2.0g ，Qd	2014．9．1	2014．9．8
	○ Intravenous ○Oral			
	○ Intravenous ○Oral			
	○ Intravenous ○Oral			
	○ Intravenous ○Oral			
**Antiviral Drug Use Before Admission** ○Y ○N ○Unknown				
	○Intravenous ○Oral<br>○ Inhalation			
	○Intravenous ○Oral<br>○ inhalation			
**Corticosteroid Use Before Admission** ○Y ○N ○Unknown				
	○Intravenous ○Oral<br>○ inhalation			
	○Intravenous ○Oral<br>○ inhalation			
**Vasopressor Use Before Admission** ○Y ○N ○Unknown				
**If Y，Start Time：______________**		**Terminal Time：______________**		
**Invasive Ventilation Before Admission** ○Y ○N ○Unknown				
**If Y，Start Time：______________**		**Terminal Time：______________**		

For peer review only

For peer review only - http://bmjopen.bmj.com/site/about/guidelines.xhtml

Note:b.Vasopressors：Norepinephrine、Dopamine、Dobutamine、Metaraminol、adrenaline

4.Laboratory Examination In 24hr On Admission

Category	Item	Value	Unit
Blood Routine	WBC		*10^9/L
	Neu		*10^9/L
	Lym		*10^9/L
	HGB		g/L
	HCT		%
	PLT		*10^9/L
Biochemistry	ALB		g/L
	LDH		U/L
	AST		U/L
	ALT		U/L
	ALP		U/L
	TBIL		umol/L
	DBIL		umol/L
	CK		U/L
	BUN		mmol/L
	Cr		mmol/L
	Glu		mmol/L
	K		mmol/L
	Na		mmol/L
Serum Detection	ESR		mm/h
	CRP		mg/dL
	PCT		ng/ml
	D-dimer		ng/ml
	PT		s
	APTT		s
	INR		
	BNP		pg/ml
	Ferritin		ug/l

5.Blood Gas Analysis、Radiology and Ultrasonography After Admission

Category	Item	Value

For peer review only - http://bmjopen.bmj.com/site/about/guidelines.xhtml

Blood gas analysis (The Worst Value In 24hr On Admission)	Oxygen Therapy※<br>○Y ○N		○Oxygen Inhalation Through Nasal Tube_____L/min<br>○Oxygen Inhalation Through Venturi Mask______%<br>○Oxygen Inhalation Through Oxygen Masks__L/min<br>○Non-invasive Ventilation<br>○Invasive Ventilation<br>○Unknowen
	FiO_2		
	pH		
	PO_2(mmHg)		
	PCO_2(mmHg)		
	SaO_2		
	Actual Bicarbonate (mmol/l)		
	Lac (mmol/l)		
Radiology (In 24hr On Admission)	Chest X-ray<br>○Y ○N	Alveolar Infiltration	○Superior Lobe Of Right Lung<br>○Middle Lobe Of Right Lung<br>○Inferior Lobe Of Right Lung<br>○Superior Lobe Of Left Lung<br>○Inferior Lobe Of Left Lung<br>○Bilateral Diffuse Infiltration<br>○Unilateral Diffuse Infiltration
		Plural effusion	○N ○Left ○Right ○Bilateral
		Cavity	○Y ○N
		consolidation	○Y ○N
		Patchy Shadow	○Y ○N
		Interstitial Change	○Y ○N
	Lung CT<br>○Y ○N	Alveolar Infiltration	○Superior Lobe Of Right Lung<br>○Middle Lobe Of Right Lung<br>○Inferior Lobe Of Right Lung<br>○Superior Lobe Of Left Lung<br>○Inferior Lobe Of Left Lung<br>○Bilateral Diffuse Infiltration<br>○Unilateral Diffuse Infiltration
		Plural effusion	○N ○Left ○Right ○Bilateral
		Cavity	○Y ○N
		consolidation	○Y ○N
		Patchy Shadow	○Y ○N

For peer review only - http://bmjopen.bmj.com/site/about/guidelines.xhtml

		**Interstitial Change**	**○Y ○N**
		**Alveolar Infiltration**	**○Superior Lobe Of Right Lung<br>○Middle Lobe Of Right Lung<br>○Inferior Lobe Of Right Lung<br>○Superior Lobe Of Left Lung<br>○Inferior Lobe Of Left Lung<br>○Bilateral Diffuse Infiltration<br>○Unilateral Diffuse Infiltration**
**Ultrasonography**	**Lower Limb Vascular Ultrasound Exam**	**Venous Thrombosis**	**○N ○Left ○Right<br>○Bilateral ○ Unexamined**

Note※The Worst Value Of Blood Gas Analysis And FiO2 At That Time.

For peer review only

6.Keep Detailed Records Of The Following Time Points,And Write down The Code In The First Row Of The Table：

①The 4th day（The Day On Admission Is The 1st Day）；②The day of changing Antibiotics in 14 days After Admission；③The 14th day after Admission ；④The Day Of Discharging

For peer review only

For peer review only - http://bmjopen.bmj.com/site/about/guidelines.xhtml

Category	Item(Unit)	The Reason and The Date						
Vital Signs	Disorder Of Consciousness							
	Tmax（℃）							
	Tmin(℃)							
	HR（beats/min）							
	RR（breaths/min）							
	BP(/ mmHg）							
Symptoms<br>1. Exacerbation<br>2. Alleviation<br>3. No-change	Cough							
	Sputum							
	Chest Pain							
	Shortness Of Breath							
	Moist Rales							
	Dry Rales							
Blood Routine	WBC（*10^9/L）							
	Neu（*10^9/L）							
	Lym（*10^9/L）							
	HGB（g/L）							
	HCT（%）							
	PLT（*10^9/L）							
Biochemistry	ALB（g/L）							
	LDH（U/L）							
	AST（U/L）							
	ALT（U/L）							
	ALP（U/L）							
	TBIL（umol/L）							
	DBIL（umol/L）							
	CK（U/L）							
	CTNI（ng/ml）							
	BUN（mmol/L）							
	Cr（mmol/L）							
	[illegible]							

For peer review only

Note：a. Direct Microscopy of sputum is not included

For peer review only

For peer review only - http://bmjopen.bmj.com/site/about/guidelines.xhtml

7.Treatment During Hospitalization

Antibiotics Use ○Y ○N				
Drug Name （Generic Name And Trade Name）	Route Of Administration	Drug Regimen	Start Time	Terminal Ttime
eg: Ceftriaxone （罗氏芬）	● Intravenous ○Oral	2.0g，Qd	2014-3-2	2014-4-5
	○ Intravenous ○Oral			
	○ Intravenous ○Oral			
	○ Intravenous ○Oral			
	○ Intravenous ○Oral			
	○ Intravenous ○Oral			
	○ Intravenous ○Oral			
	○ Intravenous ○Oral			
**Antiviral Drugs Use ○Y ○N**				
Drug name （Generic Name And Trade Name）	Route Of Administration	Drug Regimen	Start Time	Terminal Time
	○ Intravenous ○Oral ○ Inhalation			
	○ Intravenous ○Oral ○ Inhalation			
	○ Intravenous ○Oral ○ Inhalation			
**Glucocorticoids Use ○Y ○N**				
Drug name （Generic Name And Trade Name）	Route Of Administration	Drug Regimen	Start Time	Terminal Time
	○ Intravenous ○Oral ○Inhalation			
	○ Intravenous ○Oral ○ Inhalation			
**Vasopressors Use ○Y ○N**				
Drug Name	Route Of Administration	Drug Regimen	Start Time	Terminal Ttime
**Immunoregulation Drugs（Including Intravenous Immunoglobulin 、Thymosins）○Y ○N**				
Drug Name	Route of administration	Drug Regimen	Start time	Terminal time

For peer review only

For peer review only - http://bmjopen.bmj.com/site/about/guidelines.xhtml

Alternative/ Supportive Treatment			
Item	Use	Start Time	Terminal Time
Continuous Venous-venous Hemofiltration	○Y ○N		
Extracorporeal Membrane Oxygenation （ECMO）	○Y ○N		
Non-invasive Ventilation	○Y ○N		
Invasive Ventilation	○Y ○N		

8、Measurement Of T Lymphocyte Subsets

Date of specimen collection: ___Y ____M ____D

T lymphocyte subsets	CD4		/ml
	CD8		/ml
	CD4%		
	CD8%		
	NK		/ml
	NKT		/ml
	CD4/CD8		

Note：Without Time Limitation

9.Microbiological Examination

(1).Microbiological Examination In 48hrs After Admission ○Y ○N

Microbiological Examination For Sputum Or eEndotracheal aAspiration<br>Date Of Specimen Collection：__Y___M___D	
Item	Results
Direct Microscopy	○Good Quality Sputum （ > 25 leukocytes and < 10 epithelial cells per × 100 magnification field）<br>○Not Good Quality Sputum<br>○Unknown
	○G+ Cocci ○G+ Bacillus ○G- Cocci<br>○G- Bacillus ○Positive Acid-fast Stain ○None

For peer review only - http://bmjopen.bmj.com/site/about/guidelines.xhtml

Bacteria Culture	○Streptococcus pneumoniae<br>○Haemophilus influenzae<br>○Pseudomonas aeruginosa<br>○Enterobacter cloacae<br>○Acinetobacter spp<br>○Stenotrophomonas maltophilia<br>○Escherichia coli<br>○Enterococcus faecium<br>○None Or Normal oropharyngeal flora	○Moraxella catarrhalis<br>○staphylococcus aureus<br>○Klebsiella pneumoniae<br>○Proteus spp<br>○Serratia marcescens<br>○Enterobacter aerogenes<br>○Enterococcus faecalis<br>○Others: _____
	**Drug Resistant Bacteria**	
	○Methicillin Resistance Staphylococcus aureus（MRSA)<br>○Vancomycin-resistant Enterococcus<br>Bacteria producing ESBLs:<br>○Escherichia coli<br>○Enterobacter cloacae<br>non - fermentative bacteria.:<br>○Acinetobacter baumannii<br>○Others: ____	 <br><br>○Klebsiella pneumoniae<br>○Serratia marcescens<br><br>○Pseudomonas aeruginosa
	If Streptococcus pneumoniae ， MIC for penicillin____ug/ml;<br>○Not detected	
	If MRSA， MIC for Vancomycin_____ug/ml;<br>○Not detected	
Direct Microscopy	○Fungal Spore<br>○Cryptococcus neoformans	○Fungal Hyphae<br>○None
Fungi Culture	○Spergillus Fumigatus<br>○Aspergillus terreus<br>○Candida Spp<br>○Undetected	○Aspergilusflavus<br>○Mucor Mucedo<br>○Cryptococcus Neoformans<br>○Others: ____
Nucleic Acid Test For Respiratory Virus	○Influenza A H1N1<br>○Influenza A H2N3<br>○Nontypeable Influenza A<br>○Adenovirus<br>○Parainfluenza virus2<br>○Parainfluenza virus 4<br>○Rhinovirus<br>○Coronavirus OC43HKU1<br>○Coronavirus 229ENL63<br>○Bocavirus<br>○EB virus	○Avian influenza H7N9<br>○Influenza A H5N1<br>○Influenza B<br>○Parainfluenza virus 1<br>○Parainfluenza virus 3<br>○Respiratory syncytial virus A<br>○Respiratory syncytial virus B<br>○Enterovirus<br>○Herpes simplex virus<br>○Cytomegalovirus<br>○MERS-CoV

For peer review only - http://bmjopen.bmj.com/site/about/guidelines.xhtml

Nucleic Acid Test For Atypical Etiology	○Mycoplasma pneumoniae ○Chlamydia pneumoniae<br>○Legionella spp

(2).Microbiological Examination For BALF? ○Y ○N

Microbiological examination for BALF（Within One Week After Admission）<br>Date Of Specimen Collection： __Y___M___D	
Item	Results
Direct Microscopy	○G+ Cocci ○G+ Bacillus ○G- Cocci<br>○G- Bacillus ○Positive Acid-fast Stain ○None
Bacteria Culture	○Streptococcus pneumoniae ○Moraxella catarrhalis<br>○Haemophilus influenzae ○staphylococcus aureus<br>○Pseudomonas aeruginosa ○Klebsiella pneumoniae<br>○Enterobacter cloacae ○Proteus spp<br>○Acinetobacter spp ○Serratia marcescens<br>○Stenotrophomonas maltophilia ○Enterobacter aerogenes<br>○Escherichia coli ○Enterococcus faecalis<br>○Enterococcus faecium ○Others： _____<br>○None Or Normal oropharyngeal flora
	Drug Resistant Bacteria
	○Methicillin Resistance Staphylococcus aureus（MRSA)<br>○Vancomycin-resistant Enterococcus<br>Bacteria producing ESBLs：<br>○Escherichia coli ○Klebsiella pneumoniae<br>○Enterobacter cloacae ○Serratia marcescens<br>non - fermentative bacteria.：<br>○Acinetobacter baumannii ○Pseudomonas aeruginosa<br>○Others： ____
	If Streptococcus pneumoniae ， MIC for penicillin____ug/ml；<br>○Not Detected
	If MRSA，MIC for Vancomycin_____ug/ml；<br>○Not Detected
Direct Microscopy	○Fungal Spore ○Fungal Hyphae<br>○Cryptococcus neoformans ○None

For peer review only - http://bmjopen.bmj.com/site/about/guidelines.xhtml

**Fungi Culture**	○Spergillus Fumigatus<br>○Aspergillus terreus<br>○Candida Spp<br>○Undetected	○Aspergilusflavus<br>○Mucor Mucedo<br>○Cryptococcus Neoformans<br>○Others: ____
**Nucleic Acid For Respiratory Virus**	○Influenza A H1N1<br>○Influenza A H2N3<br>○Nontypeable Influenza A<br>○Adenovirus<br>○Parainfluenza virus2<br>○Parainfluenza virus 4<br>○Rhinovirus<br>○Coronavirus OC43HKU1<br>○Coronavirus 229ENL63<br>○Bocavirus<br>○EB virus	○Avian influenza H7N9<br>○Influenza A H5N1<br>○Influenza B<br>○Parainfluenza virus 1<br>○Parainfluenza virus 3<br>○Respiratory syncytial virus A<br>○Respiratory syncytial virus B<br>○Enterovirus<br>○Herpes simplex virus<br>○Cytomegalovirus<br>○MERS-CoV
**Nucleic Acid Test For Atypical Etiology**	○Mycoplasma pneumoniae<br>○Legionella spp	○Chlamydia pneumoniae

(3).Blood Culture In One Week After Admission? ○Y ○N

**Blood Culture (In One Week After Admission)**<br>**Date Of Specimen Collection: __Y___M___D**		
**Item**	**Results**	
**Bacteria Culture**	○Staphylococcus aureus<br>○Haemophilus influenzae<br>○Klebsiella pneumoniae<br>○Proteus spp<br>○Serratia marcescens<br>○Enterobacter aerogenes<br>○Enterococcus faecalis<br>○Others: ___________	○Moraxella catarrhalis<br>○Pseudomonas aeruginosa<br>○Enterobacter cloacae<br>○Acinetobacter spp<br>○Stenotrophomonas maltophilia<br>○Escherichia coli<br>○Enterococcus faecium<br>○None or normal oropharyngeal flora
	**Drug Resistant Bacteria**	
	○Methicillin resistance staphylococcus aureus（MRSA)<br>○Vancomycin-resistant Enterococcus<br>**Bacteria producing ESBLs:**<br>○Escherichia coli<br>○Enterobacter cloacae<br>**non - fermentative bacteria.:**<br>○Acinetobacter baumannii<br>○Others: ____	 <br><br>○Klebsiella pneumoniae<br>○Serratia marcescens<br><br>○Pseudomonas aeruginosa

For peer review only - http://bmjopen.bmj.com/site/about/guidelines.xhtml

	If Streptococcus pneumoniae , MIC for penicillin____ug/ml;<br>○Not Detected
	If MRSA, MIC for Vancomycin_____ug/ml;<br>○Not Detected
Fungi Culture	○Candidiasis albicans ○Candida krusei<br>○Candida tropicalis ○Candida glabrata<br>○Candida parapsilosis ○Cryptococcus neoformans<br>○Aspergillus fumigatus ○Aspergilus flavus<br>○Aspergillus terreus ○Mucor Mucedo<br>○Undetected ○Others: ____

(4)、Pleural Effusion? ○Y ○N

Pleural Effusion Test? ○Y ○N

**Microbiological Examination For Pleural Effusion(Without Time Limitation)**<br>**Date of Specimen collection: __Y___M___D**	
**Pleural Effusion Routine**	
Total Cell Count: ___×10^6/L; Multinuclear Cell: ___×10^6/L;<br>Mononuclear Cells: ___×10^6/L	
**Pleural Effusion Biochemistry**	
LDH: ___U/L; ADA: ___U/L; Pr: ___g/L<br>Glu:___mmol/L Cl: ___mmol/L	
Item	Results
Bacteria Culture	○Staphylococcus aureus ○Moraxella catarrhalis<br>○Haemophilus influenzae ○Pseudomonas aeruginosa<br>○Klebsiella pneumoniae ○Enterobacter cloacae<br>○Proteus spp ○Acinetobacter spp<br>○Serratia marcescens ○Stenotrophomonas maltophilia<br>○Enterobacter aerogenes ○Escherichia coli<br>○Enterococcus faecalis ○Enterococcus faecium<br>○Others: ___________ ○None or Normal Oropharyngeal Flora
	Drug Resistant Bacteria
	○Methicillin resistance staphylococcus aureus(MRSA)<br>○Vancomycin-resistant Enterococcus<br>Bacteria producing ESBLs:<br>○Escherichia coli ○Klebsiella pneumoniae<br>○Enterobacter cloacae ○Serratia marcescens<br>non - fermentative bacteria.:<br>○Acinetobacter baumannii ○Pseudomonas aeruginosa<br>○Others: ____
Fungi Culture	○Candidiasis albicans ○Candida krusei<br>○Candida tropicalis ○Candida glabrata

For peer review only - http://bmjopen.bmj.com/site/about/guidelines.xhtml

	○Candida parapsilosis	○Cryptococcus neoformans
	○Aspergillus fumigatus	○Aspergilus flavus
	○Aspergillus terreus	○Mucor Mucedo
	○Undetected	○Others: _

（5）、Antigen Test In 48hr After Admission？ ○Y ○N

Urinary antigen（in 48hr after admission）<br>Date of specimen collection: __Y___M___D	
Urinary Antigen For *Legionella spp*	○Positive ○Negative ○Undetected
Urinary Antigen For *Streptococcus pneumoniae*	○Positive ○Negative ○Undetected
Throat Swab Aantigen Test（In 48hr After Admission）<br>Date Of Specimen Collection: __Y___M___D	
Respiratory Syncytial Virus Antigen Test	○Positive ○Negative ○Undetected
Influenza A Antigen Test	○Positive ○Negative ○Undetected
Influenza B Antigen Test	○Positive ○Negative ○Undetected

（6）、Antibody Test？ a）○Y ○N

b）If Y , Titer Of Antibody In Paired Serum? ○Y ,Interval___days

○N

Antibody Test（Without Time Limitation）<br>Date Of Specimen Collection: __Y___M___D	
○IgM for *Mycoplasmal pneumonia*	○IgM for Influenza A
○IgG for *Mycoplasmal pneumonia*	○IgM for Parainfluenza
○IgM for *Chlamydia* spp	○IgM for Q fever
○IgG for *Chlamydia* spp.	○IgM for Adenovirus
○IgM for *Legionella spp*	○IgM for Respiratory syncytial virus
○IgG for *Legionella spp*	○IgM for Parainfluenza 1,2,3

For peer review only - http://bmjopen.bmj.com/site/about/guidelines.xhtml

10、Outcomes

(1).Treatment Failure Within 14 Days

Treatment Failure Within 14 Days（Multiple choices）（The Value Of The 1st Day On Admission As The Baseline Data）	
**1.Needs Invasive Ventilation**	○Y ○N
**2.Needs Non-invasive Ventilation**	○Y ○N
**3.Needs Vasopressors**	○Y ○N
**4.Death**	○Y ○N

The Reasons For Treatment Failure		
**1.CAP Progression**	**Pneumonia Progression**	○Y ○N
**2.CAP Complications**	**Pyothorax**	○Y ○N
	**Endocarditis**	○Y ○N
	**Meningitis**	○Y ○N
	**Others:** ____________	
**3.Severe Sepsis Due To CAP**	**ARDS**	○Y ○N
	**Sepsis**	○Y ○N
	**Hepatic Failure**	○Y ○N
	**Renal Ffailure**	○Y ○N
	**Clotting Disorders,**	○Y ○N
	**Encephalopathy**	○Y ○N
	**Others:** __________	
**4.Complications Or Underlying Disease Deterioration**	**Pulmonary Embolism**	○Y ○N
	**Myocardial Infarction**	○Y ○N
	**Arrhythmia**	○Y ○N
	**Gastrointestinal Bleeding**	○Y ○N
	**Congestive Heart Failure**	○Y ○N
	**COPD**	○Y ○N
	**Diabetes Mellitus**	○Y ○N
	**Nephropathy**	○Y ○N
	**Others:** __________	
**5.Complications Due To Treatment**	**Hemopneumothorax**	○Y ○N
	**Allergic To Antibiotics**	○Y ○N
	**HAP/VAP**	○Y ○N
	**Vascular Catheter Infection**	○Y ○N
	**C. Difficile Infection**	○Y ○N
	**Iatrogenic Urinary Tract Infection**	○Y ○N
	**Others:** __________	
**6.Unknown**	○Y ○N	

For peer review only

(2). Complications During Hospitalization

Complications During Hospitalization	
Complications（Multiple Choices） ○Y ○N	
Respiratory Failure ○Y ○N	ARDS ○Y ○N
Heart Failure ○Y ○N	Acute Myocardial Infarction ○Y ○N
Acute Liver Failure ○Y ○N	AcuteRenal Failure ○Y ○N
Septic Shock ○Y ○N	Stroke ○Y ○N
DIC ○Y ○N	Antibiotic Associated Diarrhea ○Y ○N
Arrhythmia ○Y ○N	MODS ○Y ○N
Pulmonary Embolism ○Y ○N	Deep Venous Thrombosis ○Y ○N
Ventilator Associated Pneumonia ○Y ○N	Gastrointestinal Bleeding ○Y ○N
Invasive Aspergillosis ○Y ○N	Mediastinal Emphysema ○Y ○N
Pneumothorax ○Y ○N	Nosocomial Bloodstream Infection ○Y ○N
Others ○Y ○N	If Y: ________

（3）. Outcomes

Clinical Stability Before Discharge	○Y ○N<br>If Y, the date of clinical stability ______ Y ______M ______D.<br>Meet the following seven criteria: Temperature<37.8℃ for more than 24hr; Heart rate ≤100 beats/min ; Respiratory rate ≤24 breaths/min ;Systolic blood pressure ≥90 mm Hg ; Arterial oxygen saturation ≥90% or pO2 ≥ 60 mm Hg on room air ; Ability to maintain oral intake; Normal mental status.
Admitted to RICU/ICU?	○Y ○N<br>If Y, The Date Of Admitted To RICU/ICU: ____Y____M____D<br>The Date Of Transfer From RICU/ICU: ____Y____M____D
Discharging	The Date Of Discharging____Y____M____D<br>Outcome ○Improvement ○Against-advice discharge ○Death<br>If death, The Death Date_____Y____M____D
Direct Cause Of Death (only one choice)	○Severe Pneumonia ○Respiratory Failure<br>○Shock○Heart Failure ○Acute Myocardial Infarction

For peer review only - http://bmjopen.bmj.com/site/about/guidelines.xhtml

	○Acute renal Failure ○Hepatic failure<br>○DIC ○Stroke<br>○Gastrointestinal Bleeding ○Others: ________

10. **Cost And Economy Data**

Total Expenses _______Yuan:

Drugs Cost: __________Yuan , Antimicrobials Cost _________Yuan

Laboratory Testing Expenses: ________Yuan

Bed Charge: __________Yuan

Health Care Worker Labor Cost: _________Yuan

For peer review only

For peer review only - http://bmjopen.bmj.com/site/about/guidelines.xhtml

Appendix 4: Definition of underlying diseases

1) Long-term smoking was defined as: cigarette smokers of 10 cigarettes/d during at least the previous year；

2) Alcoholism was defined as: drinking more than 5 bottles of beer (500ml / bottle) or half a catty liquor once in 2 weeks; or drinking more than 2.5 bottles of beer (500ml / bottle) or 2 ounc of white spirit per day for more than five years；

3) Long-term oral corticosteroids was defined as: oral prednisone ≥10mg / d or equivalent doses of other corticosteroids for more than 3 weeks.[1]

4) Long-term inhaled corticosteroids was defined as: inhaled corticosteroid for more than 30 days, the daily dose wasn't limited;

5) COPD was defined as: persistent airflow limitation, FEV1 / FVC < 70% post bronchodilator;

6) Asthma was defined by the history of respiratory symptoms such as wheeze, cough that varied over time and intensity, together with variable respiratory airway limitation;

7) Hypertension was defined as systolic blood pressure≥140mmHg and /or diastolic blood pressure≥90mmHg in resting status;

8) Coronary heart disease included angina pectoris, myocardial infarction, ischemic cardiomyopathy;

9) Chronic congestive heart failure was defined as cardiomegaly and ejection fraction ≤40%;

For peer review only - http://bmjopen.bmj.com/site/about/guidelines.xhtml

10) Cerebrovascular diseases included transient ischemic attack, cerebral hemorrhage, subarachnoid hemorrhage, cerebral infarction, etc;

11) Diebetes mellitus: included diabetes mellitus type 1 and diabetes mellitus type 2, not included impaired glucose tolerance and impaired fasting glycaemia;

12) Chronic liver disease included chronic viral hepatitis, chronic alcoholic liver disease, chronic fatty liver disease, etc;

13) Chronic kidney disease included diabetic nephropathy, hypertensive renal damage, chronic glomerulonephritis, chronic pyelonephritis, lupus nephritis, IgA nephropathy, nephrotic syndrome, hereditary kidney disease, etc;

14) Connective Tissue Diseases include SLE, Sjogren's syndrome, rheumatoid arthritis, polymyositis / dermatomyositis, systemic vasculitis, ankylosing spondylitis, inflammatory bowel disease, hyperthyroidism, etc;

15) Organ transplantation or bone marrow transplantation included solid organ transplantating, such as liver transplantation, kidney transplantation, lung transplantation or pancreas transplantation, etc and bone marrow transplantation;

16) Aspiration risk factors included choking, drowning, nasal, pseudobulbar palsy, dementia, coma, poisoning, Parkinson's disease.

17) Immunosuppressive therapy: was defined as systmatic glucocorticosteroid (such as prednisone ≥10mg/d for more than 3 weeks in the last month); cyclosporine or azathioprine use within 3 months, and methotrexate use ≥12.5mg/week within 3 months; biological modifiers such as etanercept and infiximab within 3 weeks.

For peer review only

18) Immunocompromised status included HIV(+), chemotherapy/radiotherapy within 6 months, immunosuppressive therapy, organ/bone marrow transplantation, splenectomy, hematological neoplasms.[2]

19) Risk factors for pseudomonal infection was defined as chronic airway disease (bronchiectasis or COPD) and HCAP risk factors according to IDSA/ATS criteria.[3-7]

20) Overtreatment was defined as: i) use of antipseudomonal β-lactams or β-lactams+ fluoquinolones in hospitalized (not in ICU) patients without risk factors for pseudomonal infection; i i) use of β-lactams(antipseudomonal or not)+ fluoquinolones in ICU patients aged< 65yr without risk factors for pseudomonas infection; iii) use of anti-MRSA drugs in hospitalized (not in ICU) patients.[3]

References:

1. Jouneau S, Poineuf JS, Minjolle S, *et al*. Which patients should be tested for viruses on bronchoalveolar lavage fluid? *Eur J Clin Microbiol Infect Dis* 2013 ;32:671-7.

2. Sousa D, Justo I, Domínguez A,*et al*. Community-acquired pneumonia in immunocompromised older patients: incidence, causative organisms and outcome. *Clin Microbiol Infect* 2013 ;19:187-92

3. Guidelines for the diagnosis and treatment of adult community-acquired pneumonia in China(2016 Edition). *Zhonghua Jie He He Hu Xi Za Zhi* 2016; 39: 241-2

4. American Thoracic Society.Infectious Diseases Society of America.Guidelines for the management of adults with hospital-acquired, ventilator-associated, and healthcare-associated pneumonia.*Am J Respir Crit Care Med* 2005; 171:388-416

For peer review only - http://bmjopen.bmj.com/site/about/guidelines.xhtml

5. Maruyama T1, Fujisawa T, Okuno M, *et al*. A new strategy for healthcare-associated pneumonia: a 2-year prospective multicenter cohort study using risk factors for multidrug-resistant pathogens to select initial empiric therapy. *Clin Infect Dis* 2013;57:1373-83.

6. Qi F, Zhang GX, She DY *et al*. Healthcare-associated Pneumonia: Clinical Features and Retrospective Analysis Over 10 Years. *Chin Med J (Engl)* 2015;128:2707-13.

7. Seong GM, Kim M, Lee J, *et al*. Healthcare-Associated Pneumonia among Hospitalized Patients: Is It Different from Community Acquired Pneumonia? *Tuberc Respir Dis (Seoul)* 2014;76:66-74.

For peer review only

For peer review only - http://bmjopen.bmj.com/site/about/guidelines.xhtml

Appendix 5 Empirical antimicrobial regimens according to Chinese CAP guideline

Populations	Common pathogens	Anti-infective agents for initial empirical therapy	Comment
Outpatient treatment (Oral administration is recommended)			
Young adults without underlying disease(s)	*S. pneumoniae*, *M. pneumoniae*, *H. influenzae*, *C. pneumoniae*, influenza virus, adenovirus, *M. catarrhalis*	(1) Aminopenicillins, penicillins-β-lactamase -inhibitor combinations; (2) I or II generation cephalosporins; (3) doxycycline or minocycline; (4) respiratory quinolones; (5) macrolides	(1) Differentiate among bacterial pneumonia, *Mycoplasma*, *Chlamydia* and viral pneumonia based on clinical characteristics; (2) Mild pneumonia caused by *Mycoplasma*, *Chlamydia*, and virus is usually self-limited
Patients with underlying disease(s) or elderly patients (age ≥ 65 years)	*S. pneumoniae*, *H. influenzae*, *Enterobacteriaceae* such as *K. pneumoniae*, *C. pneumoniae*, influenza virus, RSV, *M. catarrhalis*	(1) Penicillins-β-lactamase-inhibitor combinations; (2) II or III generation cephalosporins (oral); (3) respiratory quinolones; (4) penicillins-lactamase -inhibitor combinations, II generation cephalosporins, III generation cephalosporins combined with doxycycline or minocycline or macrolides	Monotherapy with doxycycline or minocycline or macrolides is not recommended in patients with risk factors of resistant *S. pneumoniae* (1), such as age > 65 years, underlying diseases (chronic cardiac, pulmonary, or renal diseases, diabetes mellitus, and immunosuppression), alcoholism, and β-lactams treatment within 3 months.
Inpatient treatment, non-ICU (Intravenous or oral administration)			

For peer review only - http://bmjopen.bmj.com/site/about/guidelines.xhtml

Young adults without underlying disease(s)	*S. pneumoniae*, *H. influenzae*, *M. catarrhalis*, *S. aureus*, *M. pneumoniae*, *C. pneumoniae*, influenza virus, adenovirus, other respiratory tract viruses	(1) Penicillin G, aminopenicillins, penicillins-β-lactamase-inhibitor combinations; (2) II or III generation cephalosporins, cephamycins, oxacephems; (3) the above drugs combined with doxycycline, minocycline or macrolides; (4) respiratory quinolones; (5) macrolides	(1) Only 1.9% the *S. pneumoniae* isolates from adult CAP are resistant to intravenous penicillins in China. The percentage of intermediate strains is only about 9%. Intravenous penicillins are still effective in hospitalized patients infected with penicillin-intermediate *S. pneumoniae* when increasing the dosage (23, 161); (2) When atypical pathogens are suspected, doxycycline or minocycline or respiratory quinolones are preferred. Macrolides can be used in regions with lower resistance rate to mycoplasma
Patients with underlying disease(s) or elderly patients (age ≥ 65 years)	*S. pneumoniae*, *H. influenzae*, *Enterobacteriaceae* such as *K. pneumoniae*, influenza virus, RSV, *M. catarrhalis*, anaerobic bacteria, *Legionella*	(1) Penicillins-β-lactamase-inhibitor combinations; (2) III generation cephalosporins or their enzyme-inhibitor combinations, carbapenems such as cephamycins, oxacephems, ertapenem; (3) monotherapy of the above drugs or in combination with macrolides; (4) respiratory quinolones	(1) *Enterobacteriaceae* infection must be considered in patients with underlying disease(s) and elderly patients. The patients must be further evaluated for the risk of infection with ESBLs-producing *Enterobacteriaceae*; (2) Elderly patients should be monitored for the risk factors of aspiration
Requirement for ICU admission (Intravenous administration is recommended)			
Young adults without underlying disease(s)	*S. pneumoniae*, *S. aureus*, influenza virus, adenovirus, *Legionella*	(1) Penicillins-β-lactamase-inhibitor combinations, III generation cephalosporins, cephamycins, oxacephems, ertapenem combined with macrolides; (2) respiratory quinolones	(1) *S. pneumoniae* is the most common pathogen. The other pathogens such as *S. aureus*, *Legionella*, influenza virus should also be considered (1, 2, 162-166); (2) During influenza seasons, attention must be paid to influenza viral infections. Combination with neuraminidase inhibitors should be considered. Attention should be paid to secondary *S. aureus* infection (167). The agents active against MRSA can be used in combination if necessary

For peer review only - http://bmjopen.bmj.com/site/about/guidelines.xhtml

Patients with underlying disease(s) or elderly patients (age ≥ 65 years)	*S. pneumoniae*, *Legionella*, *Enterobacteriaceae* such as *K. pneumoniae*, *S. aureus*, anaerobic bacteria, influenza virus, RSV	(1) Penicillins-β-lactamase-inhibitor combinations, III generation cephalosporins or in combination with beta-lactamase inhibitors, carbapenems such as ertapenem combined with macrolides; (2) penicillins-β-lactamase-inhibitor combinations, III generation cephalosporins or in combination with beta-lactamase inhibitors, carbapenems such as ertapenem combined with respiratory quinolones	(1) Evaluate the risk of infection with ESBLs-producing *Enterobacteriaceae*; (2) Physicians should be aware of the risk factors for aspiration and antimicrobial coverage of relevant pathogens
CAP with risk factors for *P. aeruginosa* infection and requirement for inpatient treatment or ICU admission (Intravenous administration is recommended)			
Patients with structural lung disease	*P. aeruginosa*, *S. pneumoniae*, *Legionella*, *Enterobacteriaceae* such as *K. pneumoniae*, *S. aureus*, anaerobic bacteria, influenza virus, RSV virus	(1) β-lactams with antipseudomonal activity; (2) quinolones with antipseudomonal activity; (3) β-lactams with antipseudomonal activity combined with quinolones or aminoglycosides with antipseudomonal activity; (4) combination of β-lactams, aminoglycosides and quinolones with antipseudomonal activity	Risk factors include: (1) airway *P. aeruginosa* colonization; (2) repeated doses of antibacterial drugs or glucocorticoids due to chronic airway disease. Combination therapy is recommended for patients with severe CAP or proven antimicrobial resistance

For peer review only - http://bmjopen.bmj.com/site/about/guidelines.xhtml

Appendix 6: Definition of microbiological criteria of CAP:

Definite, if one of the following criteria was met:

4. Positive urinary antigen for *Legionella pneumophila* (LP, Binax Now L pneumophila urinary antigen test; Trinity Biotech, Bray, Ireland);
5. Positive urinary antigen for *Streptococcus pneumoniae* (Binax Now *S pneumoniae* urinary antigen test; Emergo Europe, The Netherlands);
6. Positive bacterial culture from blood or plural fluid except for coagulase negative *Staphylococcus spp*.
7. Paired sera with a fourfold or more increase in the titers of antibodies to *Mycoplasma pneumoniae* (MP), *Chlamydia pneumonia*, *L pneumophila or* respiratory viruses (Influenza A and B, Parainfluenza, Adenovirus, Respiratory syncytial virus). Or Serum IgM antibody (MIF) ≥ 1:16 for *Chlamydia pneumonia.*

Probable, if one of the following criteria was met:

a. Detection of respiratory virus in sputum/bronchoalveolar lavage (BALF)/throat swabs by Realtime-PCR (Zhijiang, Shanghai, China) according to manufacturer's instructions, including respiratory syncytial virus (RSV) types A and B, influenza virus (IFV) types A and B, parainfluenza virus (PIV) types 1, 2, 3 and 4, rhinovirus (HRV), enterovirus (EV), coronavirus (hCoV) types 229E, NL63, OC43 and HKU1, parapneumovirus (hMPV), and adenovirus (AdV), bocavirus;
b. Bacteria isolated form purulent sputum (defined as an adequate quality sputum sample with > 25 leukocytes and < 10 epithelial cells per × 100 magnification field) with compatible findings of Gram staining;
c. Detection of *Mycoplasma pneumoniae* (MP), *Chlamydia pneumonia* or *L pneumophila* in sputum/BALF/throat swabs by Realtime-PCR (Zhijiang, Shanghai, China)
d. Positive antigen for Influenza A/B (Alere ™, Clearview Exact Influenza A& B)

For peer review only - http://bmjopen.bmj.com/site/about/guidelines.xhtml

e. Serum IgM antibody positive for *Mycoplasma pneumoniae* (MP), or Serum IgG antibody (MIF) ≥ 1:512 for *Chlamydia pneumonia.*

For peer review only

For peer review only - http://bmjopen.bmj.com/site/about/guidelines.xhtml

Appendix 7: CAP patients with definite and probable microbiological diagnosis

For peer review only

For peer review only - http://bmjopen.bmj.com/site/about/guidelines.xhtml

Etiology	Without risk factors for pseudomonal infection （n=409)				With risk factors for pseudomonal infection (n=329)	Total (n=738)
	age<65yr and not in ICU (n=182)	age<65yr and in ICU (n=29)	age≥65yr and not in ICU (n=162)	age≥65yr and in ICU (n=36)		
**Bacterial**	142 (19.2%)	14 (1.9%)	137 (18.6%)	34 (4.6%)	316 (42.8%)	643 (87.1%)
Pseudomonas aeruginosa	27	0	31	6	133	197
Klebsiella pneumoniae	30	9	27	10	54	130
E. coli	15	1	17	2	31	66
Acinetobacter	13	3	20	3	23	62
Staphylococcus aureus	7	3	10	7	24	51
Enterobacter cloacae	9	1	8	3	17	38
Streptococcus pneumoniae	9	1	5	1	9	25
Stenotrophomonas	8	1	10	2	4	25
Enterococcus faecalis	5	0	3	0	9	17
Enterococcus faecium	3	0	1	0	5	9
others	20	3	18	7	35	83
**Atypical etiology**	5 (0.7%)	0 (0.0%)	2 (0.3%)	0 (0.0%)	0 (0.0%)	7 (0.9%)
Mycoplasma pneumoniae	6	0	1	0	0	7
Legionnella pneumoniae	0	1	2	0	0	3
Chlamydia pneumoniae	0	1	0	0	0	1
**Virus**	30 (4.1%)	8 (1.1%)	15 (2.0%)	1 (0.1%)	9 (1.2%)	63 (8.5%)
Influenza A virus	25	8	14	1	6	54
Rhinovirus	3	2	2	0	1	8
Influenza B virus	0	0	4	1	3	8
Adenovirus	6	1	0	0	0	7
Respiratory syncytial virus	1	0	0	0	0	1
Human metapneumovirus	0	0	1	0	0	1
Cytomegalovirus	1	1	0	0	0	2
**Bacterials+viruses**	4 (0.5%)	5 (0.7%)	6 (0.8%)	1 (0.1%)	4 (0.5%)	20 (2.7%)
**Viruses+atypical pathogens**	2 (0.3%)	2 (0.3%)	1 (0.1%)	0 (0.0%)	0 (0.0%)	5 (0.7%)

For peer review only - http://bmjopen.bmj.com/site/about/guidelines.xhtml

For peer review only

For peer review only - http://bmjopen.bmj.com/site/about/guidelines.xhtml

Appendix 8: Sub-group analysis of 30-day mortality

**Item**	**30-day mortality**	**P value**
Severity of illness		
CURB-65		<0.001
0’	63 (2.7%)	
1’	93 (4.2%)	
2’	64 (7.2%)	
3’	15 (10.2%)	
4’	1 (5.0%)	
5’	1 (100.0%)	
PSI risk class		<0.001
Ⅰ	26 (2.3%)	
Ⅱ	27 (3.0%)	
Ⅲ	24 (3.2%)	
Ⅳ	45 (7.0%)	
Ⅴ	22 (12.2%)	
Age		>0.05
14~64 ys	108 (3.9%)	
65~74 ys	44 (4.1%)	
75~89 ys	81 (4.6%)	
≥90 ys	13 (7.0%)	
Gender		>0.05
Male	144 (4.6%)	
Female	102 (3.8%)	
Underlying Diseases		
None of any underlying disease	47 (2.9%)	
Chronic congestive heart failure	14 (6.9%)	<0.001

For peer review only - http://bmjopen.bmj.com/site/about/guidelines.xhtml

COPD	51 (6.4%)	<0.001
Malignant solid tumors	15 (5.9%)	<0.001
Chronic Renal diseases	11 (5.5%)	<0.001
Cerebrovascular Diseases	42 (4.7%)	<0.001
Connective Tissue Diseases	5 (4.5%)	0.003
Coronary Heart Diseases	50 (4.3%)	<0.001
Bronchiectasis	27 (4.3%)	<0.001
Hypertension	87 (4.2%)	<0.001
Asthma	14 (4.1%)	<0.001
Diabetes	36 (3.9%)	<0.001
Chronic Liver diseases	2 (2.2%)	>0.05
ICU admission		
Yes	56 (15.3%)	<0.001
No	190 (3.5%)	
Systemic glucocorticosteroids use in admission		
Yes	87 (5.6%)	<0.001
No	159 (3.7%)	

For peer review only

For peer review only - http://bmjopen.bmj.com/site/about/guidelines.xhtml

Appendix Figure 1 Patient screening algorithm for hospitalized CAP

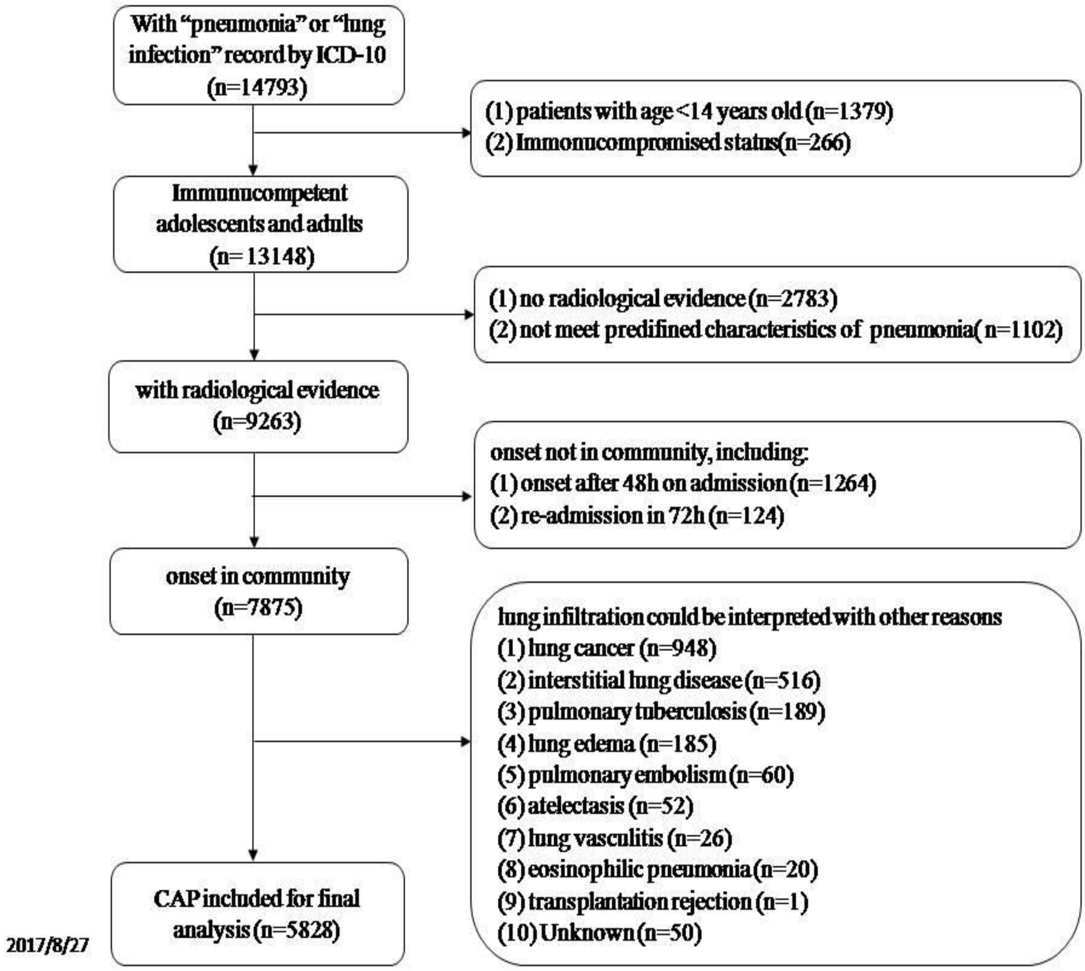

For peer review only - http://bmjopen.bmj.com/site/about/guidelines.xhtml

STROBE Statement—checklist of items that should be included in reports of observational studies

	Item No	Recommendation
**Title and abstract**	1	(*a*) Indicate the study's design with a commonly used term in the title or the abstract (Page 1)
		(*b*) Provide in the abstract an informative and balanced summary of what was done and what was found (Page 3-4)
**Introduction**		
Background/rationale	2	Explain the scientific background and rationale for the investigation being reported (Page 4-5)
Objectives	3	State specific objectives, including any prespecified hypotheses (Page 5)
**Methods**		
Study design	4	Present key elements of study design early in the paper (Page 6)
Setting	5	Describe the setting, locations, and relevant dates, including periods of recruitment, exposure, follow-up, and data collection (Page 6-9)
Participants	6	(*a*) *Cohort study*—Give the eligibility criteria, and the sources and methods of selection of participants. Describe methods of follow-up<br>*Case-control study*—Give the eligibility criteria, and the sources and methods of case ascertainment and control selection. Give the rationale for the choice of cases and controls<br>*Cross-sectional study*—Give the eligibility criteria, and the sources and methods of selection of participants (Page 6-7)
		(*b*) *Cohort study*—For matched studies, give matching criteria and number of exposed and unexposed<br>*Case-control study*—For matched studies, give matching criteria and the number of controls per case
Variables	7	Clearly define all outcomes, exposures, predictors, potential confounders, and effect modifiers. Give diagnostic criteria, if applicable (Page 7-10)
Data sources/ measurement	8*	For each variable of interest, give sources of data and details of methods of assessment (measurement). Describe comparability of assessment methods if there is more than one group (Page6- 7)
Bias	9	Describe any efforts to address potential sources of bias (Page 7)
Study size	10	Explain how the study size was arrived at (Page9)
Quantitative variables	11	Explain how quantitative variables were handled in the analyses. If applicable, describe which groupings were chosen and why (Page9-10)
Statistical methods	12	(*a*) Describe all statistical methods, including those used to control for confounding (Page 9-10)
		(*b*) Describe any methods used to examine subgroups and interactions (Page 9-10)
		(*c*) Explain how missing data were addressed (Page 9)
		(*d*) *Cohort study*—If applicable, explain how loss to follow-up was addressed<br>*Case-control study*—If applicable, explain how matching of cases and controls was addressed<br>*Cross-sectional study*—If applicable, describe analytical methods taking account of sampling strategy(Page 9-10)
		(*e*) Describe any sensitivity analyses (Page 9-10)

Continued on next page

For peer review only - http://bmjopen.bmj.com/site/about/guidelines.xhtml

**Results**		
Participants	13*	(a) Report numbers of individuals at each stage of study—eg numbers potentially eligible, examined for eligibility, confirmed eligible, included in the study, completing follow-up, and analysed (Page 10)
		(b) Give reasons for non-participation at each stage (Page 10, Appendix figure 1)
		(c) Consider use of a flow diagram (Appendix figure 1)
Descriptive data	14*	(a) Give characteristics of study participants (eg demographic, clinical, social) and information on exposures and potential confounders (Page 10-11)
		(b) Indicate number of participants with missing data for each variable of interest (Table 1-5)
		(c) *Cohort study*—Summarise follow-up time (eg, average and total amount)
Outcome data	15*	*Cohort study*—Report numbers of outcome events or summary measures over time
		Case-control study—Report numbers in each exposure category, or summary measures of exposure
		Cross-sectional study—Report numbers of outcome events or summary measures (Page 10-15)
Main results	16	(*a*) Give unadjusted estimates and, if applicable, confounder-adjusted estimates and their precision (eg, 95% confidence interval). Make clear which confounders were adjusted for and why they were included (Page 10-15)
		(*b*) Report category boundaries when continuous variables were categorized (Page 11)
		(*c*) If relevant, consider translating estimates of relative risk into absolute risk for a meaningful time period
Other analyses	17	Report other analyses done—eg analyses of subgroups and interactions, and sensitivity analyses(Appendix 8)
**Discussion**		
Key results	18	Summarise key results with reference to study objectives (Page 20-23)
Limitations	19	Discuss limitations of the study, taking into account sources of potential bias or imprecision. Discuss both direction and magnitude of any potential bias (Page 23-24)
Interpretation	20	Give a cautious overall interpretation of results considering objectives, limitations, multiplicity of analyses, results from similar studies, and other relevant evidence (Page20- 23)
Generalisability	21	Discuss the generalisability (external validity) of the study results (Page24)
**Other information**		
Funding	22	Give the source of funding and the role of the funders for the present study and, if applicable, for the original study on which the present article is based (Page25)

*Give information separately for cases and controls in case-control studies and, if applicable, for exposed and unexposed groups in cohort and cross-sectional studies.

Note: An Explanation and Elaboration article discusses each checklist item and gives methodological background and published examples of transparent reporting. The STROBE checklist is best used in conjunction with this article (freely available on the Web sites of PLoS Medicine at http://www.plosmedicine.org/, Annals of Internal Medicine at http://www.annals.org/, and Epidemiology at http://www.epidem.com/). Information on the STROBE Initiative is available at www.strobe-statement.org.

For peer review only

BMJ Open

Disease characteristics and management of hospitalized adolescents and adults with Community-Acquired Pneumonia in China：a retrospective multicenter survey

Journal:	*BMJ Open*
Manuscript ID	bmjopen-2017-018709.R2
Article Type:	Research
Date Submitted by the Author:	03-Oct-2017
Complete List of Authors:	Chen, Liang; Department of Infectious Diseases and Clinical Microbiology, Beijing Chao-Yang Hospital, Capital Medical University<br>Zhou, Fei; Department of Infectious Diseases and Clinical Microbiology, Beijing Chao-Yang Hospital, Capital Medical University<br>Li, Hui; Department of Pulmonary and Critical Care Medicine, Center for Respiratory Diseases, National Clinical Research Center of Respiratory Diseases; China-Japan Friendship Hospital<br>Xing, Xiqian; Department of Respiratory Medicine, Yan'an Hospital Affiliated to Kunming Medical University, Department of Respiratory Medicine, Yan'an Hospital Affiliated to Kunming Medical University, Kunming City, Yunnan Province<br>Han, Xiudi; Department of Respiratory Medicine, Qingdao Municipal Hospital, Qingdao City， Shandong Province<br>Wang, Yiming; Department of Pulmonary and Critical Care Medicine, Center for Respiratory Diseases, National Clinical Research Center of Respiratory Diseases; China-Japan Friendship Hospital<br>Zhang, Chunxiao; Department of Respiratory Medicine, Beijing Huimin Hospital, Beijing<br>Suo, Lijun; Department of Respiratory Medicine, Linzi District People's Hospital, Zibo City, Shandong Province<br>Wang, Jingxiang; Department of Respiratory Medicine, Beijing Luhe Hospital Affiliated to Capital Medical University, Beijing<br>Yu, Guohua; Department of Pulmonary and Critical Care Medicine, Weifang NO.2 People's Hospital, Weifang City, Shandong Province<br>Wang, Guangqiang; Department of Respiratory Medicine, Qilu Hospital Of Shandong University (Qindao), Qingdao City, Shandong Province<br>Yao, xuexin; Department of Respiratory Medicine, The 2nd Hospital of Beijing Corps, Chinese Armed Police Forces, Beijing<br>Yu, Hongxia; Department of Infectious Disease, Yantai Yuhuangding Hospital, Yantai City, Shandong Province<br>Wang, Lei; Department of Respiratory Medicine, Rizhao Chinese Medical Hospital Affiliated to Shandong Chinese Medical University, RiZhao City, Shandong Province<br>Liu, Meng; Department of Infectious Diseases and Clinical Microbiology, Beijing Chao-Yang Hospital, Capital Medical University, Beijing<br>Xue, Chunxue; Department of Infectious Diseases and Clinical Microbiology, Beijing Chao-Yang Hospital, Capital Medical University, Beijing<br>Liu, Bo; Department of Respiratory Medicine, Linzi District People's Hospital, Zibo City, Shandong Province

	Zhu, Xiaoli; Occupational Medicine and Toxicology Department, Beijing Chao-Yang Hospital, Capital Medical University, Beijing<br>Li, Yanli; Department of Infectious Diseases and Clinical Microbiology, Beijing Chao-Yang Hospital, Capital Medical University, Beijing<br>Xiao, Ying; Department of Infectious Diseases and Clinical Microbiology, Beijing Chao-Yang Hospital, Capital Medical University, Beijing<br>Cui, Xiaojing; Department of Pulmonary and Critical Care Medicine, Center for Respiratory Diseases, National Clinical Research Center of Respiratory Diseases; China-Japan Friendship Hospital<br>Li, Lijuan; Department of Pulmonary and Critical Care Medicine, Center for Respiratory Diseases, National Clinical Research Center of Respiratory Diseases; China-Japan Friendship Hospital<br>Uyeki, Timothy; Influenza Division, National Center for Immunization and Respiratory Diseases, Centers for Disease Control and Prevention-Atlanta, Georgia, USA<br>Wang, Chen; Department of Pulmonary and Critical Care Medicine, Center for Respiratory Diseases, National Clinical Research Center of Respiratory Diseases; China-Japan Friendship Hospital<br>Cao, Bin; Department of Pulmonary and Critical Care Medicine, Center for Respiratory Diseases, National Clinical Research Center of Respiratory Diseases; China-Japan Friendship Hospital
**Primary Subject Heading**:	Infectious diseases
Secondary Subject Heading:	Medical management
Keywords:	disease characteristics, management, community-acquired pneumonia, China

SCHOLARONE™ Manuscripts

For peer review only

Disease characteristics and management of hospitalized adolescents and adults with Community-Acquired Pneumonia in China：a retrospective multicenter survey

Liang Chen[1,2], Fei Zhou[1], Hui Li[3], Xiqian Xing[4], Xiudi Han[5],Yimin Wang[3], Chunxiao Zhang[6], Lijun Suo[8], Jinxiang Wang[9], Guohua Yu[11], Guangqiang Wang[12], Xuexin Yao[13], Hongxia Yu[14], Lei Wang[7], Meng Liu[1], Chunxue Xue[1], Bo Liu[8], Xiaoli Zhu[10],Yanli Li[1]， Ying Xiao[1], Xiaojing Cui[3], Lijuan Li[3], Timothy M. Uyeki[15], Chen Wang[3,16]， Bin Cao[3,16] for CAP-China network

1. Department of Infectious Diseases and Clinical Microbiology, Beijing Chao-Yang Hospital, Capital Medical University, Beijing

2. Department of Infectious Disease, 4th Medical College of Peking University, Beijing Jishuitan Hospital, Beijing

3. Department of Pulmonary and Critical Care Medicine, Center for Respiratory Diseases, National Clinical Research Center of Respiratory Diseases; China-Japan Friendship Hospital

4. Department of Respiratory Medicine, Yan'an Hospital Affiliated to Kunming Medical University, Kunming City, Yunnan Province

5. Department of Respiratory Medicine, Qingdao Municipal Hospital, Qingdao City，Shandong Province

6. Department of Respiratory Medicine, Beijing Huimin Hospital, Beijing

7. Department of Respiratory Medicine, Rizhao Chinese Medical Hospital Affiliated to Shandong Chinese Medical University, RiZhao City, Shandong Province

For peer review only

8. Department of Respiratory Medicine, Linzi District People's Hospital, Zibo City, Shandong Province

9. Department of Respiratory Medicine, Beijing Luhe Hospital Affiliated to Capital Medical University, Beijing

10. Occupational Medicine and Toxicology Department, Beijing Chao-Yang Hospital, Capital Medical University, Beijing

11. Department of Pulmonary and Critical Care Medicine, Weifang NO.2 People's Hospital, Weifang City, Shandong Province

12. Department of Respiratory Medicine, Qilu Hospital Of Shandong University (Qindao), Qingdao City, Shandong Province

13. Department of Respiratory Medicine, The 2nd Hospital of Beijing Corps, Chinese Armed Police Forces, Beijing

14. Department of Infectious Disease, YantaiYuhuangding Hospital, Yantai City, Shandong Province

15. Influenza Division, National Center for Immunization and Respiratory Diseases, Centers for Disease Control and Prevention-Atlanta, Georgia, USA

16. Department of Pulmonary Medicine; Capital Medical University, Beijing;

Corresponding Author: Dr. Bin Cao, MD. Department of Pulmonary and Critical Care Medicine，Center for Respiratory Diseases，China-Japan Friendship Hospital；National Clinical Research Center of Respiratory Diseases；Capital Medical University. Caobin_ben@vip.163.com

Abstract

For peer review only - http://bmjopen.bmj.com/site/about/guidelines.xhtml

Objectives To describe the clinical characteristics and management of patients hospitalized with CAP in China.

Design This was a multicenter, retrospective, observational study.

Setting 13 teaching hospitals in northern, central and southern China from 1 January 2014 to 31 December 2014

Participants Information on hospitalized patients aged ≥14 years with radiographically-confirmed pneumonia with illness onset in the community was collected using standard case report forms.

Primary and secondary outcome measures Resource use for CAP management.

Results Of 14,793 patients screened, 5828 with radiographically-confirmed CAP were included in the final analysis. Low mortality risk patients with a CURB-65 score 0-1 and PSI risk class Ⅰ-Ⅱ accounted for 81.2% (4434/5594) and 56.4% (2034/3609) CAP patients respectively. 21.7% (1111/5130) patients had already achieved clinical stability on admission. A definite or probable pathogen was identified only in 12.7% (738/5828) patients. 40.9% (1575/3852) patients without pseudomonal infection risk factors received antimicrobial over-treatment regimens. The median duration between clinical stability to discharge was 5.0 days with 30-day mortality of 4.2%.

Conclusions These data demonstrated overuse of health resources in CAP management, indicating that there is potential for improvement and substantial savings to health-care systems in China.

Strengths and limitations of this study

For peer review only - http://bmjopen.bmj.com/site/about/guidelines.xhtml

- This is the largest multi-center study to investigate demographic characteristics, severity and microbiological testing, empirical antimicrobial treatment, duration of hospitalization and 30-day mortality among adults and adolescents hospitalized with CAP in mainland China, including adolescents and adults of all ages admitted to general hospital wards or ICUs from the participating centers, patients who were critically ill and aged >90 years.
- The participating hospital sites are teaching hospitals in seven cities in three provinces, and may not be representative of CAP in smaller, rural hospitals.
- The majority of patients are adult CAP patients, so our findings do not apply to children hospitalized with CAP.

Background

Community acquired pneumonia (CAP) is one of the most common infectious syndromes and is a leading cause of death worldwide.[1 2] In Europe, the reported rate of CAP ranges from 1.6 to 9 cases per 1,000 in the general adult population per year.[3 4 5] Despite advances in medical technology and global economic development, CAP-associated mortality remains high (e.g., 20.9/100,000 in the United States and 12.7/100,000 in Canada).[2 6] Patients hospitalized in intensive care units for CAP have mortality in excess of 20% for immunocompetent patients and closer to 30% for those immunocompromised.[7] In Japan and Korea, the 30-day mortality of patients hospitalized with CAP is about 4-6%.[8 9]

Although mainland China has nearly 19% of the world's population, there are limited data on CAP management and disease burden in China during the last ten

For peer review only - http://bmjopen.bmj.com/site/about/guidelines.xhtml

years. According to a household interview survey published in the China Health and Family Planning Statistical Yearbook (2013), the two-week prevalence of pneumonia in China was estimated to be 11/1,000, and the direct cost due to bacterial pneumonia was about 320 million RMB (approximately $46.4 million).[10] In 2015, CAP-China, a multicenter clinical network, was founded with the support of National Key Technology Support Program from Ministry of Science and Technology (2015BAI12B11) to provide data on CAP for clinical researchers and healthcare policy makers in China.

A multicenter retrospective study of all hospitalized CAP patients from 13 centers in northern, central and southern China among CAP-China members was implemented in 2015 (Clinicaltrial Registration No.NCT02489578). To our knowledge, this is the largest multi-center study to investigate demographic characteristics, severity and microbiological testing, empirical antimicrobial treatment, duration of hospitalization and 30-day mortality among adults and adolescents hospitalized with CAP in mainland China.

Methods

Study Design and Population

Data were collected from 13 hospitals in Northern (Beijing), Central (Yantai, Qindao, Weifang, Zibo, Rizhao cities in Shandong Province) and Southern (Kunming City in Yunan Province) China. A listing of participating centers can be found in Appendix 1. All patients admitted to the 13 hospitals during 1 January 2014 through 31 December 2014 with the relevant disease codes of pneumonia or pulmonary infection in the

For peer review only - http://bmjopen.bmj.com/site/about/guidelines.xhtml

World Health Organization International Classification of Diseases 10th revision (ICD-10, Appendix 2) were eligible. Data on all eligible patients identified in screening were retrieved from the Hospital Information System (HIS) in each center. Trained physicians reviewed the medical case history and collected data on 786 variables for each patient. Chest radiographs and computerized tomography (CT) scans for each patient were reviewed by pulmonary physicians and radiologists in each center. Two-leveled review process was performed for data collection and entry.

The CAP case definition includes (1) illness onset in the community(defined as community acquired infection among those who have not been hospitalized during recent 28 days)[11]; (2) chest radiograph or CT scan showing infiltrate or interstitial changes, with or without pleural effusion; (3) any one of pneumonia clinical manifestations: (a) recent cough, sputum or aggravation of respiratory symptoms, the emergence of purulent sputum, with or without chest pain; (b) fever (defined as axillary temperature ⩾37.3°C)[12] or hypothermia (axillary temperature <36 °C); (c) signs of pulmonary consolidation and (or) moist crackles; or (d) WBC $>10\times10^9$/L, or $<4\times10^9$/L, with or without neutrophil predominance.

Patients were excluded if (1) age <14 years; (2) pneumonia onset ⩾48 hours after admission; (3) lung infiltrate or interstitial changes which were interpreted as lung cancer, pulmonary tuberculosis, non-infectious interstitial lung diseases, pulmonary edema, atelectasis, pulmonary embolism, pulmonary eosinophil infiltrate, pulmonary vasculitis; (4) immunocompromised status (including HIV(+), chemotherapy/radiotherapy within 6 months, immunosuppressive therapy, organ/bone

For peer review only - http://bmjopen.bmj.com/site/about/guidelines.xhtml

marrow transplantation, splenectomy, hematological neoplasms); (5) re-admission within 72 hours after discharge.

The study design was approved by the Ethics committee of China-Japan Friendship Hospital (No.2015-86). Given the retrospective nature of the study, the Ethics Committee determined that informed consent was not necessary.

Quality control of the study

Key investigators, including clinicians, statisticians, microbiologists and radiologists worked together to draft the protocol and created a single formatted case report form (CRF) that was utilized by all centers. Before study initiation, all investigators from the thirteen centers received training on the protocol, screening process, definition of underlying diseases and formatted CRF（Appendix file 3). After data were collected, the CRF was reviewed by a trained researcher to ensure its completeness and data quality. A second review was performed independently by a trained team of physicians in each center before being entering in duplicate into a computerized database.

Data Collection:

A total of 786 variables were included in the formatted CRF, including:

(1) Demographic data: age, gender, ID number, source of admission, types of medical insurance;

(2) Underlying diseases: chronic lung, heart, renal and liver diseases, diabetes, hypertension, solid organ cancers. Definition of underlying diseases is listed in Appendix file 4.

For peer review only

(3) Factors for acquisition or prevention of CAP: pregnancy, postpartum within six months, current smoking history, excessive drinking, exposure to day care center children, bed-ridden longer than two months, chronic receipt of corticosteroids (dosage equivalent prednisolone ≥10mg/d for more than 30 days), statin use, *S. pneumonia* or Influenza vaccination within one year.

(4) Clinical manifestations, clinical signs: recorded on the day of admission, on the 4th hospital day, change of antibiotics within 14 days of admission, and the day of discharge or death. Laboratory and radiological findings were also recorded if such tests were repeated by attending physicians. Pneumonia disease severity scores (PSI /CURB-65) were also recorded.

(5) Microbiological examination: Gram stain and culture of sputum within 48 hours, blood culture within 48 hours, BALF and pleural fluid culture within one week after admission, serum antibody (including IgM and IgG) for atypical pathogens (*Mycoplasma pneumoniae, Chlamydia pneumoniae, Legionella pneumophila*). Urinary antigen testing was performed for *Streptococcus pneumonia* and *Legionella spp.* Real-time PCR testing was done for respiratory virus and atypical pathogens with sputum and BALF. Nasopharyngeal (NP) swab was used for antigen testing for Influenza A and Influenza B. Aspirate was not routinely used for antigen testing.

(6) Antimicrobial treatment before admission and change of antimicrobials during hospitalization. Use of corticosteroids, vasopressors, mechanical ventilation, Continuous renal replacement therapy (CRRT) and extracorporeal membrane oxygenation (ECMO) were also recorded.

For peer review only - http://bmjopen.bmj.com/site/about/guidelines.xhtml

For peer review only

(7) Clinical stability was defined as satisfying all of the following: axillary temperature ≤37.8 °C more than 24 hours without use of antipyretic medications; resting heart rate ≤100 beats/min; respiratory rate ≤24 breaths/ minute; systolic blood pressure ≥90mmHg; SpO2 ≥90% on room air; ability to maintain oral intake; normal mental status.[13]

(8) Over-treatment was defined as: i) use of antipseudomonal β-lactams or β-lactams+ fluoquinolones in hospitalized (not in ICU) patients without risk factors for pseudomonal infection; ii) use of β-lactams (antipseudomonal or not)+ fluoquinolonesin ICU patients aged< 65yr without risk factors for pseudomonas infection；iii) use of anti-MRSA drugs in hospitalized (not in ICU) patients (Use of anti-MRSA drugs in ICU patients with MRSA risk after influenza virus infection was considered adequate).[14]

(9) Risk factors for pseudomonal infection was defined as chronic airway disease (bronchiectasis and COPD) and at least one risk factor for HCAP as defined by the 2005 IDSA/ATS adult CAP guidelines.[14 15 16 17 18]

(10) Empirical antimicrobial regimens recommended by Chinese CAP guidelines were showed in Appendix 5.

Microbiology testing

The conditions that a pathogen was defined as the definite or probable etiology based on were showed in Appendix 6.

Statistical analysis

No formal sample size calculations were performed because of the retrospective

For peer review only - http://bmjopen.bmj.com/site/about/guidelines.xhtml

descriptive study design. All data were analyzed by descriptive statistics with SPSS19. Measurement data were tested for normality by Kolmogorov-Smirnov. Measurement data of normal distribution was reported as mean ± standard deviation. Measurement data of non-normal distribution was reported as median. The $\chi 2$ test statistics were used for 30-day mortality subgroup analysis. A P-value of <0.05 was considered statistically significant.

Results

Screening Process

A total of 14,793 patients were screened to meet the inclusion and exclusion criteria for CAP and 5828 patients were included in the final analysis (Appendix Figure 1).

Epidemiological characteristics

The proportions of male and female patients were similar. The median age was 65 years, range 14-103 years. Prevalent co-morbidities included hypertension (35.2%), coronary heart disease (20.0%), diabetes (15.7%), cerebrovascular diseases (15.3%) and COPD（13.7%）. 14.9% of CAP patients had at least one healthcare associated pneumonia (HCAP) risk factor (according to IDSA/ATS HAP/HCAP guideline published in 2005[15]). 45.7% patients received antibiotics before admission.

A substantial proportion of admitted patients had relatively mild disease as indicated by the following: i) CURB-65 score[19] 0-1 accounted for 81.2%, ii) PSI risk class[20]Ⅰ~Ⅱ accounted for 56.3%; iii) Shorr Score[21] 0~1 accounts for 99.6%; and iv) Aliberti Score[22] low riskgroup in 89.7%; v) only 12.0% (261/2172) patients had procalcitonin (PCT) more than 2 ng/ml; vi) as many as 65.7% (3741/5698) patients

For peer review only - http://bmjopen.bmj.com/site/about/guidelines.xhtml

had normal peripheral leukocyte counts (4,000-10,000/ul). Most importantly, 21.7% patients had met criteria for clinical stability at hospital admission.[13] (Table 1-2)

Table 1: Demographic characteristics and underlying diseases

Items	Cases (%)
Male	3117 (53.5)
Age (years,median, IQR)	65 (53-78)
14~64	2802 (48.1)
65~74	1081 (18.5)
75~89	1760 (30.2)
≥90	185 (3.2)
Source of admission （n=5823）	
From Out-patient Department	4183 (71.8)
From Emergency Room	1588 (27.3)
Transfer from other hospital	52 (0.9)
Days from illness onset to admission (n=5826, median, IQR)	6.0 (3.0-14.0)
Patients who received antibiotics before admission	2664 (45.7）
β-lactams	1015 (38.1)
Fluoquinolones	586 (22.0)
Macrolides	170 (6.4)
β-lactams+ fluoquinolones	413 (15.5)
β-lactams+ macrolides	201 (7.5)
Others	279 (10.5)
Systemic glucocorticosteroids use before admission	250 (4.3)
Underlying Diseases	4219 (72.4)
Hypertension	2053 (35.2)
Coronary Heart Disease	1163 (20.0)
Diabetes	913 (15.7)
Cerebrovascular Diseases	890 (15.3)
COPD	801 (13.7)
Bronchiectasis	629 (10.8)
Asthma	339 (5.8)
Malignant solid tumors	254 (4.4)
Congestive Heart Failure	202 (3.5)

For peer review only - http://bmjopen.bmj.com/site/about/guidelines.xhtml

Chronic renal diseases	201 (3.4)
Connective Tissue Diseases	110 (1.9)
Chronic Hepatic Diseases	90 (1.5)
Smoking status	
Current smokers	1009 (17.3)
Ex-smokers	590 (10.1)
Alcoholism	407 (7.0)
Risk factors for aspiration*	377 (6.5)
History of CAP within one year	368 (6.3)
History of vaccination	
Influenza vaccine within 1 year	12 (0.2)
Streptococcus pneumoniae vaccine within 5 years	8 (0.1)
Risk factors for HCAP according to IDSA/ATS criteria	868 (14.9)
Hospitalized in an acute care hospital for two or more days within 90 days	404 (6.9)
Received recent intravenous antibiotic therapy, chemotherapy, or wound care within the past 30 days	656 (11.3)
Attended a hospital or hemodialysis clinic	36 (0.6)
Residence in a nursing home or long-term care facility	19 (0.3)
CURB-65 score (n=5594)	
0	2343 (41.9)
1	2199 (39.3)
2	884 (15.8)
3	147 (2.6)
4	20 (0.4)
5	1 (0.0)
PSI risk class (n=3609)	
I	1130 (31.3)
II	904 (25.0)
III	748 (20.7)
IV	646 (17.9)
V	181 (5.0)
Shorr Score (n=5650)	
0	5084 (90.0)
1	541 (9.6)

For peer review only - http://bmjopen.bmj.com/site/about/guidelines.xhtml

2	23 (0.4)
3	2 (0.0)
4	0 (0.0)
Aliberti Score（n=5828)	
Low risk group	5226 (89.7)
High risk group	602 (10.3)
Clinical stability on admission § (n=5130)	1111 (21.7)

COPD: chronic obstructive pulmonary disease; HCAP: healthcare associated pneumonia; IDSA/ATS: Infectious Diseases Society America/American Thoracic Society. PSI: pneumonia severity index. *Risk factors for aspiration included choking, drowning, nasal feeding, pseudobulbar palsy, dementia, coma, poisoning, Parkinson's disease. § Clinical stability was defined as satisfying the following at the same time: axillary temperature ≤37.8 °C more than 24 hours; heart rate ≤100 beats/min in resting state; breathing rate ≤24 breaths/minute; systolic blood pressure ≥90mmHg; SpO2 ≥90% on room air; ability to maintain oral intake; normal mental status.

Table 2: Clinical and radiological features on admission

Items	Cases（%）
Axillary Temperature≥38℃ (n=5826)	2783 (47.8)
Axillary Temperature<36℃(n=5793)	44 (0.8)
Cough	5192 (89.1)
Sputum	4751 (81.5)
Shortness of breath	2116 (36.3)
Chest pain	709 (12.2)
Decrease of consciousness	294 (5.0)
Chest signs	
Moist rales	2919 (50.1)
Dry rales	1387 (23.8)
Edema of lower limbs	592 (10.2)
Cyanosis	547 (9.4)
SBP<90 mmHg	45 (0.8)
Radiology	
Infiltrate more than two lobes	3776 (64.8)

For peer review only - http://bmjopen.bmj.com/site/about/guidelines.xhtml

Plural effusion	1205 (20.7)
Cavitation	228 (3.9)
WBC (mm^{-3}, n=5698)	
>10,000	1626 (28.5)
<4,000	331 (5.8)
4,000~10,000	3741 (65.7)
BUN >7.0 $mmol \cdot L^{-1}$ (n=5601)	1166 (20.8)
PH <7.30 (n=3330)	87 (2.6)
PaO_2/FiO_2<300 mmHg (n=3327)	1196 (35.9)
PCT ($ng \cdot ml^{-1}$, n=2172)	
PCT≤0.25	1307 (60.2)
0.25<PCT<1	479 (22.1)
1≤PCT<2	125 (5.8)
PCT≥2	261 (12.0)

SBP: systolic blood pressure; WBC: white blood cell count; BUN: blood urea nitrogen; Scr: serum creatinine; PH: potential of hydrogen; PaO_2/FiO_2: arterial pressure of oxygen/fraction of inspiration oxygen; PCT: procalcitonin.

Clinical and radiological features

Clinical and radiological features on admission are shown in Table 2. Cough, sputum, shortness of breath and fever were the most common. 64.8% patients had multi-lobar infiltrates and 20.7% of patients had pleural effusion.

Microbiological testing

75.0% patients had some types of microbiologic testing. 68.9% of patients had a sputum culture obtained within 48 hours of admission, although only 18.5% of patients were able to produce a sputum culture of acceptable quality. The proportion of patients with blood culture, BALF culture, and pleural effusion culture were 10.3%, 9.1% and 1.9% respectively. Only 0.8% of patients had a urinary antigen test sent to

For peer review only - http://bmjopen.bmj.com/site/about/guidelines.xhtml

evaluate for *Legionella pneumophila*, and 2.6% had urinary antigen testing for *Streptococcus pneumoniae*. (Table 3)

Table 3: Microbiological examination for CAP

Items	Cases (%)
Any Microbiological examination	4371 (75.0)
Microbiological examination for bacterial	4015 (68.9)
Microbiological examination for atypical etiology	1983 (34.0)
Microbiological examination for virus	2014 (34.6)
Bacterial or fungal Culture	4015 (68.9)
Qualified sputum culture*	1078 (18.5)
Blood culture **	602 (10.3)
BALF culture*[+]	532 (9.1)
Pleural effusion culture**	108 (1.9)
**Antibody-Based Assays on acute serum**	
Mycoplasma pneumoniae	IgM: 1821 (31.2)<br>IgG: 794 (13.6)
Chlamydia pneumoniae	IgM: 1294 (22.2)<br>IgG: 220 (3.8)
Legionella pneumoniae	IgM: 645 (11.1)<br>IgG: 227 (3.9)
Adenovirus	IgM: 644 (11.1)<br>IgG: 0 (0.0)
Respiratory syncytial virus	IgM: 643 (11.0)<br>IgG: 0 (0.0)
Influenza A virus	IgM: 643 (11.0)<br>IgG: 0 (0.0)
Influenza B virus	IgM: 640 (11.0)<br>IgG: 0 (0.0)
Parainfluenza virus	IgM: 643 (11.0)<br>IgG: 0 (0.0)
**Nucleic Acid-Based Molecular Diagnostics**	
From sputum	297(5.1)
Time Interval¶(days, median, IQR)	9.0 (6.0-16.0)
From BALF[+]	19 (0.3)
Time Interval¶(days, median, IQR)	13.0 (9.0-24.0)

For peer review only - http://bmjopen.bmj.com/site/about/guidelines.xhtml

Mycoplasma pneumoniae	270 (4.6)
Chlamydia spp	270 (4.6)
Legionella spp	270 (4.6)
Influenza A virus	270 (4.6)
Influenza B virus	270 (4.6)
Other respiratory virus#	270 (4.6)
**Urinary Antigen test**	
Streptococcus pneumoniae	150 (2.6)
Legionella spp	47 (0.8)
**Nasopharyngeal swab antigen testing**	
Influenza A virus	41 (0.7)
Influenza B virus	21 (0.4)

*： within 48hr after admission

**:within one week after admission

¶: days from illness onset to testing

#parainfluenza virus (PIV) types 1, 2, 3 and 4, rhinovirus (HRV), enterovirus (EV), coronovirus (hCoV) types 229E, NL63, OC43 and HKU1, parapneumovirus (hMPV), and adenovirus (AdV), bocavirus

[+]**BALF:** bronchoalveolar lavage fluid

Of all patients, serological testing for antibodies to *Mycoplasma pneumoniae* was only performed on a single serum specimen for IgM (31.2%) and IgG antibodies (13.6%). Similarly, serological testing on a single serum specimen was done for *Chlamydia pneumoniae* IgM antibody in 22.2% of patients and for IgM antibodies to *Legionella pneumophila* and respiratory viruses in 11.1%. No convalescent serum specimens were collected for serological testing for any pathogens, limiting interpretation of serology results for a single serum specimen.

A definite or probable pathogen was identified only in 12.7% of patients

For peer review only

For peer review only - http://bmjopen.bmj.com/site/about/guidelines.xhtml

(738/5828): only bacteria in 87.1% (643/738), only atypical pathogens in 0.9% (7/738), only viruses in 8.5% (63/738), bacteria and viruses in 2.7% (20/738), viruses and atypical pathogens in 0.7% (5/738). The most common five pathogens identified were *Pseudomonas aeruginosa* 26.7% (197/738), *Klebsiella pneumonia* 17.6% (130/738), *Escherichia.coli* 8.9% (66/738), *Acinetobacter* 8.4% (62/738) and influenza A virus 7.3% (54/738). (Appendix 7)

Empiric antimicrobial regimens

β-lactams (received by 72.7% of patients) and fluoquinolones (received by 42.2%) were the most common classes of antibiotics that were administered empirically. In patients (not in ICU) without pseudomonal infection risk factors, 27.8% (1070/3852) patients received empiric antibiotic regimens including antipseudomonal β-lactams, and 12.1% (468/3852) patients received β-lactams + fluoquinolones; 0.4% (16/3852) patients aged <65 years and not in ICU received β-lactams (antipseudomonal or not) + fluoquinolones combined regimens. Overall, 40.9% (1575/3852) patients without pseudomonal infection risk factors received antimicrobial over-treatment regimens.(Table 4)

Table 4: Empirical antimicrobial regimen for CAP patients (n=5716)*

**Empirical antimicrobials (%)**	**Without risk factors for *P. seudomonas* infection （n=3852）**				**With risk factors for *P. seudomonas* infection (n=1864)**
	age<65yr and not in ICU (n=1881)	age<65yr and in ICU (n=79)	age≥65yr and not in ICU (n=1742)	age≥65yr and in ICU (n=150)	
β-lactams (antipseudomonal)	178 (4.6)#	21 (0. 5)	407 (10.6)#	58 (1.5)	541 (29.0)

For peer review only - http://bmjopen.bmj.com/site/about/guidelines.xhtml

β-lactams	331 (8.6)	9 (0.2)	482 (12.5)	20 (0.5)	345 (18.5)
Fluoquinolones	502 (13.0)	10 (0.3)	273 (7.1)	6 (0.2)	252 (13.5)
Macrolides	20 (0.5)	0 (0.0)	17 (0.4)	0 (0.0)	10 (0.5)
β-lactams (antipseudomonal) + fluoquinolones	201 (5.2)[#]	13 (0.3)[#]	189 (4.9)[#]	30 (0.8)	238 (12.8)
β-lactams+ fluoquinolones	302 (7.8)[#]	3 (0.1)[#]	166 (4.3)[#]	9 (0.2)	177 (9.5)
β-lactams+ macrolides	160 (4.2)	2 (0.1)	64 (1.7)	2 (0. 1)	55 (3.0)
β-lactams (antipseudomonal) + macrolides	50 (1.3)[#]	0 (0.0)	45 (1.2)[#]	2 (0.1)	58 (3.1)
Fluoquinolones + macrolides	24 (0.6)	0 (0.0)	11 (0.3)	0 (0.0)	6 (0.3)
anti-MRSA drugs	9 (0.2)[#]	8 (0.2)	12 (0.3)[#]	6 (0.2)	29 (1.6)
Others	104 (2.7)	13 (0.3)	76 (2.0)	17 (0.4)	153 (8.2)

*: data on empirical antimicrobial regimens in 112 patients were missing.

[#]Overtreatment was defined as: i) use of antipseudomonal β -lactams or β -lactams+ fluoquinolones in hospitalized (not in ICU) patients without risk factors for pseudomonal infection; i i) use of β -lactams (antipseudomonal or not)+ fluoquinolones in ICU patients aged< 65yr without risk factors for pseudomonal infection； iii) use of anti-MRSA drugs in hospitalized (not in ICU) patients (Use of anti-MRSA drugs in ICU patients with MRSA risk after influenza virus infection was considered adequate). [14]

- Risk factors for *P. seudomonal* infection was defined as chronic airway disease (bronchiectasis or COPD) or HCAP according to IDSA/ATS criteria.[15]

Clinical outcomes

Clinical outcomes are shown in Table 5. Overall, 6.3% of patients were admitted to an ICU, and 2.7% required invasive mechanical ventilation. Vasopressors were administered to 3.4% of patients, and 26.4% received corticosteroids during the hospitalization. The 30-day mortality was 4.2%. The median duration of hospitalization was 11 days. The median duration from admission to clinical stability

For peer review only - http://bmjopen.bmj.com/site/about/guidelines.xhtml

was 4 days, and from clinical stability to discharge was 5 days. The median duration of ICU hospitalization was 8 days. The top five causes of death were severe pneumonia/multi-organ dysfunction syndrome (MODS) 69.1% (170/246), cardiac failure 2.8% (7/246), acute myocardial infarction 2.0% (5/246), stroke 1.6% (4/246) and gastrointestinal hemorrhage 1.6% (4/246).

Table 5: Supportive treatment and clinical outcomes of patients with CAP

Items	Cases （%）
ICU admission	367 (6.3)
Mechanical ventilation	
Non-invasive ventilation	286 (4.9)
Invasive ventilation in ICU	123 (2.1)
Invasive ventilation not in ICU	33 (0.6)
Vasopressor use	197 (3.4)
CRRT	16 (0.3)
ECMO	3 (0.1)
Systemic glucocorticosteroids use after diagnosis of CAP	1540 (26.4)
ICU patients who received systemicglucocorticoids	154 (2.6)
Patients on invasive mechanical ventilation who received systemic glucocorticoids	75 (1.3)
Patients on non-invasive mechanical ventilation who received systemic glucocorticoids	158 (2.7)
30-day mortality	246 (4.2)
Length of stay in Hospital (days, median,IQR)	11.0 (5.0-24.0)
Days between admission-clinical stability (median, n=5130,IQR)	4.0 (1.0-10.0)
Days between clinical stability-discharge (median, n=5130,IQR)	5.0 (1.0-9.0)
Length of stay in ICU (days, median, n=350,IQR)	8.0 (4.0-16.0)
Treatment failure within 14 days	427 (7.3)
Needs non-invasive ventilation	169 (2.9)
Needs invasive ventilation	145 (2.5)

For peer review only - http://bmjopen.bmj.com/site/about/guidelines.xhtml

Needs vasopressors	130 (2.2)
Death	147 (2.5)
Direct causes of death	
Severe pneumonia/MODS	170 (69.1)
Heart failure	7 (2.8)
Acute myocardial infarction	5 (2.0)
Stroke	4 (1.6)
Hemorrhage of digestive tract	4 (1.6)
Acute renal failure	2 (0.8)
Arhythmia	2 (0.8)
Accident aspiration	1 (0.4)
Others	51 (20.7)

ICU: intensive care unit; CRRT: continuous renal replacement therapy; ECMO: extracorporeal membrane oxygenation; MODS: multiple organ dysfunction syndrome; DIC: disseminated intravascular coagulation.

Appendix8 shows the results of sub-group analysis of 30-day mortality. Fatality increased with age. Mortality was similar between male and female patients (4.9% vs 3.5%). Mortality in patients admitted to an ICU was 15.3%.

Discussion

This study represents the largest, multicenter, retrospective cohort study on the etiologies and outcomes in adolescents and adults with CAP in China. In this study, we found that admission of patients with low mortality risk, inadequate microbiological diagnostic tests, overuse of antibiotics and incorrect serological testing for *Mycoplasma pneumoniae*, *Chlamydia pneumoniae, Legionella pneumophila* and respiratory viruses, were the main challenges of CAP management.

We identified four major categories of overuse of health care resources in CAP management in China:

For peer review only - http://bmjopen.bmj.com/site/about/guidelines.xhtml

(1) A large number of low-risk patients were admitted to the hospitals. Guidelines for CAP management in China and the U.S. recommend that decisions for hospitalization should be based on illness severity.[14 23] It was estimated that over $8 billion dollars are spent in CAP treatment every year in the U.S, and the cost for inpatient CAP management is 25-30 times more than for outpatient CAP management.[24 25 26] Therefore, admission of low mortality risk CAP patients results in major unnecessary cost expenditures. Moreover, outpatients usually return to their baseline activity levels much sooner than inpatients, and enjoyed a higher quality of life.[27 28] Finally, hospitalization is associated with the risk of nosocomial infections, potentially caused by high virulent and multidrug-resistant organisms.[29] Admission of low-risk CAP patients was also observed in a recent large U.S. study,[11] so it may not be unique to China. However, there are many other factors that play an important role in deciding the need for hospitalization such as comorbidities, lack of available family support, older age, mental illness and drug abuse, etc.[30 31]

(2) Length of stay in hospital was unnecessarily long. CAP guidelines recommend that patients should be discharged as soon as they achieve clinical stability and have no other active medical problems. Keeping patients in hospital and observing them while receiving oral antibiotic therapy, or waiting for normalization of all clinical parameters are not indicated and are associated with increased costs and potentially with in-hospital adverse events.[13 29 30] We observed that CAP patients were discharged a median of 5 days after achieving clinical stability, and 22% met clinical stability criteria at admission. Given the median LOS of 11 days for all CAP patients,

For peer review only - http://bmjopen.bmj.com/site/about/guidelines.xhtml

discharging CAP patients once they achieved clinical stability would lead to cost-savings of approximately half of the total hospitalization expenses. Similarly, the length of stay in hospital may be influenced by other social factors.

(3) 40.9% patients without risk factors for *Pseudomonal* infection received over-treatment with empiric antimicrobial regimens. Antipseudomonal β-lactams (28.2%) or β-lactams + quinolones (12.2%) were the most common empiric regimens for over-treatment. This may be due to overestimation of illness severity, clinicians unfamiliarity with CAP guidelines, or lack of microbiologic diagnostic testing. Moreover, we found quinolones use in more than 40% of CAP patients. The U.S. Food and Drug Administration (FDA) has released warnings of potential adverse effects of fluoroquinolones, such as Q-T prolongation, tendon injury, psychiatric disorder, etc.[32 33 34] As second-line anti-tuberculosis drugs, fluoroquinolones can also affect the diagnosis of tuberculosis and induce drug-resistance.[35 36]

(4) Incorrect serological testing was performed. We observed that many patients had an acute serum specimen collected for IgG serology testing for atypical bacteria and respiratory viruses without a convalescent serum specimen obtained for paired serological testing. Furthermore, many patients had testing for IgM antibodies for a variety of respiratory pathogens, but elevation of IgM antibodies with a low-normal IgG titer is uncommon during acute illness.[37 38 39] Paired serology for virus and atypical pathogens is recommended for epidemiological purpose. A follow-up convalescent serum specimen to document changes in IgG and IgM antibody levels is generally required for diagnosis.[40 41] Thus, the value of antibody testing on a single acute serum

For peer review only - http://bmjopen.bmj.com/site/about/guidelines.xhtml

specimen to determine the etiology of CAP is questionable. The costs of more frequent use of PCR testing on lower respiratory specimens may be partially offset by not performing serological testing in CAP patients.

The strengths of this study, in contrast to some past epidemiological investigations,[42] included data on bacterial isolates obtained in current clinical practice, microbiologic testing ordered, and antimicrobials administered, according to Chinese standards-of-care, and the study population included adolescents and adults of all ages admitted to general hospital wards or ICUs from the participating centers to reduce selection bias. We also included patients who were critically ill, aged >90 years and with risk factors for HCAP.

This study had several limitations. First, given the retrospective study design, it is possible that selection bias was present and the study population may not have been representative of all CAP patients admitted to the 13 participating sites. Secondly, the participating hospital sites were teaching hospitals in seven cities in three provinces, and were not selected to be representative of CAP hospital management in China, especially in smaller, rural hospitals. Third, this study reports on CAP management during 2014; analysis of multiple years of data can allow assessment of changes in CAP management. Fourth, 45.7% of CAP patients received antibiotics before hospital admission and before specimen collection, which may reduce the detection of some bacterial infections, such as *Streptococcus pneumoniae*. The low number of tests performed (good quality sputum, blood cultures, urine antigens, polymerase chain reaction) limit the knowledge of the true etiology of CAP in the study. Therefore, the

For peer review only - http://bmjopen.bmj.com/site/about/guidelines.xhtml

bacterial pathogens identified in this study may not be representative of all bacterial causes of CAP in the source patient populations for this study. Finally, while we included adolescents, the majority of patients were adult CAP patients, and our findings do not apply to children hospitalized with CAP.

In conclusion, we characterized adolescents and adults hospitalized for CAP in China and identified several problems suggesting the over-use of healthcare resources in CAP management. This suggests that education and training of clinicians on current CAP guidelines in China are needed to improve clinical management and could also result in substantial cost-saving in healthcare expenditures for CAP patients. The multi-center hospital network can serve as a platform for conducting intervention studies for hospitalized CAP patients in the future, utilizing the baseline data from this observational study.

Acknowledgments

We thank Dr. Jay Purdy (Senior Director, Anti-infectives, Pfizer Inc, 500 Arcola Rd, F3203, Collegeville, PA 19426), Francesco Blasi (Respiratory Unit, IRCCS FondazioneCa' GrandaOspedale Maggiore Policlinico, Dept of Pathophysiology and Transplantation, University of Milan, Milan, Italy) , and Richard G. Wunderink (Division of Pulmonary and Critical Care Medicine, Department of Medicine, Northwestern University Feinberg School of Medicine, Chicago, IL, USA) for his valuable comments on the manuscript.

Contributors Study design: Liang Chen, Feizhou, Hui Li, Chen Wang, Bin Cao. Data collection: Liang Chen, Fei Zhou, Hui Li, Xiqian Xing, Xiudi Han,Yimin Wang,

For peer review only - http://bmjopen.bmj.com/site/about/guidelines.xhtml

Chunxiao Zhang, Lijun Suo, Jinxiang Wang, Guohua Yu, Guangqiang Wang, Xuexin Yao, Hongxia Yu, Lei Wang, Meng Liu, Chunxue Xue, Bo Liu, Xiaoli Zhu, Yanli Li, Ying Xiao, Xiaojing Cui, Lijuan Li. Statistical analysis**:** Liang Chen, Fei Zhou, Hui Li, Xiudi Han. Writing: Liang Chen, Bin Cao, Timothy M. Uyeki. All authors take full responsibility for the study design, data analysis and interpretation, and preparation of the manuscript. All authors approved the final draft manuscript.

Funding This work was funded by National Science Grant for Distinguished Young Scholars (81425001/H0104); National Key Technology Support Program from Ministry of Science and Technology (2015BAI12B11); The Beijing Science and Technology Project (D151100002115004).

Disclaimer The views expressed are those of the authors and do not necessarily reflect the official policy of the Centers for Disease Control and Prevention.

Competing interests None declared.

Data sharing statement No additional data available

References

1. Miniño AM, Heron MP, Smith BL. Deaths: preliminary data for 2004. *Natl Vital Stat Rep* 2006;54:1-49.

2.File TM Jr, Marrie TJ. Burden of community-acquired pneumonia in North American adults. *Postgrad Med* 2010;122:130-41.

3. Almirall J, Bolíbar I, Vidal J, *et al*. Epidemiology of community-acquired pneumonia in adults: a population-based study. *Eur Respir J* 2000;15:757-63.

4. Jokinen C, Heiskanen L, Juvonen H, *et al*. Incidence of community-acquired

For peer review only - http://bmjopen.bmj.com/site/about/guidelines.xhtml

pneumonia in the population of four municipalities in eastern Finland. *Am J Epidemiol* 1993;137:977-88.

5. Woodhead MA, Macfarlane JT, McCracken JS, *et al*. Prospective study of the aetiology and outcome of pneumonia in the community. *Lancet* 1987;1:671-4.

6. Miniño AM, Heron MP, Smith BL. Deaths: preliminary data for 2004. *Natl Vital Stat Rep* 2006;54:19

7. National Center for Health Statistics (US). Health, United States, 2012: with special features on emergency care. Hyattsville, MD: National Center for Health Statistics, 2013:297-299.

8. Uematsu H, Kunisawa S, Sasaki N, *et al*. Development of a risk-adjusted in-hospital mortality prediction model for community-acquired pneumonia: a retrospective analysis using a Japanese administrative database. *BMC Pulm Med* 2014;14:203-210

9.Kim HI, Kim SW, Chang HH, *et al*. Mortality of community-acquired pneumonia in Korea: assessed with the pneumonia severity index and the CURB-65 score. *J Korean Med Sci* 2013;28:1276-82.

10. National Health and Family Planning Commission of the People's Republic of China. China health and family planning statistics yearbook 2013. (Available at: http://www.nhfpc.gov.cn/htmlfiles/zwgkzt/ptjnj/year2013/index2013.html)

11. Jain S, Self WH, Wunderink RG, *et al*. Community-Acquired Pneumonia Requiring Hospitalization among U.S. Adults. *N Engl J Med* 2015;373:415-27.

12. Zhou QT, He B, Zhu H. Potential for cost-savings in the care ofhospitalized

For peer review only - http://bmjopen.bmj.com/site/about/guidelines.xhtml

low-risk community-acquired pneumonia patients in China. *Value Health* 2009;12:40-6.

13. Ramirez JA, Vargas S, Ritter GW, *et al*. Early switch from intravenous to oral antibiotics and early hospital discharge: a prospective observational study of 200 consecutive patients with community-acquired pneumonia. *Arch Intern Med* 1999;159: 2449–54.

14. Qu JM, Cao B. Guidelines for the diagnosis and treatment of adult community acquired pneumonia in China (2016 Edition). *Zhong hua Jie He He Hu Xi Za Zhi* 2016;39:241-2

15. American Thoracic Society. Infectious Diseases Society of America.Guidelines for the management of adults with hospital-acquired, ventilator-associated, and healthcare-associated pneumonia. *Am J Respir Crit Care Med* 2005;171:388-416

16. Maruyama T, Fujisawa T, Okuno M, *et al*. A new strategy for healthcare-associated pneumonia: a 2-year prospective multicenter cohort study using risk factors for multidrug-resistant pathogens to select initial empiric therapy. *Clin Infect Dis* 2013;57:1373-83.

17. Qi F, Zhang GX, She DY *et al*. Healthcare-associated Pneumonia: Clinical Features and Retrospective Analysis Over 10 Years. *Chin Med J (Engl)* 2015;128:2707-13.

18. Seong GM, Kim M, Lee J, *et al*. Healthcare-Associated Pneumonia among Hospitalized Patients: Is It Different from Community Acquired Pneumonia? *Tuberc Respir Dis (Seoul)* 2014;76:66-74.

19. Lim WS, van der Eerden MM, Laing R, *et al*. Defining community acquired

For peer review only - http://bmjopen.bmj.com/site/about/guidelines.xhtml

pneumonia severity on presentation to hospital: an international derivation and validation study. *Thorax* 2003;58:377-82.

20. Fine MJ, Auble TE, Yealy DM, *et al*. A prediction rule to identify low-risk patients with community-acquired pneumonia. *N Engl J Med* 1997;336:243–50.

21. Shorr AF, Zilberberg MD, Reichley R, *et al*. Validation of a clinical score for assessing the risk of resistant pathogens in patients with pneumonia presenting to the emergency department. *Clin Infect Dis* 2012;54:193–8

22. Aliberti S, Di Pasquale M, Zanaboni AM, *et al*. Stratifying risk factors for multidrug-resistant pathogens in hospitalized patients coming from the community with pneumonia. *Clin Infect Dis*2012;54:470–8

23.American Thoracic Society. Infectious Disease Society / American Thoracic Society consensus guideline on the management of community-acquired pneumonia in adults. *Clin Infect Dis* 2007;44 Suppl 2:S27-72

24. Broulette J, Yu H, Pyenson B, *et al*. The Incidence Rate and Economic Burden of Community-Acquired Pneumonia in a Working-Age Population. *Am Health Drug Benefits* 2013;6:494-503.

25. Niederman MS, McCombs JS, Unger AN, *et al*. The cost of treating community-acquired pneumonia. *Clin Ther* 1998;20:820-37.

26. Rozenbaum MH, Mangen MJ, Huijts SM, *et al*. Incidence, direct costs and duration of hospitalization of patients hospitalized with community acquired pneumonia: A nationwide retrospective claims database analysis. *Vaccine* 2015;33 :3193-9.

For peer review only

27. Coley CM, Li YH, Medsger AR, *et al*. Preferences for home vs hospital care among low-risk patients with community-acquired pneumonia. *Arch Intern Med* 1996;156:1565-71

28. Carratalà J, Fernández-Sabé N, Ortega L, *et al*. Outpatient care compared with hospitalization for community-acquired pneumonia: a randomized trial inlow-risk patients. *Ann Intern Med* 2005;142:165-72.

29. Rhew DC, Riedinger MS, Sandhu M, *et al*. A prospective, multicenterstudy of a pneumonia practice guideline. *Chest* 1998;114:115–9.

30.Halm EA, Switzer GE, Mittman BS, *et al*. What factors influence physicians' decisions to switch from intravenous to oral antibiotics for community-acquired pneumonia? *J Gen Intern Med* 2001;16:599–605.

31. Goss CH, Rubenfeld GD, Park DR, *et al*. Cost and incidence of social comorbidities in low-risk patients with community-acquiredpneumonia admitted to a public hospital. *Chest* 2003;124:2148–55.

32.Aschenbrenner DS. The FDA revises Boxed Warning For Fluoroquinolones-Again. *Am J Nurs* 2016;116:22-3.

33.Tillotson GS.FDA and the safe and appropriate antibiotic use of fluoroquinolones. *Lancet Infect Dis* 2016;16:e11-2.

34. Lu ZK, Yuan J, Li M, *et al*. Cardiac risks associated with antibiotics: azithromycin and levofloxacin. *Expert Opin Drug Saf* 2015;14:295-303.

35.Rush B, Wormsbecker A, Stenstrom R, *et al*. Moxifloxacin Use and Its Association on the Diagnosis of Pulmonary Tuberculosis in An Inner City Emergency Department.

For peer review only

J Emerg Med 2016;50:371-5.

36.Mirza IA, Khan FA, Khan KA, *et al*. Extensively and pre-extensively drug resistant tuberculosis in clinical isolates of multi-drug resistant tuberculosis using classical second line (levofloxacin and amikacin). *J Coll Physicians Surg Pak* 2015;25:337-41.

37.Qu J, Gu L, Wu J, *et al*. Accuracy of IgM antibody testing, FQ-PCR and culture in laboratory diagnosis of acute infection by Mycoplasma pneumoniae in adults and adolescents with community-acquired pneumonia. *BMC Infect Dis* 2013;13:172-7.

38.Angrup A, Chaudhry R, Sharma S, *et al*. Application of real-time quantitative polymerase chain reaction assay to detect Legionella pneumophila in patients of community-acquired pneumonia in a tertiary care hospital. *Indian J Med Microbiol* 2016;34:539-543.

39.Bartlett JG.Diagnostic test for etiologic agents of community-acquired pneumonia. *Infect Dis Clin North Am* 2004;18:809-27.

40. Sayan M, Kilinç O, Yüce A, *et al*. Seropositivity against atypical pneumonia agents demonstrated in patients with community-acquired pneumonia. *Mikrobiyol Bul* 2003;37:247-53.

41.Salo P, Leinonen M. Microbiological investigations. *Semin Respir Infect*1999;14:128-34.

42.Liu YN, Chen MJ, Zhao TM, *et al*.A multicentre study on the pathogenic agentsin 665 adult patients with community-acquired pneumonia in cities of China. *Zhong hua Jie He He Hu Xi Za Zhi* 2006;29:3-8.

For peer review only

For peer review only - http://bmjopen.bmj.com/site/about/guidelines.xhtml

For peer review only

For peer review only - http://bmjopen.bmj.com/site/about/guidelines.xhtml

Appendix 1: Details of Participating centers

Name of the hospital	Province, city	2nd and 3rd level hospital	Teaching Hospital	Beds	Staff of Clinical Microbioloy Lab
Beijing Chao-Yang Hospital Affiliated to Capital Medical University	Beijing	3rd	Yes	1400	11
Beijing Jishuitan Hospital 4th Medical College of Peking University	Beijing	3rd	Yes	1500	10
Beijing Luhe Hospital Affiliated to Capital Medical University	Beijing	3rd	Yes	1042	5
Qingdao Municipal Hospital	ShanDong, Qingdao	3rd	Yes	1200	4
Qilu Hospital Of Shandong University(Qindao)	ShanDong, Jinan	3rd	Yes	1200	6
Beijing Huimin Hospital	Beijing	2nd	Yes	500	2
Linzi District People's Hospital	ShanDong, Zibo	2nd	Yes	1200	5
The 2nd Hospital of Beijing Corps, Chinese Armed Police Forces	Beijing	3rd	Yes	450	2
China-Japan Friendship Hospital	Beijing	3rd	Yes	1610	9
Yan'an Hospital Affiliated to Kunming Medical University	Kunming, Yan'an	3rd	Yes	1302	4

For peer review only

Yantai Yuhuangding Hospital	Shangdong, Yantai	3rd	Yes	3000	6
Rizhao Chinese Medical Hospital Affiliated to Shandong Chinese Medical University	Shangdong, Rizhao	3rd	Yes	1212	8
Weifang NO.2 People's Hospital	Shangdong, Weifang	3rd	Yes	1006	8

Definition of 2nd and 3rd level hospital in China:

The 2nd level hospital was defined as a hospital providing medical, prevention, health care and rehabilitation services to multiple communities (with a radius of population more than 100,000 peoples); the 3rd level hospital was defined as a hospital providing medical service to the whole country beyond cities and provinces, with comprehensive medical, teaching and research ability.

For peer review only

For peer review only - http://bmjopen.bmj.com/site/about/guidelines.xhtml

Appendix 2: ICD-10

Influenza with pneumonia, other influenza virus identified	J10.0
Influenza with pneumonia, virus not identified	J11.0
Virus pneumonia, not elsewhere classified	J12
Adenoviral pneumonia	J12.0
Respiratory syncytial virus pneumonia	J12.1
Parainfluenza virus pneumonia	J12.2
Other virus pneumonia	J12.8
Viral pneumonia,unspecified	J12.9
Pneumonia due to *Streptococcus pneumoniae*	J13
Pneumonia due to *Haemophilus influenzae*	J14
Bacterial pneumonia, not elsewhere classified	J15
Pneumonia due to *Klebsiella pneumoniae*	J15.0
Pneumonia due to *Pseudomonas spp.*	J15.1
Pneumonia due to *Staphylococcus*	J15.2
Pneumonia due to *Streptococcus spp*., group B	J15.3
Pneumonia due to other *streptococci*	J15.4
Pneumonia due to *Escherichia coli*	J15.5
Pneumonia due to other aerobic Gram-negative bacteria	J15.6
Pneumonia due to Mycoplasma pneumoniae	J15.7
Other bacterial pneumonia	J15.8

For peer review only - http://bmjopen.bmj.com/site/about/guidelines.xhtml

Bacterial pneumonia, unspecified	J15.9
Pneumonia due to other infectious organisms, not elsewhere classified	J16
Chlamydia pneumonia	J16.0
Pneumonia due to other specified infectious organisms	J16.8
Pneumonia due to other specified infectious organism	J16.8
Pneumonia in diseases classified elsewhere	J17*
Pneumonia in bacterial diseases classified elsewhere	J17.0*
Pneumonia in viral diseases classified elsewhere	J17.1*
Pneumonia in mycoses	J17.2*
Pneumonia in other diseases classified elsewhere	J17.8*
Pulmonary mycobacterial infection	A31.0
Pulmonary actinomycosis	A42.0
Pulmonary nocardiosis	A43.0
Legionnaires' disease	A48.1
Varicella pneumonia	B01.2+
Measles complicated by pneumonia	B05.1+
Cytomegaloviral pneumonitis	B25.0+
Pulmonary candidiasis	B37.1
Acute pulmonary coccidioidomycosis	B38.0
Acute pulmonary histoplasmosis capsulati	B39.0
Acute pulmonary blastomycosis	B40.0

For peer review only

For peer review only - http://bmjopen.bmj.com/site/about/guidelines.xhtml

Pulmonary paracoccidioidomycosis	B41.0
Pulmonary sporotrichosis	B42.0+
Invasive pulmonary aspergillosis	B44.0
Other pulmonary aspergillosis	B44.1
Pulmonary cryptococcosis	B45.0
Pulmonary mucormycosis	B46.0
Pneumonia, organism unspecified	J18
Bronchopneumonia, unspecified organism	J18.0
Lobar pneumonia, unspecified	J18.1
Hypostatic, pneumonia, unspecified	J18.2
Other pneumonia, organism unspecified	J18.8
Pneumonia, unspecified	J18.9

For peer review only

For peer review only - http://bmjopen.bmj.com/site/about/guidelines.xhtml

Appendix 3: Case Report Form Of Patients Hospitalized With CAP/HCAP

**Code：** **R-**	
**Name：**	**Gender：** ○**Male** ○**Female**
**Age：** ______**years old**	**Nationality：** ○**Han** ○**Others**
**Eight：** ______**cm**	**Weight：** ___ **kg**
**ID Number：**	
**Date Of Admission：** ______**Y**____**M**____**D**	
**Case Number：**	**ID Number:**
**Admission Form：** ○**Outpatience** ○**Emergency** ○**Transfers**	
**Tel：**	**Cell Phone：**
**Provider Payments：** ○**Social Medical Insurance** ○**New Rural Cooperative Medical System** ○ **Medical Services At State Expense** ○ **Commercial Medical Insurance** ○**Self-paying** ○**Others**	

Study Director：Bin Cao

Team Members：Liang Chen、Hu Li、Meng Liu、Xiudi Han、Xiaoli Zhu 、Bo Liu、Jinxiang Wang、Xuexin Yao、Chunxiao Zhang、Shujing Shi、Fei Zhou、Chunxue Xue、Yanli Li、Donghao Yu（Beijing Chao-Yang Hospital 001；Beijing Jishuitan Hospital 002；Beijing Luhe Hospital 003；Qingdao Municipal Hospital 004;Qilu Hospital Of Shandong University(Qindao) 005； Beijing Huimin Hospital 006； Linzi District People's Hospital 007； The 2nd Hospital of Beijing Corps,Chinese Armed Police Forces 008； China-Japan Friendship Hospital 009;Yan'an Hospital Affliated to Kunming Medical University 010）

For peer review only - http://bmjopen.bmj.com/site/about/guidelines.xhtml

Inclusion Criteria:

1. **Age ≥14 years old**
2. **Onset in community**
3. **Chest X-ray or CT scan showing infiltration or interstitial changes, with or without pleural effusion**
4. **Any one of pneumonia clinical manifestations, including:**

(a) Recent cough, sputum or aggravation of respiratory symptoms, the emergence of purulent sputum, with or without chest pain;

(b) Fever (axillary temperature ≥37.3°C) or hypothermia (axillary temperature <36 °C);

(c) Signs of pulmonary consolidation and (or) moist rales;

(d) WBC>10×10^9/L, or <4×10^9/L, with or without nucleus left.

Meet criteria 1,2 ,3 and anyone of criteria 4

Exclusion Criteria:

1. **Lung infiltrate or interstitial changes which can be interpreted as lung cancer, pulmonary tuberculosis, non-infectious interstitial lung diseases, pulmonary edema, atelectasis, pulmonary embolism, pulmonary eosinophil infiltration, pulmonary vasculitis;**
2. **HIV positive**
3. **Readmission within 72 hours after discharging.**

For peer review only

For peer review only - http://bmjopen.bmj.com/site/about/guidelines.xhtml

Part 1：Baseline Characteristics

Underlying Disease	
**COPD** ○Y ○N	**Asthma** ○Y ○N
**Bronchiectasis** ○Y ○N	**Malignancy** ○Y ○N
**Sleep Apnea Syndrome** ○Y ○N	**Congestive Heart Failure** ○Y ○N
**Coronary Heart Disease** ○Y ○N	**Hypertention** ○Y ○N
**Peripheral Vascular Diseases** ○Y ○ N	**Diabetes Mellitus** ○Y ○N
**Cerebrovascular Disease** ○Y ○N	**Autoimmune Diseases** [a] ○Y ○ N
**Chronic Viral Hepatitis** ○Y ○N	**Cirrhosis** ○Y ○N
**Hematological Malignancy** ○Y ○N	**Organ /bone Marrow Transplantation** ○Y ○N
**Immunosuppressive Therapy** [b] ○Y ○N	**Chemotherapy/Radiotherapy Within 6 Months** ○Y ○N
**Chronic Renal Diseases** ○Y ○N	**Splenectomy** ○Y ○N

Note： aSLE, Sjogren's syndrome, rheumatoid arthritis, polymyositis / dermatomyositis, systemic vasculitis, ankylosing spondylitis, inflammatory bowel disease, hyperthyroidism, etc;； b.Anti-rejection drugs

With The Following Situation	
**Pregnancy**	○Y ○N ○Unknown；<br>If Y，Pregnancy___weeks。
**Within 6 months after delivery**	○Y ○N ○Unknown；<br>If Y，___weeks after delivery
**Smoking**	○Y ○N ○Former Smoker ○Unknown<br>If Y，Smoked For__years，___cigarettes/day；<br>If Former Smoker，Smoked For___years，<br>___cigarettes/day ,GivenUp For______years
**Alcoholism** [a]	○Y ○N ○Unknown
**Risk factors for inhalation** [b]	○Y ○N ○Unknown
**Contact Children In Day-care Center**	○Y ○N ○Unknown
**Bed Ridden（≥2months）**	○Y ○N ○Unknown
**Long-term inhaled Corticosteroid use**[d]	○Y ○N ○Unknown
**Long-term oral Corticosteroid use**[c]	○Y ○N ○Unknown；<br>If Y，Name Of Corticosteroid：_______,

	Dose___mg/day，For______days
**Oral Statin Drugs**	○Y ○N ○Unknown
**History Of CAP Within One Year**	○Y ○N ○Unknown
**Influenza Vaccine Within 1 Year**	○Y ○N ○Unknown
***Streptococcus pneumoniae* Vaccine Within 5 Years**	○Y ○N ○Unknown

Note：**a**：drinking more than 5 bottles of beer (500ml / bottle) or half a catty liquor once in 2 weeks; or drinking more than 2.5 bottles of beer (500ml / bottle) or 2 ounc of white spirit per day for more than five years；**b:** Inhalation risk factors included choking, drowning, nasal feeding, pseudobulbar palsy, dementia, coma, poisoning, Parkinson's disease； **c:** Long-term oral corticosteroids was defined as: oral prednisone ≥10mg / d or equivalent doses of other corticosteroids for more than 3 weeks;**d**：Long-term inhaled corticosteroids was defined as: inhaled corticosteroid for more than 30 days, the daily dose wasn't limited.

**Risk Factors Of Health-Care Acquired Pneumonia**	
**Hospitalization For 2d Or More In The Preceding 90 Days**	○Y ○N ○Unknown
**Home Infusion Therapy (Including Antibiotics) Or Home Wound Care In 30 Days**	○Y ○N ○Unknown
**Chronic dialysis within 30 Days**	○Y ○N ○Unknown
**Residence In A Nursing Home Or Extended Care Facility**	○Y ○N ○Unknown

Part 2：**Data of This Hospitalization**

1.Signs And Symptoms

**History Of Present Illness**	
**Clinical Manifestation**	
**Date Of Illness Onset ： ________Y_____M_____D**	
**Fever？ （T≥37.3 ℃）**	○Y ○N；If Y ，Tmax: _____℃
**Hypothermia？ （T＜36℃）**	○Y ○N；If Y，Tmin: _____℃
**Cough？**	○Y ○N
**Sputum？**	○Y ○N；<br>If Y，○Yellow Phlegm ○White Phlegm<br>○Bloody Sputum ○Unknown
**Chest Pain？**	○Y ○N
**Shortness Of Breath？**	○Y ○N
**Sore Throat Or Rhinorrhea**	○Y ○N
**Chill/Shiver**	○Y ○N
**Exhaustion/**	○Y ○N

For peer review only - http://bmjopen.bmj.com/site/about/guidelines.xhtml

Muscle And Joint Aches//Headache	
Darrhea？	○Y ○N
Familial Aggregation （2 Epidemiological Related People Suffered From Pneumonia In Two Weeks）？	○Y ○N
Physical Examination<br>(The Worst Value Of The Day On Admission）	
Tmax，℃	
Tmin，℃	
HR, beats/min	
RR, breaths/min	
BP(Systolic Pressure / Diastolic Pressure), mmHg	
Disorder Of Consciousness？	○Y ○N
Cyanosis？	○Y ○N
Physical Signs Of Lung：	Moist rales ○Y ○N<br>Dry rales ○Y ○N
Edema Of Legs？	○Y ○N；<br>If Y，Asymmetric Edema Of Legs？ ○Y ○N

3.Pre-hospital Medical Data ○Y ○N

Radiology		
Chest X-ray<br>○Y<br>○N<br>○Unknown<br>Date of Examination：<br>____Y___M__D	Site Of Pneumonia	○Bilateral Lung ○Unilateral Lung
	Site Of Pneumonia	○Superior Lobe Of Right Lung<br>○Middle Lobe Of Right Lung<br>○Inferior Lobe Of Right Lung<br>○Superior Lobe Of Left Lung<br>○Inferior Lobe Of Left Lung<br>○Unknown
	Plural effusion	○N ○Left ○Right ○Bilateral
	Cavity	○Y ○N
	consolidation	○Y ○N
	Interstitial Change	○Y ○N
	Infiltration	○Y ○N
Lung CT<br>○Y<br>○N<br>○Unknown<br>Date of Examination：<br>____Y___M__D	Alveolar Infiltration	○Superior Lobe Of Right Lung<br>○Middle Lobe Of Right Lung<br>○Inferior Lobe Of Right Lung<br>○Superior Lobe Of Left Lung<br>○Inferior Lobe Of Left Lung<br>○Bilateral Diffuse Infiltration<br>○Unilateral Diffuse iInfiltration

For peer review only - http://bmjopen.bmj.com/site/about/guidelines.xhtml

	Plural effusion	○N ○Left ○Right ○Bilateral
	Cavity	○Y ○N
	consolidation	○Y ○N
	Abscesses	○Y ○N
	Patchy Shadow	○Y ○N
	Interstitial change	○Y ○N

Microbiological Examination

Microbiological Examination Before Admission ○Y ○N ○Unknown

If Y：
Date Of Specimen Collection：______Y_____M_____D
Specimen Type: ○Sputum ○Blood ○BALF ○Asopharyngeal Swab
○Endotracheal Aspirate ○Plural Effusion ○Urine
Microbiological Examination Results:________________________________

Treatment Before Admission

Antimicrobials Before Admission ○Y ○N ○Unknown

Drug name（Generic Name And Trade Name）	Route Of Administration	Drug Regimen	Start Time	TerminalTime
eg：Ceftriaxone（罗氏芬）	● Intravenous ○Oral	2.0g ，Qd	2014．9．1	2014．9．8
	○ Intravenous ○Oral			
	○ Intravenous ○Oral			
	○ Intravenous ○Oral			
	○ Intravenous ○Oral			
**Antiviral Drug Use Before Admission ○Y ○N ○Unknown**				
	○Intravenous ○Oral ○ Inhalation			
	○Intravenous ○Oral ○ inhalation			
**Corticosteroid Use Before Admission ○Y ○N ○Unknown**				
	○Intravenous ○Oral ○ inhalation			
	○Intravenous ○Oral ○ inhalation			

Vasopressor Use Before Admission ○Y ○N ○Unknown

If Y，Start Time：_____________ Terminal Time：_____________

Invasive Ventilation Before Admission ○Y ○N ○Unknown

If Y，Start Time：_____________ Terminal Time：_____________

For peer review only - http://bmjopen.bmj.com/site/about/guidelines.xhtml

Note:b.Vasopressors：Norepinephrine、Dopamine、Dobutamine、Metaraminol、adrenaline

4.Laboratory Examination In 24hr On Admission

Category	Item	Value	Unit
Blood Routine	WBC		*10^9/L
	Neu		*10^9/L
	Lym		*10^9/L
	HGB		g/L
	HCT		%
	PLT		*10^9/L
Biochemistry	ALB		g/L
	LDH		U/L
	AST		U/L
	ALT		U/L
	ALP		U/L
	TBIL		umol/L
	DBIL		umol/L
	CK		U/L
	BUN		mmol/L
	Cr		mmol/L
	Glu		mmol/L
	K		mmol/L
	Na		mmol/L
Serum Detection	ESR		mm/h
	CRP		mg/dL
	PCT		ng/ml
	D-dimer		ng/ml
	PT		s
	APTT		s
	INR		
	BNP		pg/ml
	Ferritin		ug/l

5.Blood Gas Analysis、Radiology and Ultrasonography After Admission

Category	Item	Value

For peer review only - http://bmjopen.bmj.com/site/about/guidelines.xhtml

Blood gas analysis (The Worst Value In 24hr On Admission)	Oxygen Therapy※ ○Y ○N		○Oxygen Inhalation Through Nasal Tube_____L/min<br>○Oxygen Inhalation Through Venturi Mask______%<br>○Oxygen Inhalation Through Oxygen Masks__L/min<br>○Non-invasive Ventilation<br>○Invasive Ventilation<br>○Unknowen
	FiO_2		
	pH		
	PO_2(mmHg)		
	PCO_2(mmHg)		
	SaO_2		
	Actual Bicarbonate (mmol/l)		
	Lac (mmol/l)		
Radiology (In 24hr On Admission)	Chest X-ray ○Y ○N	Alveolar Infiltration	○Superior Lobe Of Right Lung<br>○Middle Lobe Of Right Lung<br>○Inferior Lobe Of Right Lung<br>○Superior Lobe Of Left Lung<br>○Inferior Lobe Of Left Lung<br>○Bilateral Diffuse Infiltration<br>○Unilateral Diffuse Infiltration
		Plural effusion	○N ○Left ○Right ○Bilateral
		Cavity	○Y ○N
		consolidation	○Y ○N
		Patchy Shadow	○Y ○N
		Interstitial Change	○Y ○N
	Lung CT ○Y ○N	Alveolar Infiltration	○Superior Lobe Of Right Lung<br>○Middle Lobe Of Right Lung<br>○Inferior Lobe Of Right Lung<br>○Superior Lobe Of Left Lung<br>○Inferior Lobe Of Left Lung<br>○Bilateral Diffuse Infiltration<br>○Unilateral Diffuse Infiltration
		Plural effusion	○N ○Left ○Right ○Bilateral
		Cavity	○Y ○N
		consolidation	○Y ○N
		Patchy Shadow	○Y ○N

For peer review only

For peer review only - http://bmjopen.bmj.com/site/about/guidelines.xhtml

		**Interstitial Change**	**○Y ○N**
		**Alveolar Infiltration**	**○Superior Lobe Of Right Lung<br>○Middle Lobe Of Right Lung<br>○Inferior Lobe Of Right Lung<br>○Superior Lobe Of Left Lung<br>○Inferior Lobe Of Left Lung<br>○Bilateral Diffuse Infiltration<br>○Unilateral Diffuse Infiltration**
**Ultrasonography**	**Lower Limb Vascular Ultrasound Exam**	**Venous Thrombosis**	**○N ○Left ○Right<br>○Bilateral ○ Unexamined**

Note※The Worst Value Of Blood Gas Analysis And FiO2 At That Time.

For peer review only

For peer review only - http://bmjopen.bmj.com/site/about/guidelines.xhtml

6.Keep Detailed Records Of The Following Time Points,And Write down The Code In The First Row Of The Table：

①The 4th day（The Day On Admission Is The 1st Day）；②The day of changing Antibiotics in 14 days After Admission；③The 14th day after Admission ；④The Day Of Discharging

For peer review only - http://bmjopen.bmj.com/site/about/guidelines.xhtml

Category	Item(Unit)	The Reason and The Date						
Vital Signs	Disorder Of Consciousness							
	Tmax（℃）							
	Tmin(℃)							
	HR（beats/min）							
	RR（breaths/min）							
	BP(/ mmHg）							
Symptoms<br>1. Exacerbation<br>2. Alleviation<br>3. No-change	Cough							
	Sputum							
	Chest Pain							
	Shortness Of Breath							
	Moist Rales							
	Dry Rales							
Blood Routine	WBC（*10^9/L）							
	Neu（*10^9/L）							
	Lym（*10^9/L）							
	HGB（g/L）							
	HCT（%）							
	PLT（*10^9/L）							
Biochemistry	ALB（g/L）							
	LDH（U/L）							
	AST（U/L）							
	ALT（U/L）							
	ALP（U/L）							
	TBIL（umol/L）							
	DBIL（umol/L）							
	CK（U/L）							
	CTNI（ng/ml）							
	BUN（mmol/L）							
	Cr（mmol/L）							
	[illegible]							

For peer review only - http://bmjopen.bmj.com/site/about/guidelines.xhtml

For peer review only

Note：a. Direct Microscopy of sputum is not included

For peer review only

For peer review only - http://bmjopen.bmj.com/site/about/guidelines.xhtml

7.Treatment During Hospitalization

Antibiotics Use ○Y ○N				
Drug Name （Generic Name And Trade Name）	Route Of Administration	Drug Regimen	Start Time	Terminal Ttime
eg: Ceftriaxone （罗氏芬）	● Intravenous ○Oral	2.0g，Qd	2014-3-2	2014-4-5
	○ Intravenous ○Oral			
	○ Intravenous ○Oral			
	○ Intravenous ○Oral			
	○ Intravenous ○Oral			
	○ Intravenous ○Oral			
	○ Intravenous ○Oral			
	○ Intravenous ○Oral			

Antiviral Drugs Use ○Y ○N				
Drug name （Generic Name And Trade Name）	Route Of Administration	Drug Regimen	Start Time	Terminal Time
	○ Intravenous ○Oral ○ Inhalation			
	○ Intravenous ○Oral ○ Inhalation			
	○ Intravenous ○Oral ○ Inhalation			

Glucocorticoids Use ○Y ○N				
Drug name （Generic Name And Trade Name）	Route Of Administration	Drug Regimen	Start Time	Terminal Time
	○ Intravenous ○Oral ○Inhalation			
	○ Intravenous ○Oral ○ Inhalation			

Vasopressors Use ○Y ○N				
Drug Name	Route Of Administration	Drug Regimen	Start Time	Terminal Ttime

Immunoregulation Drugs（Including Intravenous Immunoglobulin 、Thymosins）○Y ○N				
Drug Name	Route of administration	Drug Regimen	Start time	Terminal time

For peer review only - http://bmjopen.bmj.com/site/about/guidelines.xhtml

Alternative/ Supportive Treatment			
Item	Use	Start Time	Terminal Time
Continuous Venous-venous Hemofiltration	○Y ○N		
Extracorporeal Membrane Oxygenation （ECMO）	○Y ○N		
Non-invasive Ventilation	○Y ○N		
Invasive Ventilation	○Y ○N		

8、Measurement Of T Lymphocyte Subsets

Date of specimen collection: ___Y ____M ____D

T lymphocyte subsets	CD4		/ml
	CD8		/ml
	CD4%		
	CD8%		
	NK		/ml
	NKT		/ml
	CD4/CD8		

Note：Without Time Limitation

9.Microbiological Examination

(1).Microbiological Examination In 48hrs After Admission ○Y ○N

Microbiological Examination For Sputum Or eEndotracheal aAspiration<br>Date Of Specimen Collection：__Y___M___D	
Item	Results
Direct Microscopy	○Good Quality Sputum（ ＞25 leukocytes and＜10 epithelial cells per × 100 magnification field）<br>○Not Good Quality Sputum<br>○Unknown
	○G+ Cocci ○G+ Bacillus ○G- Cocci<br>○G- Bacillus ○Positive Acid-fast Stain ○None

For peer review only - http://bmjopen.bmj.com/site/about/guidelines.xhtml

Bacteria Culture	○Streptococcus pneumoniae<br>○Haemophilus influenzae<br>○Pseudomonas aeruginosa<br>○Enterobacter cloacae<br>○Acinetobacter spp<br>○Stenotrophomonas maltophilia<br>○Escherichia coli<br>○Enterococcus faecium<br>○None Or Normal oropharyngeal flora	○Moraxella catarrhalis<br>○staphylococcus aureus<br>○Klebsiella pneumoniae<br>○Proteus spp<br>○Serratia marcescens<br>○Enterobacter aerogenes<br>○Enterococcus faecalis<br>○Others: _____
	Drug Resistant Bacteria	
	○Methicillin Resistance Staphylococcus aureus（MRSA)<br>○Vancomycin-resistant Enterococcus<br>Bacteria producing ESBLs:<br>○Escherichia coli<br>○Enterobacter cloacae<br>non - fermentative bacteria.:<br>○Acinetobacter baumannii<br>○Others: ____	 <br><br>○Klebsiella pneumoniae<br>○Serratia marcescens<br><br>○Pseudomonas aeruginosa
	If Streptococcus pneumoniae ， MIC for penicillin____ug/ml;<br>○Not detected	
	If MRSA， MIC for Vancomycin_____ug/ml;<br>○Not detected	
Direct Microscopy	○Fungal Spore<br>○Cryptococcus neoformans	○Fungal Hyphae<br>○None
Fungi Culture	○Spergillus Fumigatus<br>○Aspergillus terreus<br>○Candida Spp<br>○Undetected	○Aspergilusflavus<br>○Mucor Mucedo<br>○Cryptococcus Neoformans<br>○Others: ____
Nucleic Acid Test For Respiratory Virus	○Influenza A H1N1<br>○Influenza A H2N3<br>○Nontypeable Influenza A<br>○Adenovirus<br>○Parainfluenza virus2<br>○Parainfluenza virus 4<br>○Rhinovirus<br>○Coronavirus OC43HKU1<br>○Coronavirus 229ENL63<br>○Bocavirus<br>○EB virus	○Avian influenza H7N9<br>○Influenza A H5N1<br>○Influenza B<br>○Parainfluenza virus 1<br>○Parainfluenza virus 3<br>○Respiratory syncytial virus A<br>○Respiratory syncytial virus B<br>○Enterovirus<br>○Herpes simplex virus<br>○Cytomegalovirus<br>○MERS-CoV

For peer review only - http://bmjopen.bmj.com/site/about/guidelines.xhtml

Nucleic Acid Test For Atypical Etiology	○Mycoplasma pneumoniae ○Chlamydia pneumoniae<br>○Legionella spp

(2).Microbiological Examination For BALF? ○Y ○N

Microbiological examination for BALF（Within One Week After Admission）<br>Date Of Specimen Collection： __Y___M___D	
Item	Results
Direct Microscopy	○G+ Cocci ○G+ Bacillus ○G- Cocci<br>○G- Bacillus ○Positive Acid-fast Stain ○None
Bacteria Culture	○Streptococcus pneumoniae ○Moraxella catarrhalis<br>○Haemophilus influenzae ○staphylococcus aureus<br>○Pseudomonas aeruginosa ○Klebsiella pneumoniae<br>○Enterobacter cloacae ○Proteus spp<br>○Acinetobacter spp ○Serratia marcescens<br>○Stenotrophomonas maltophilia ○Enterobacter aerogenes<br>○Escherichia coli ○Enterococcus faecalis<br>○Enterococcus faecium ○Others： _____<br>○None Or Normal oropharyngeal flora
	Drug Resistant Bacteria
	○Methicillin Resistance Staphylococcus aureus（MRSA)<br>○Vancomycin-resistant Enterococcus<br>Bacteria producing ESBLs：<br>○Escherichia coli ○Klebsiella pneumoniae<br>○Enterobacter cloacae ○Serratia marcescens<br>non - fermentative bacteria.：<br>○Acinetobacter baumannii ○Pseudomonas aeruginosa<br>○Others： ____
	If Streptococcus pneumoniae ， MIC for penicillin____ug/ml；<br>○Not Detected
	If MRSA， MIC for Vancomycin_____ug/ml；<br>○Not Detected
Direct Microscopy	○Fungal Spore ○Fungal Hyphae<br>○Cryptococcus neoformans ○None

For peer review only - http://bmjopen.bmj.com/site/about/guidelines.xhtml

**Fungi Culture**	**○Spergillus Fumigatus**<br>**○Aspergillus terreus**<br>**○Candida Spp**<br>**○Undetected**	**○Aspergilusflavus**<br>**○Mucor Mucedo**<br>**○Cryptococcus Neoformans**<br>**○Others: ____**
**Nucleic Acid For Respiratory Virus**	**○Influenza A H1N1**<br>**○Influenza A H2N3**<br>**○Nontypeable Influenza A**<br>**○Adenovirus**<br>**○Parainfluenza virus2**<br>**○Parainfluenza virus 4**<br>**○Rhinovirus**<br>**○Coronavirus OC43HKU1**<br>**○Coronavirus 229ENL63**<br>**○Bocavirus**<br>**○EB virus**	**○Avian influenza H7N9**<br>**○Influenza A H5N1**<br>**○Influenza B**<br>**○Parainfluenza virus 1**<br>**○Parainfluenza virus 3**<br>**○Respiratory syncytial virus A**<br>**○Respiratory syncytial virus B**<br>**○Enterovirus**<br>**○Herpes simplex virus**<br>**○Cytomegalovirus**<br>**○MERS-CoV**
**Nucleic Acid Test For Atypical Etiology**	○Mycoplasma pneumoniae<br>○Legionella spp	**○Chlamydia pneumoniae**

(3).Blood Culture In One Week After Admission? ○Y ○N

**Blood Culture (In One Week After Admission)<br>Date Of Specimen Collection: __Y___M___D**		
**Item**	**Results**	
**Bacteria Culture**	**○Staphylococcus aureus**<br>**○Haemophilus influenzae**<br>**○Klebsiella pneumoniae**<br>**○Proteus spp**<br>**○Serratia marcescens**<br>**○Enterobacter aerogenes**<br>**○Enterococcus faecalis**<br>**○Others: ___________**	**○Moraxella catarrhalis**<br>**○Pseudomonas aeruginosa**<br>**○Enterobacter cloacae**<br>**○Acinetobacter spp**<br>**○Stenotrophomonas maltophilia**<br>**○Escherichia coli**<br>**○Enterococcus faecium**<br>**○None or normal oropharyngeal flora**
	**Drug Resistant Bacteria**	
	**○Methicillin resistance staphylococcus aureus（MRSA)**<br>**○Vancomycin-resistant Enterococcus**<br>**Bacteria producing ESBLs:**<br>**○Escherichia coli** ○Klebsiella pneumoniae<br>**○Enterobacter cloacae** ○Serratia marcescens<br>**non - fermentative bacteria.:**<br>**○Acinetobacter baumannii** ○Pseudomonas aeruginosa<br>**○Others: ____**	

For peer review only - http://bmjopen.bmj.com/site/about/guidelines.xhtml

	If Streptococcus pneumoniae ， MIC for penicillin____ug/ml；<br>○Not Detected
	If MRSA， MIC for Vancomycin_____ug/ml；<br>○Not Detected
Fungi Culture	○Candidiasis albicans ○Candida krusei<br>○Candida tropicalis ○Candida glabrata<br>○Candida parapsilosis ○Cryptococcus neoformans<br>○Aspergillus fumigatus ○Aspergilus flavus<br>○Aspergillus terreus ○Mucor Mucedo<br>○Undetected ○Others： ____

（4）、Pleural Effusion？ ○Y ○N

Pleural Effusion Test？ ○Y ○N

Microbiological Examination For Pleural Effusion（Without Time Limitation）<br>Date of Specimen collection： __Y___M___D	
Pleural Effusion Routine	
Total Cell Count： ___×10⁶/L； Multinuclear Cell： ___×10⁶/L；<br>Mononuclear Cells： ___×10⁶/L	
Pleural Effusion Biochemistry	
LDH： ___U/L； ADA： ___U/L； Pr： ___g/L<br>Glu:___mmol/L Cl： ___mmol/L	
Item	Results
Bacteria Culture	○Staphylococcus aureus ○Moraxella catarrhalis<br>○Haemophilus influenzae ○Pseudomonas aeruginosa<br>○Klebsiella pneumoniae ○Enterobacter cloacae<br>○Proteus spp ○Acinetobacter spp<br>○Serratia marcescens ○Stenotrophomonas maltophilia<br>○Enterobacter aerogenes ○Escherichia coli<br>○Enterococcus faecalis ○Enterococcus faecium<br>○Others: ___________ ○None or Normal Oropharyngeal Flora
	Drug Resistant Bacteria
	○Methicillin resistance staphylococcus aureus（MRSA)<br>○Vancomycin-resistant Enterococcus<br>Bacteria producing ESBLs：<br>○Escherichia coli ○Klebsiella pneumoniae<br>○Enterobacter cloacae ○Serratia marcescens<br>non - fermentative bacteria.:<br>○Acinetobacter baumannii ○Pseudomonas aeruginosa<br>○Others: ____
Fungi Culture	○Candidiasis albicans ○Candida krusei<br>○Candida tropicalis ○Candida glabrata

For peer review only - http://bmjopen.bmj.com/site/about/guidelines.xhtml

	○Candida parapsilosis	○Cryptococcus neoformans
	○Aspergillus fumigatus	○Aspergilus flavus
	○Aspergillus terreus	○Mucor Mucedo
	○Undetected	○Others: _

（5）、Antigen Test In 48hr After Admission？ ○Y ○N

Urinary antigen（in 48hr after admission）<br>Date of specimen collection: __Y___M___D	
Urinary Antigen For *Legionella spp*	○Positive ○Negative ○Undetected
Urinary Antigen For *Streptococcus pneumoniae*	○Positive ○Negative ○Undetected
Throat Swab Aantigen Test（In 48hr After Admission）<br>Date Of Specimen Collection: __Y___M___D	
Respiratory Syncytial Virus Antigen Test	○Positive ○Negative ○Undetected
Influenza A Antigen Test	○Positive ○Negative ○Undetected
Influenza B Antigen Test	○Positive ○Negative ○Undetected

（6）、Antibody Test？ a）○Y ○N

b）If Y , Titer Of Antibody In Paired Serum? ○Y ,Interval___days

○N

Antibody Test（Without Time Limitation）<br>Date Of Specimen Collection: __Y___M___D	
○IgM for *Mycoplasmal pneumonia*	○IgM for Influenza A
○IgG for *Mycoplasmal pneumonia*	○IgM for Parainfluenza
○IgM for *Chlamydia* spp	○IgM for Q fever
○IgG for *Chlamydia* spp.	○IgM for Adenovirus
○IgM for *Legionella spp*	○IgM for Respiratory syncytial virus
○IgG for *Legionella spp*	○IgM for Parainfluenza 1,2,3

For peer review only - http://bmjopen.bmj.com/site/about/guidelines.xhtml

10、Outcomes

(1).Treatment Failure Within 14 Days

**Treatment Failure Within 14 Days（Multiple choices）**<br>（The Value Of The 1st Day On Admission As The Baseline Data）	
**1.Needs Invasive Ventilation**	**○Y ○N**
**2.Needs Non-invasive Ventilation**	**○Y ○N**
**3.Needs Vasopressors**	**○Y ○N**
**4.Death**	**○Y ○N**

**The Reasons For Treatment Failure**			
**1.CAP Progression**	**Pneumonia Progression**	**○Y**	**○N**
**2.CAP Complications**	**Pyothorax**	**○Y**	**○N**
	**Endocarditis**	**○Y**	**○N**
	**Meningitis**	**○Y**	**○N**
	**Others: ____________**		
**3.Severe Sepsis Due To CAP**	**ARDS**	**○Y**	**○N**
	**Sepsis**	**○Y**	**○N**
	**Hepatic Failure**	**○Y**	**○N**
	**Renal Ffailure**	**○Y**	**○N**
	**Clotting Disorders,**	**○Y**	**○N**
	**Encephalopathy**	**○Y**	**○N**
	**Others: __________**		
**4.Complications Or Underlying Disease Deterioration**	**Pulmonary Embolism**	**○Y**	**○N**
	**Myocardial Infarction**	**○Y**	**○N**
	**Arrhythmia**	**○Y**	**○N**
	**Gastrointestinal Bleeding**	**○Y**	**○N**
	**Congestive Heart Failure**	**○Y**	**○N**
	**COPD**	**○Y**	**○N**
	**Diabetes Mellitus**	**○Y**	**○N**
	**Nephropathy**	**○Y**	**○N**
	**Others: __________**		
**5.Complications Due To Treatment**	**Hemopneumothorax**	**○Y**	**○N**
	**Allergic To Antibiotics**	**○Y**	**○N**
	**HAP/VAP**	**○Y**	**○N**
	**Vascular Catheter Infection**	**○Y**	**○N**
	**C. Difficile Infection**	**○Y**	**○N**
	**Iatrogenic Urinary Tract Infection**	**○Y**	**○N**
	**Others: __________**		
**6.Unknown**	**○Y ○N**		

For peer review only

（2）. Complications During Hospitalization

Complications During Hospitalization	
Complications（Multiple Choices） ○Y ○N	
Respiratory Failure ○Y ○N	ARDS ○Y ○N
Heart Failure ○Y ○N	Acute Myocardial Infarction ○Y ○N
Acute Liver Failure ○Y ○N	AcuteRenal Failure ○Y ○N
Septic Shock ○Y ○N	Stroke ○Y ○N
DIC ○Y ○N	Antibiotic Associated Diarrhea ○Y ○N
Arrhythmia ○Y ○N	MODS ○Y ○N
Pulmonary Embolism ○Y ○N	Deep Venous Thrombosis ○Y ○N
Ventilator Associated Pneumonia ○Y ○N	Gastrointestinal Bleeding ○Y ○N
Invasive Aspergillosis ○Y ○N	Mediastinal Emphysema ○Y ○N
Pneumothorax ○Y ○N	Nosocomial Bloodstream Infection ○Y ○N
Others ○Y ○N	If Y: ________

（3）. Outcomes

Clinical Stability Before Discharge	○Y ○N<br>If Y， the date of clinical stability ______ Y ______M ______D.<br>Meet the following seven criteria：Temperature<37.8℃ for more than 24hr； Heart rate ≤100 beats/min ； Respiratory rate ≤24 breaths/min ;Systolic blood pressure ≥90 mm Hg ； Arterial oxygen saturation ≥90% or pO2 ≥ 60 mm Hg on room air ； Ability to maintain oral intake； Normal mental status.
Admitted to RICU/ICU？	○Y ○N<br>If Y，The Date Of Admitted To RICU/ICU： ____Y____M____D<br>The Date Of Transfer From RICU/ICU： ____Y____M____D
Discharging	The Date Of Discharging____Y____M____D<br>Outcome ○Improvement ○Against-advice discharge<br>○Death<br>If death， The Death Date_____Y____M____D
Direct Cause Of Death (only one choice）	○Severe Pneumonia ○Respiratory Failure<br>○Shock○Heart Failure ○Acute Myocardial Infarction

For peer review only - http://bmjopen.bmj.com/site/about/guidelines.xhtml

	○Acute renal Failure	○Hepatic failure
	○DIC	○Stroke
	○Gastrointestinal Bleeding	○Others: ________

10. Cost And Economy Data

Total Expenses _______Yuan:

Drugs Cost: __________Yuan , Antimicrobials Cost _________Yuan

Laboratory Testing Expenses: ________Yuan

Bed Charge: __________Yuan

Health Care Worker Labor Cost: _________Yuan

For peer review only

For peer review only - http://bmjopen.bmj.com/site/about/guidelines.xhtml

Appendix 4: Definition of underlying diseases

1) Long-term smoking was defined as: cigarette smokers of 10 cigarettes/d during at least the previous year；

2) Alcoholism was defined as: drinking more than 5 bottles of beer (500ml / bottle) or half a catty liquor once in 2 weeks; or drinking more than 2.5 bottles of beer (500ml / bottle) or 2 ounc of white spirit per day for more than five years；

3) Long-term oral corticosteroids was defined as: oral prednisone ≥10mg / d or equivalent doses of other corticosteroids for more than 3 weeks.[1]

4) Long-term inhaled corticosteroids was defined as: inhaled corticosteroid for more than 30 days, the daily dose wasn't limited;

5) COPD was defined as: persistent airflow limitation, FEV1 / FVC < 70% post bronchodilator;

6) Asthma was defined by the history of respiratory symptoms such as wheeze, cough that varied over time and intensity, together with variable respiratory airway limitation;

7) Hypertension was defined as systolic blood pressure≥140mmHg and /or diastolic blood pressure≥90mmHg in resting status;

8) Coronary heart disease included angina pectoris, myocardial infarction, ischemic cardiomyopathy;

9) Chronic congestive heart failure was defined as cardiomegaly and ejection fraction ≤40%;

For peer review only - http://bmjopen.bmj.com/site/about/guidelines.xhtml

10) Cerebrovascular diseases included transient ischemic attack, cerebral hemorrhage, subarachnoid hemorrhage, cerebral infarction, etc;

11) Diebetes mellitus: included diabetes mellitus type 1 and diabetes mellitus type 2, not included impaired glucose tolerance and impaired fasting glycaemia;

12) Chronic liver disease included chronic viral hepatitis, chronic alcoholic liver disease, chronic fatty liver disease, etc;

13) Chronic kidney disease included diabetic nephropathy, hypertensive renal damage, chronic glomerulonephritis, chronic pyelonephritis, lupus nephritis, IgA nephropathy, nephrotic syndrome, hereditary kidney disease, etc;

14) Connective Tissue Diseases include SLE, Sjogren's syndrome, rheumatoid arthritis, polymyositis / dermatomyositis, systemic vasculitis, ankylosing spondylitis, inflammatory bowel disease, hyperthyroidism, etc;

15) Organ transplantation or bone marrow transplantation included solid organ transplantating, such as liver transplantation, kidney transplantation, lung transplantation or pancreas transplantation, etc and bone marrow transplantation;

16) Aspiration risk factors included choking, drowning, nasal, pseudobulbar palsy, dementia, coma, poisoning, Parkinson's disease.

17) Immunosuppressive therapy: was defined as systmatic glucocorticosteroid (such as prednisone ≥10mg/d for more than 3 weeks in the last month); cyclosporine or azathioprine use within 3 months, and methotrexate use ≥12.5mg/week within 3 months; biological modifiers such as etanercept and infiximab within 3 weeks.

For peer review only

18) Immunocompromised status included HIV(+），chemotherapy/radiotherapy within 6 months, immunosuppressive therapy, organ/bone marrow transplantation, splenectomy, hematological neoplasms.[2]

19) Risk factors for pseudomonal infection was defined as chronic airway disease (bronchiectasis or COPD) and HCAP risk factors according to IDSA/ATS criteria.[3-7]

20) Overtreatment was defined as: i) use of antipseudomonal β-lactams or β-lactams+ fluoquinolones in hospitalized (not in ICU) patients without risk factors for pseudomonal infection; i i) use of β-lactams(antipseudomonal or not)+ fluoquinolones in ICU patients aged< 65yr without risk factors for pseudomonas infection；iii) use of anti-MRSA drugs in hospitalized (not in ICU) patients.[3]

References:

1. Jouneau S, Poineuf JS, Minjolle S, *et al*. Which patients should be tested for viruses on bronchoalveolar lavage fluid? *Eur J Clin Microbiol Infect Dis* 2013 ;32:671-7.

2. Sousa D, Justo I, Domínguez A,*et al*. Community-acquired pneumonia in immunocompromised older patients: incidence, causative organisms and outcome. *Clin Microbiol Infect* 2013 ;19:187-92

3. Guidelines for the diagnosis and treatment of adult community-acquired pneumonia in China(2016 Edition). *Zhonghua Jie He He Hu Xi Za Zhi* 2016; 39: 241-2

4. American Thoracic Society.Infectious Diseases Society of America.Guidelines for the management of adults with hospital-acquired, ventilator-associated, and healthcare-associated pneumonia.*Am J Respir Crit Care Med* 2005; 171:388-416

For peer review only - http://bmjopen.bmj.com/site/about/guidelines.xhtml

5. Maruyama T1, Fujisawa T, Okuno M, *et al*. A new strategy for healthcare-associated pneumonia: a 2-year prospective multicenter cohort study using risk factors for multidrug-resistant pathogens to select initial empiric therapy. *Clin Infect Dis* 2013;57:1373-83.

6. Qi F, Zhang GX, She DY *et al*. Healthcare-associated Pneumonia: Clinical Features and Retrospective Analysis Over 10 Years. *Chin Med J (Engl)* 2015;128:2707-13.

7. Seong GM, Kim M, Lee J, *et al*. Healthcare-Associated Pneumonia among Hospitalized Patients: Is It Different from Community Acquired Pneumonia? *Tuberc Respir Dis (Seoul)* 2014;76:66-74.

For peer review only

Appendix 5 Empirical antimicrobial regimens according to Chinese CAP guideline

Populations	Common pathogens	Anti-infective agents for initial empirical therapy	Comment
Outpatient treatment (Oral administration is recommended)			
Young adults without underlying disease(s)	*S. pneumoniae*, *M. pneumoniae*, *H. influenzae*, *C. pneumoniae*, influenza virus, adenovirus, *M. catarrhalis*	(1) Aminopenicillins, penicillins-β-lactamase -inhibitor combinations; (2) I or II generation cephalosporins; (3) doxycycline or minocycline; (4) respiratory quinolones; (5) macrolides	(1) Differentiate among bacterial pneumonia, *Mycoplasma*, *Chlamydia* and viral pneumonia based on clinical characteristics; (2) Mild pneumonia caused by *Mycoplasma*, *Chlamydia*, and virus is usually self-limited
Patients with underlying disease(s) or elderly patients (age ≥ 65 years)	*S. pneumoniae*, *H. influenzae*, *Enterobacteriaceae* such as *K. pneumoniae*, *C. pneumoniae*, influenza virus, RSV, *M. catarrhalis*	(1) Penicillins-β-lactamase-inhibitor combinations; (2) II or III generation cephalosporins (oral); (3) respiratory quinolones; (4) penicillins-lactamase -inhibitor combinations, II generation cephalosporins, III generation cephalosporins combined with doxycycline or minocycline or macrolides	Monotherapy with doxycycline or minocycline or macrolides is not recommended in patients with risk factors of resistant *S. pneumoniae* (1), such as age > 65 years, underlying diseases (chronic cardiac, pulmonary, or renal diseases, diabetes mellitus, and immunosuppression), alcoholism, and β-lactams treatment within 3 months.
Inpatient treatment, non-ICU (Intravenous or oral administration)			

For peer review only - http://bmjopen.bmj.com/site/about/guidelines.xhtml

Young adults without underlying disease(s)	*S. pneumoniae*, *H. influenzae*, *M. catarrhalis*, *S. aureus*, *M. pneumoniae*, *C. pneumoniae*, influenza virus, adenovirus, other respiratory tract viruses	(1) Penicillin G, aminopenicillins, penicillins-β-lactamase-inhibitor combinations; (2) II or III generation cephalosporins, cephamycins, oxacephems; (3) the above drugs combined with doxycycline, minocycline or macrolides; (4) respiratory quinolones; (5) macrolides	(1) Only 1.9% the *S. pneumoniae* isolates from adult CAP are resistant to intravenous penicillins in China. The percentage of intermediate strains is only about 9%. Intravenous penicillins are still effective in hospitalized patients infected with penicillin-intermediate *S. pneumoniae* when increasing the dosage (23, 161); (2) When atypical pathogens are suspected, doxycycline or minocycline or respiratory quinolones are preferred. Macrolides can be used in regions with lower resistance rate to mycoplasma
Patients with underlying disease(s) or elderly patients (age ≥ 65 years)	*S. pneumoniae*, *H. influenzae*, *Enterobacteriaceae* such as *K. pneumoniae*, influenza virus, RSV, *M. catarrhalis*, anaerobic bacteria, *Legionella*	(1) Penicillins-β-lactamase-inhibitor combinations; (2) III generation cephalosporins or their enzyme-inhibitor combinations, carbapenems such as cephamycins, oxacephems, ertapenem; (3) monotherapy of the above drugs or in combination with macrolides; (4) respiratory quinolones	(1) *Enterobacteriaceae* infection must be considered in patients with underlying disease(s) and elderly patients. The patients must be further evaluated for the risk of infection with ESBLs-producing *Enterobacteriaceae*; (2) Elderly patients should be monitored for the risk factors of aspiration
Requirement for ICU admission (Intravenous administration is recommended)			
Young adults without underlying disease(s)	*S. pneumoniae*, *S. aureus*, influenza virus, adenovirus, *Legionella*	(1) Penicillins-β-lactamase-inhibitor combinations, III generation cephalosporins, cephamycins, oxacephems, ertapenem combined with macrolides; (2) respiratory quinolones	(1) *S. pneumoniae* is the most common pathogen. The other pathogens such as *S. aureus*, *Legionella*, influenza virus should also be considered (1, 2, 162-166); (2) During influenza seasons, attention must be paid to influenza viral infections. Combination with neuraminidase inhibitors should be considered. Attention should be paid to secondary *S. aureus* infection (167). The agents active against MRSA can be used in combination if necessary

For peer review only

For peer review only - http://bmjopen.bmj.com/site/about/guidelines.xhtml

Patients with underlying disease(s) or elderly patients (age ≥ 65 years)	*S. pneumoniae*, *Legionella*, *Enterobacteriaceae* such as *K. pneumoniae*, *S. aureus*, anaerobic bacteria, influenza virus, RSV	(1) Penicillins-β-lactamase-inhibitor combinations, III generation cephalosporins or in combination with beta-lactamase inhibitors, carbapenems such as ertapenem combined with macrolides; (2) penicillins-β-lactamase-inhibitor combinations, III generation cephalosporins or in combination with beta-lactamase inhibitors, carbapenems such as ertapenem combined with respiratory quinolones	(1) Evaluate the risk of infection with ESBLs-producing *Enterobacteriaceae*; (2) Physicians should be aware of the risk factors for aspiration and antimicrobial coverage of relevant pathogens
CAP with risk factors for *P. aeruginosa* infection and requirement for inpatient treatment or ICU admission (Intravenous administration is recommended)			
Patients with structural lung disease	*P. aeruginosa*, *S. pneumoniae*, *Legionella*, *Enterobacteriaceae* such as *K. pneumoniae*, *S. aureus*, anaerobic bacteria, influenza virus, RSV virus	(1) β-lactams with antipseudomonal activity; (2) quinolones with antipseudomonal activity; (3) β-lactams with antipseudomonal activity combined with quinolones or aminoglycosides with antipseudomonal activity; (4) combination of β-lactams, aminoglycosides and quinolones with antipseudomonal activity	Risk factors include: (1) airway *P. aeruginosa* colonization; (2) repeated doses of antibacterial drugs or glucocorticoids due to chronic airway disease. Combination therapy is recommended for patients with severe CAP or proven antimicrobial resistance

For peer review only

For peer review only - http://bmjopen.bmj.com/site/about/guidelines.xhtml

Appendix 6: Definition of microbiological criteria of CAP:

Definite, if one of the following criteria was met:

4. Positive urinary antigen for *Legionella pneumophila* (LP, Binax Now L pneumophila urinary antigen test; Trinity Biotech, Bray, Ireland);
5. Positive urinary antigen for *Streptococcus pneumoniae* (Binax Now *S pneumoniae* urinary antigen test; Emergo Europe, The Netherlands);
6. Positive bacterial culture from blood or plural fluid except for coagulase negative *Staphylococcus spp*.
7. Paired sera with a fourfold or more increase in the titers of antibodies to *Mycoplasma pneumoniae* (MP), *Chlamydia pneumonia*, *L pneumophila or* respiratory viruses (Influenza A and B, Parainfluenza, Adenovirus, Respiratory syncytial virus). Or Serum IgM antibody (MIF) ≥ 1:16 for *Chlamydia pneumonia.*

Probable, if one of the following criteria was met:

a. Detection of respiratory virus in sputum/bronchoalveolar lavage (BALF)/throat swabs by Realtime-PCR (Zhijiang, Shanghai, China) according to manufacturer's instructions, including respiratory syncytial virus (RSV) types A and B, influenza virus (IFV) types A and B, parainfluenza virus (PIV) types 1, 2, 3 and 4, rhinovirus (HRV), enterovirus (EV), coronavirus (hCoV) types 229E, NL63, OC43 and HKU1, parapneumovirus (hMPV), and adenovirus (AdV), bocavirus;
b. Bacteria isolated form purulent sputum (defined as an adequate quality sputum sample with > 25 leukocytes and < 10 epithelial cells per × 100 magnification field) with compatible findings of Gram staining;
c. Detection of *Mycoplasma pneumoniae* (MP), *Chlamydia pneumonia* or *L pneumophila* in sputum/BALF/throat swabs by Realtime-PCR (Zhijiang, Shanghai, China)
d. Positive antigen for Influenza A/B (Alere ™, Clearview Exact Influenza A& B)

For peer review only - http://bmjopen.bmj.com/site/about/guidelines.xhtml

e. Serum IgM antibody positive for *Mycoplasma pneumoniae* (MP), or Serum IgG antibody (MIF) ≥ 1:512 for *Chlamydia pneumonia.*

For peer review only

For peer review only - http://bmjopen.bmj.com/site/about/guidelines.xhtml

Appendix 7: CAP patients with definite and probable microbiological diagnosis

For peer review only

For peer review only - http://bmjopen.bmj.com/site/about/guidelines.xhtml

Etiology	Without risk factors for pseudomonal infection （n=409)				With risk factors for pseudomonal infection (n=329)	Total (n=738)
	age<65yr and not in ICU (n=182)	age<65yr and in ICU (n=29)	age≥65yr and not in ICU (n=162)	age≥65yr and in ICU (n=36)		
**Bacterial**	142 (19.2%)	14 (1.9%)	137 (18.6%)	34 (4.6%)	316 (42.8%)	643 (87.1%)
Pseudomonas aeruginosa	27	0	31	6	133	197
Klebsiella pneumoniae	30	9	27	10	54	130
E. coli	15	1	17	2	31	66
Acinetobacter	13	3	20	3	23	62
Staphylococcus aureus	7	3	10	7	24	51
Enterobacter cloacae	9	1	8	3	17	38
Streptococcus pneumoniae	9	1	5	1	9	25
Stenotrophomonas	8	1	10	2	4	25
Enterococcus faecalis	5	0	3	0	9	17
Enterococcus faecium	3	0	1	0	5	9
others	20	3	18	7	35	83
**Atypical etiology**	5 (0.7%)	0 (0.0%)	2 (0.3%)	0 (0.0%)	0 (0.0%)	7 (0.9%)
Mycoplasma pneumoniae	6	0	1	0	0	7
Legionnella pneumoniae	0	1	2	0	0	3
Chlamydia pneumoniae	0	1	0	0	0	1
**Virus**	30 (4.1%)	8 (1.1%)	15 (2.0%)	1 (0.1%)	9 (1.2%)	63 (8.5%)
Influenza A virus	25	8	14	1	6	54
Rhinovirus	3	2	2	0	1	8
Influenza B virus	0	0	4	1	3	8
Adenovirus	6	1	0	0	0	7
Respiratory syncytial virus	1	0	0	0	0	1
Human metapneumovirus	0	0	1	0	0	1
Cytomegalovirus	1	1	0	0	0	2
**Bacterials+viruses**	4 (0.5%)	5 (0.7%)	6 (0.8%)	1 (0.1%)	4 (0.5%)	20 (2.7%)
**Viruses+atypical pathogens**	2 (0.3%)	2 (0.3%)	1 (0.1%)	0 (0.0%)	0 (0.0%)	5 (0.7%)

For peer review only

For peer review only - http://bmjopen.bmj.com/site/about/guidelines.xhtml

For peer review only

For peer review only - http://bmjopen.bmj.com/site/about/guidelines.xhtml

Appendix 8: Sub-group analysis of 30-day mortality

Item	30-day mortality	P value
Severity of illness		
CURB-65		<0.001
0’	63 (2.7%)	
1’	93 (4.2%)	
2’	64 (7.2%)	
3’	15 (10.2%)	
4’	1 (5.0%)	
5’	1 (100.0%)	
PSI risk class		<0.001
Ⅰ	26 (2.3%)	
Ⅱ	27 (3.0%)	
Ⅲ	24 (3.2%)	
Ⅳ	45 (7.0%)	
Ⅴ	22 (12.2%)	
Age		>0.05
14~64 ys	108 (3.9%)	
65~74 ys	44 (4.1%)	
75~89 ys	81 (4.6%)	
≥90 ys	13 (7.0%)	
Gender		>0.05
Male	144 (4.6%)	
Female	102 (3.8%)	
Underlying Diseases		
None of any underlying disease	47 (2.9%)	
Chronic congestive heart failure	14 (6.9%)	<0.001

For peer review only - http://bmjopen.bmj.com/site/about/guidelines.xhtml

COPD	51 (6.4%)	<0.001
Malignant solid tumors	15 (5.9%)	<0.001
Chronic Renal diseases	11 (5.5%)	<0.001
Cerebrovascular Diseases	42 (4.7%)	<0.001
Connective Tissue Diseases	5 (4.5%)	0.003
Coronary Heart Diseases	50 (4.3%)	<0.001
Bronchiectasis	27 (4.3%)	<0.001
Hypertension	87 (4.2%)	<0.001
Asthma	14 (4.1%)	<0.001
Diabetes	36 (3.9%)	<0.001
Chronic Liver diseases	2 (2.2%)	>0.05
ICU admission		
Yes	56 (15.3%)	<0.001
No	190 (3.5%)	
Systemic glucocorticosteroids use in admission		
Yes	87 (5.6%)	<0.001
No	159 (3.7%)	

For peer review only

Appendix Figure 1 Patient screening algorithm for hospitalized CAP

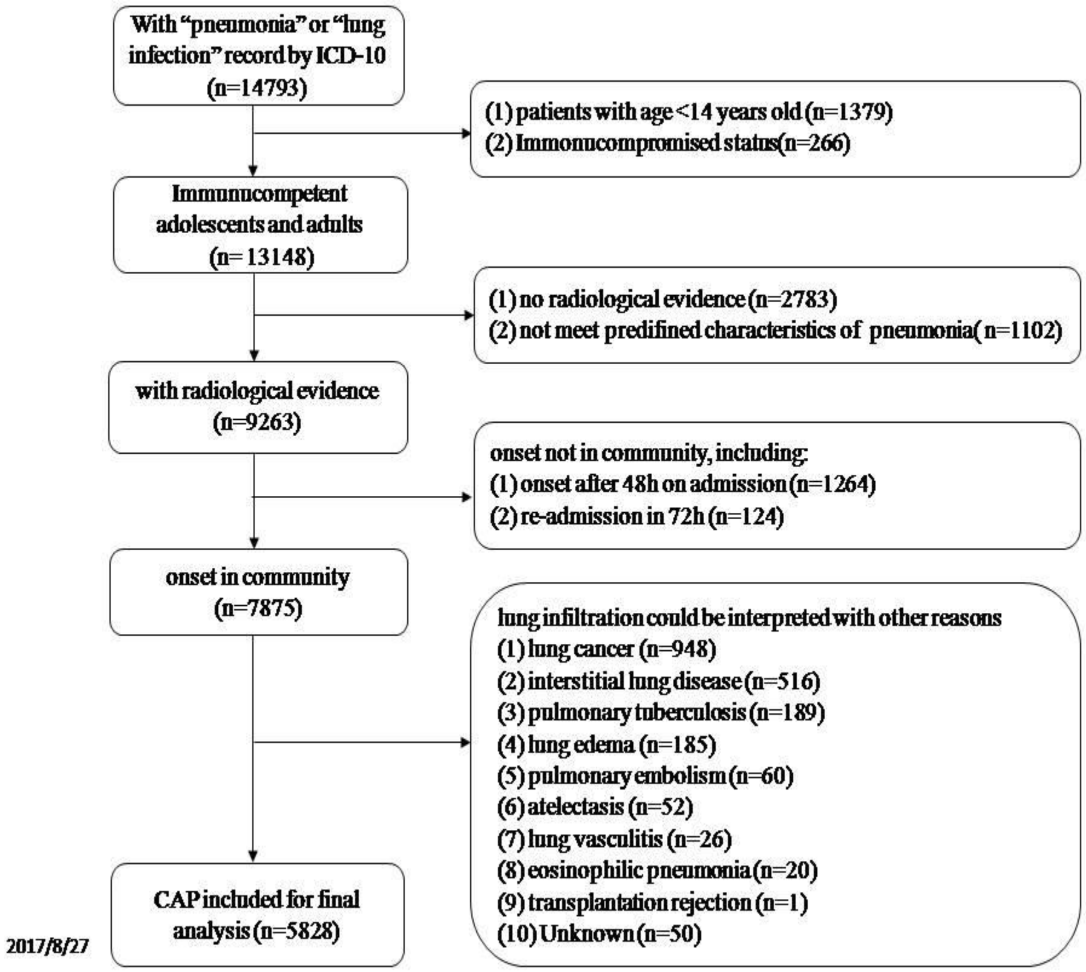

y only

For peer review only - http://bmjopen.bmj.com/site/about/guidelines.xhtml

STROBE Statement—checklist of items that should be included in reports of observational studies

	Item No	Recommendation
**Title and abstract**	1	(*a*) Indicate the study's design with a commonly used term in the title or the abstract (Page 1)
		(*b*) Provide in the abstract an informative and balanced summary of what was done and what was found (Page 3-4)
**Introduction**		
Background/rationale	2	Explain the scientific background and rationale for the investigation being reported (Page 4-5)
Objectives	3	State specific objectives, including any prespecified hypotheses (Page 5)
**Methods**		
Study design	4	Present key elements of study design early in the paper (Page 6)
Setting	5	Describe the setting, locations, and relevant dates, including periods of recruitment, exposure, follow-up, and data collection (Page 6-9)
Participants	6	(*a*) *Cohort study*—Give the eligibility criteria, and the sources and methods of selection of participants. Describe methods of follow-up<br>*Case-control study*—Give the eligibility criteria, and the sources and methods of case ascertainment and control selection. Give the rationale for the choice of cases and controls<br>*Cross-sectional study*—Give the eligibility criteria, and the sources and methods of selection of participants (Page 6-7)
		(*b*) *Cohort study*—For matched studies, give matching criteria and number of exposed and unexposed<br>*Case-control study*—For matched studies, give matching criteria and the number of controls per case
Variables	7	Clearly define all outcomes, exposures, predictors, potential confounders, and effect modifiers. Give diagnostic criteria, if applicable (Page 7-10)
Data sources/ measurement	8*	For each variable of interest, give sources of data and details of methods of assessment (measurement). Describe comparability of assessment methods if there is more than one group (Page6- 7)
Bias	9	Describe any efforts to address potential sources of bias (Page 7)
Study size	10	Explain how the study size was arrived at (Page9)
Quantitative variables	11	Explain how quantitative variables were handled in the analyses. If applicable, describe which groupings were chosen and why (Page9-10)
Statistical methods	12	(*a*) Describe all statistical methods, including those used to control for confounding (Page 9-10)
		(*b*) Describe any methods used to examine subgroups and interactions (Page 9-10)
		(*c*) Explain how missing data were addressed (Page 9)
		(*d*) *Cohort study*—If applicable, explain how loss to follow-up was addressed<br>*Case-control study*—If applicable, explain how matching of cases and controls was addressed<br>*Cross-sectional study*—If applicable, describe analytical methods taking account of sampling strategy(Page 9-10)
		(*e*) Describe any sensitivity analyses (Page 9-10)

Continued on next page

For peer review only - http://bmjopen.bmj.com/site/about/guidelines.xhtml

**Results**		
Participants	13*	(a) Report numbers of individuals at each stage of study—eg numbers potentially eligible, examined for eligibility, confirmed eligible, included in the study, completing follow-up, and analysed (Page 10)
		(b) Give reasons for non-participation at each stage (Page 10, Appendix figure 1)
		(c) Consider use of a flow diagram (Appendix figure 1)
Descriptive data	14*	(a) Give characteristics of study participants (eg demographic, clinical, social) and information on exposures and potential confounders (Page 10-11)
		(b) Indicate number of participants with missing data for each variable of interest (Table 1-5)
		(c) *Cohort study*—Summarise follow-up time (eg, average and total amount)
Outcome data	15*	*Cohort study*—Report numbers of outcome events or summary measures over time
		Case-control study—Report numbers in each exposure category, or summary measures of exposure
		Cross-sectional study—Report numbers of outcome events or summary measures (Page 10-15)
Main results	16	(*a*) Give unadjusted estimates and, if applicable, confounder-adjusted estimates and their precision (eg, 95% confidence interval). Make clear which confounders were adjusted for and why they were included (Page 10-15)
		(*b*) Report category boundaries when continuous variables were categorized (Page 11)
		(*c*) If relevant, consider translating estimates of relative risk into absolute risk for a meaningful time period
Other analyses	17	Report other analyses done—eg analyses of subgroups and interactions, and sensitivity analyses(Appendix 8)
**Discussion**		
Key results	18	Summarise key results with reference to study objectives (Page 20-23)
Limitations	19	Discuss limitations of the study, taking into account sources of potential bias or imprecision. Discuss both direction and magnitude of any potential bias (Page 23-24)
Interpretation	20	Give a cautious overall interpretation of results considering objectives, limitations, multiplicity of analyses, results from similar studies, and other relevant evidence (Page20- 23)
Generalisability	21	Discuss the generalisability (external validity) of the study results (Page24)
**Other information**		
Funding	22	Give the source of funding and the role of the funders for the present study and, if applicable, for the original study on which the present article is based (Page25)

*Give information separately for cases and controls in case-control studies and, if applicable, for exposed and unexposed groups in cohort and cross-sectional studies.

Note: An Explanation and Elaboration article discusses each checklist item and gives methodological background and published examples of transparent reporting. The STROBE checklist is best used in conjunction with this article (freely available on the Web sites of PLoS Medicine at http://www.plosmedicine.org/, Annals of Internal Medicine at http://www.annals.org/, and Epidemiology at http://www.epidem.com/). Information on the STROBE Initiative is available at www.strobe-statement.org.

For peer review only
